# Emergent actin flows explain distinct modes of gliding motility

**DOI:** 10.1038/s41567-024-02652-4

**Published:** 2024-10-08

**Authors:** Christina L. Hueschen, Li-av Segev-Zarko, Jian-Hua Chen, Mark A. LeGros, Carolyn A. Larabell, John C. Boothroyd, Rob Phillips, Alexander R. Dunn

**Affiliations:** 1 Dept. of Chemical Engineering, Stanford University, Palo Alto, CA USA; 2 Dept. of Microbiology and Immunology, Stanford University, Palo Alto, CA USA; 3 Dept. of Anatomy, University of California San Francisco, San Francisco, CA USA; 4 National Center for X-ray Tomography, Lawrence Berkeley National Laboratory, Berkeley, CA USA; 5 Molecular Biophysics and Integrated Bioimaging Division, Lawrence Berkeley National Laboratory, Berkeley, CA USA; 6 Dept. of Physics, California Institute of Technology, Pasadena, CA USA; 7 Div. of Biology and Biological Engineering, California Institute of Technology, Pasadena, CA USA; 8 Present Address: Dept. of Cell and Developmental Biology, University of California San Diego, La Jolla, CA USA

**Keywords:** Cellular motility, Biological physics

## Abstract

During host infection, *Toxoplasma gondii* and related unicellular parasites move using gliding, which differs fundamentally from other known mechanisms of eukaryotic cell motility. Gliding is thought to be powered by a thin layer of flowing filamentous (F)-actin sandwiched between the plasma membrane and a myosin-covered inner membrane complex. How this surface actin layer drives the various gliding modes observed in experiments—helical, circular, twirling and patch, pendulum or rolling—is unclear. Here we suggest that F-actin flows arise through self-organization and develop a continuum model of emergent F-actin flow within the confines provided by *Toxoplasma* geometry. In the presence of F-actin turnover, our model predicts the emergence of a steady-state mode in which actin transport is largely directed rearward. Removing F-actin turnover leads to actin patches that recirculate up and down the cell, which we observe experimentally for drug-stabilized actin bundles in live *Toxoplasma gondii* parasites. These distinct self-organized actin states can account for observed gliding modes, illustrating how different forms of gliding motility can emerge as an intrinsic consequence of the self-organizing properties of F-actin flow in a confined geometry.

## Abstract (web summary)

Unicellular parasites, such as *Toxoplasma gondii*, can use different forms of gliding motions when infecting a host. These motility modes arise from the self-organizing properties of filamentous actin flow at the surface of these parasitic cells.

## Main

Single-celled parasites of the eukaryotic phylum Apicomplexa cause hundreds of millions of cases of malaria, toxoplasmosis and cryptosporidiosis each year^1–3^. To propel themselves over host cells and through extracellular matrix, motile Apicomplexa like *Plasmodium* spp. or *Toxoplasma gondii* use an adhesion-dependent locomotion mechanism called gliding that defies the paradigmatic classification of eukaryotic cells into cilia-dependent swimmers and cell-shape-change-dependent crawlers. Gliding depends on a layer of filamentous (F)-actin^4,5^ and a fast, single-headed myosin, MyoA^6^, confined to a 25-nm-thick compartment between the parasite plasma membrane and a membranous scaffold termed the inner membrane complex (IMC)^7^. MyoA is anchored in the IMC through its association with myosin light chain 1 (MLC1)^7,8^. To drive gliding, MyoA is believed to slide short actin filaments rearward through the intermembrane space, towards the posterior end of the cell (reviewed in refs. ^9,10^). When actin-coupled adhesin proteins in the plasma membrane bind to a stationary external substrate, MyoA instead propels the inner cytoskeleton and parasite cytoplasm forward. The rearward direction of actin filament transport by MyoA was thought to be fixed and likely templated by a basket of polarized subpellicular microtubules beneath the IMC^9^. However, as discussed below, this ‘templating’ cannot account for all observed apicomplexan gliding motions.

On a two-dimensional substrate, motile *Toxoplasma gondii* parasites can undergo helical gliding, with simultaneous forward translation and cell body rotation^11^ (Supplementary Video 1), or glide in circles with their anterior (apical) end leading, motions that translate into a corkscrew trajectory when embedded in three-dimensional (3D) matrix^12–14^. These forward cell movements contributed to the working model of rearward actin transport along the path of subpellicular microtubules^9^. However, parasites also display a rotational motion known as twirling when oriented upright, with their posterior end on the substrate. In addition, observations of back-and-forth motion, termed patch or pendulum gliding, have been reported in a diversity of conditions (Supplementary Table 1, Supplementary Fig. 1a,b and Supplementary Video 1). Prevalent models of rearward-only actin transport cannot explain these motions. Further, MLC1 localizes throughout the IMC, not merely above subpellicular microtubules^15,16^ (Supplementary Fig. 1c), making it unclear how myosin orientation might be fixed relative to the cell axis. Recent observations of unchanged gliding speeds in *Toxplasma gondii* mutants with short and sparse microtubules^17^, the discovery of gliding by *Plasmodium* merozoites^18^, which do not have a basket of chiral subpellicular microtubules, and the observation of actin filament alignment to newly described IMC surface filaments in *Cryptosporidium parvum*^19^ further suggest that our understanding of how gliding motility arises from molecular-level organization remains incomplete. In this study, we sought to better understand how the actomyosin machinery gives rise to such a diverse array of gliding movements and, more broadly, how actin and myosin are patterned or polarized to yield coherent force generation in this system.

## Tracking actin and myosin in motile *Toxoplasma gondii*

**Fig. 1:**
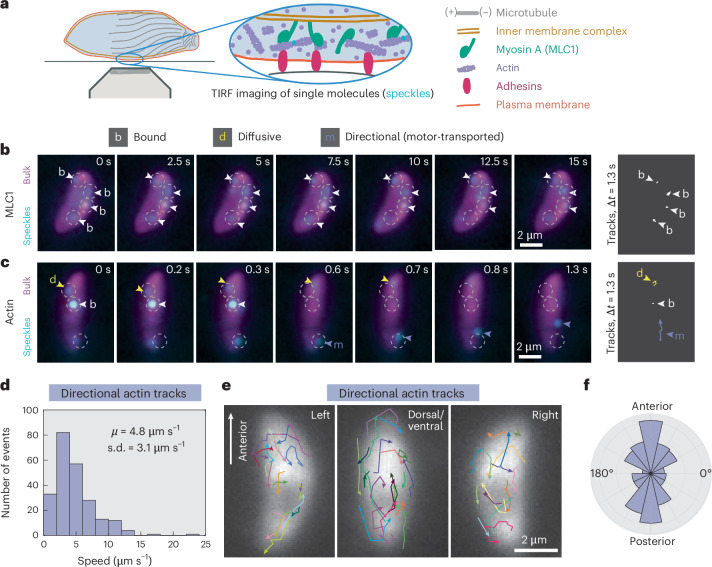
*Toxoplasma gondii* actin transport direction is heterogeneous, not uniformly rearward. **a**, Schematic of the intermembrane actomyosin layer that drives apicomplexan gliding and of TIRF imaging of speckle-labelled actin or MLC1. Inset: actin is speckle-labelled with single Janelia Fluor dyes (cyan). **b**,**c**, Examples of MLC1 (**b**) and actin (**c**) movement (speckles; cyan) over time in extracellular parasites, with higher-density labelling (bulk; magenta) to show cell position. Arrowheads highlight examples of specific protein behaviours. Images denoised with noise2void^60^. Protein trajectories (far right) are shown for an equal time interval (1.3 s) to allow comparison of bound (white), diffusive (yellow) and directional (purple) movements. Experiment repeated in *n* = 7 (MLC1) and *n* = 18 (actin) independent cells. **d**, Histogram of speeds of directional actin tracks from 18 cells. *μ*, mean; s.d., standard deviation. **e**, Directional actin tracks (*n* = 54 tracks in 18 overlaid cells from three experiments). Cells were grouped by the cell side visible, aligned with anterior end up and superimposed. Cell polarity was determined by microtubule labelling or tracking of the posterior end following posterior-down cell twirling (Supplementary Information Section 5), and directional actin tracks were aligned with respect to the parasite long axis. **f**, Polar histogram of the orientation of directional actin displacements with respect to cell polarity (*n* = 231 displacements, 54 tracks, 18 cells).

To directly test the model of ‘templated’ rearward actin transport along the path of subpellicular microtubules, we used fluorescent speckle imaging^20,21^ to track actin (ACT1) and MLC1 proteins labelled with individual fluorophores in live, active *Toxoplasma gondii* tachyzoites (Fig. 1a). Pauses between parasite movements enabled us to reliably track protein movement (Methods) relative to microtubule polarity, which is fixed through the tachyzoite life stage. MLC1 proteins were frequently immobile (‘bound’) for tens of seconds (Fig. 1b, Supplementary Fig. 2a and Supplementary Video 2), consistent with a stably anchored population of MyoA motors at the IMC. Relative to MLC1, a larger fraction of actin was mobile (Supplementary Fig. 2b) and displayed meandering (‘diffusive’) behaviour as well as ballistic (‘directional’) behaviour consistent with processive transport of filaments by myosin (Fig. 1c and Supplementary Video 3). Directional actin moved with a mean speed of 4.8 µm s^−1^ (Fig. 1d), similar to the in vitro actin transport speeds of 4–5 µm s^−1^ reported for purified *Toxoplasma* MyoA complexes^22,23^.

Analysis of directional actin tracks in live parasites was inconsistent with a mechanistic model featuring fixed myosin polarity and uniformly rearward actin flow. Relative to the parasite long axis, F-actin transport direction was heterogeneous and as often forward as rearward (Fig. 1e,f). Although the orientation of subpellicular microtubules or IMC surface filaments could contribute to the observed bias towards longitudinal F-actin transport (Fig. 1f), for example by steric channelling^19^ or by scaffolding closer longitudinal than latitudinal MyoA spacing^24^, microtubule polarity evidently did not template rearward-only actin transport.

## A theoretical model of actin filament collective motion

**Fig. 2:**
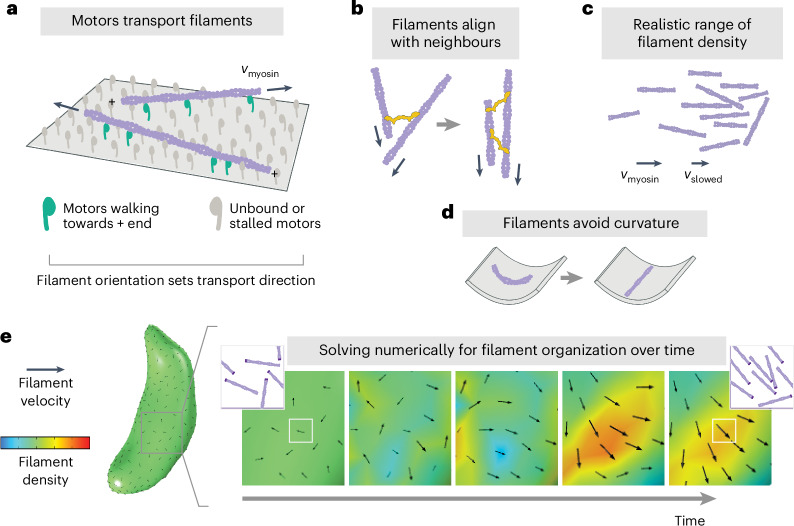
*Toxoplasma* actin self-organization: theoretical model. Rules of local actin filament behaviour, implemented in equation (2): **a**, Actin filaments (purple) are transported with their minus ends leading at speed *v*_myosin_, as indicated by arrows. **b**, Neighbouring filaments align by steric effects or crosslinking proteins (yellow). **c**, Filament density remains within a realistic range, with filament speed slowing if entering a pile-up. **d**, Filament orientation is biased towards lower curvature. **e**, Example of numerically solving for filament self-organization using the finite element method, predicting filament density and velocity over time. Black arrow size reflects velocity magnitude. To provide intuition, for each white box, the corresponding inset shows schematized F-actin (darker purple represents minus ends) whose density and orientation are consistent with the simulation density (colour) and velocity (black arrow).

Our data, combined with the diversity of gliding modes exhibited by motile Apicomplexa, led us to explore the possibility that gliding motility might represent an emergent self-organized state rather than the consequence of a microtubule-templated asymmetry in actomyosin polarity. In this scenario, the heterogenous F-actin transport observed between glides (Fig. 1d–f) could reflect a disorganized state between transient self-organized actin states that drive gliding. Self-organization^25^ is a hallmark of actomyosin networks, with morphologically diverse examples such as the lamellipodia of crawling keratocytes^26^ and neutrophils^27^, the flowing cortex of *C. elegans* zygotes^28^, and dense actin networks of in vitro motility systems^29^. Drawing on continuum theories for active collective motion or flocking^30–32^ and previous studies of self-organization of cytoskeletal systems^28,33,34^, we developed a continuum model of *Toxoplasma* actin filament collective motion (Fig. 2 and Supplementary Fig. 3). In our model, actin filaments at the surface of the cell follow a few simple rules: filaments are transported at the speed of myosin motors, and filament orientation sets the myosin-driven transport direction (Fig. 2a); filaments align with neighbouring filaments through collisions^35^ or due to crosslinking proteins like *Toxoplasma* coronin^36–38^ (Fig. 2b); filament density remains within a realistic range (Fig. 2c); and filament alignment is biased away from orientations of high membrane curvature (Fig. 2d). Actin filament organization over space and time (*t*) is described by two fields: the scalar field *ρ*, which captures filament density, and the velocity vector field **v**, which captures both filament polarity (orientation of **v**) and speed (magnitude of **v**). Two equations are needed: the continuity equation

1 $${\frac{\partial \rho }{\partial t}}+\nabla \bullet \left(\,\rho {\bf{v}}\right)=0$$

ensures conservation of filaments, and filament velocity evolves according to the rules described above using the minimal Toner–Tu equations

2 $$\begin{array}{ll} {\displaystyle \frac{\partial {\bf{v}}}{\partial t}} \normalsize = -{\rm{\lambda }}{\bf{v}}\bullet \nabla {\bf{v}} + \left(\alpha \left(\,\rho -{\rho }_{{\mathrm{c}}}\right)-\beta {\left|{\bf{v}}\right|}^{2}\right){\bf{v}}\\\qquad + \,D{\nabla }^{2}{\bf{v}} - \sigma \nabla \rho - \varepsilon {F}_{\rm{K}}\left({\bf{v}}{,}{{\kappa }}\right)\left({{I}}-\hat{{\bf{v}}}\otimes \hat{{\bf{v}}}\right){{\bf{d}}}_{{\boldsymbol{1}}}\end{array}$$

where *ρ*_c_ is the critical density above which filaments move coherently, the coefficient *λ* tunes filament transport (self-advection), the ratio of *α* and *β* sets a filament transport speed scale, *D* tunes filament alignment with neighbours, ${\rm{\sigma }}\nabla \rho$ provides an effective pressure that limits density variance, $\hat{{\bf{v}}}$ is a unit vector in direction **v**, *I* is the identity matrix, and the coefficient *ε* tunes the curvature (${{\kappa }}$)-induced force *F*_κ_(**v**, ${{\kappa }}$) that rotates filaments away from **d**_1_, the direction of maximum curvature (Supplementary Fig. 3). This final term generally favours filament alignment to the cell long axis and could also capture, conceptually, an orientation bias from the subpellicular microtubules or IMC surface filaments^19^. The exclusion of any of these terms leads to results that are either unphysical or inconsistent with established *Toxoplasma* biology (Supplementary Information Section 6). Although likely a simplification, the generality of this minimal flocking framework allowed us to explore how cell-scale actin organization might emerge from local actomyosin interactions confined to *Toxoplasma*’s surface shape.

**Fig. 3:**
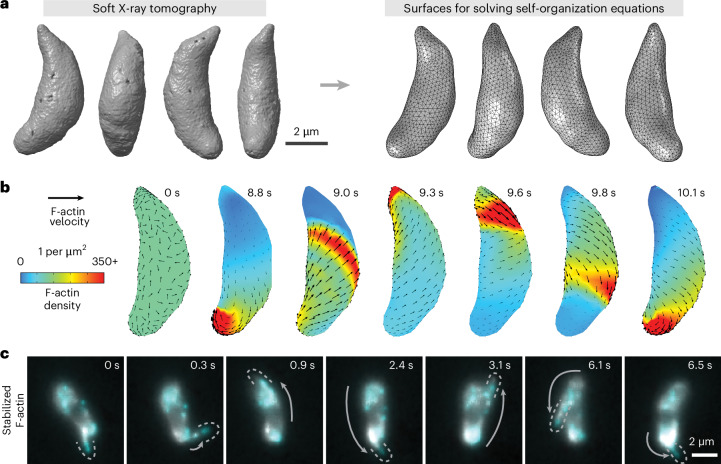
Stable actin filaments circle the *Toxoplasma* cell. **a**, Soft X-ray tomograms of cryo-fixed extracellular *Toxoplasma gondii* tachyzoites were used to generate triangle-meshed surfaces on which to solve our actin self-organization theoretical model. **b**, For stable filaments, solving the model constrained to *Toxoplasma*’s surface geometry predicts recirculating actin patches. **c**, In experiments, actin filaments briefly stabilized with jasplakinolide can circle around the cell. Cyan and grey both show actin, labelled at different dye densities. Images denoised with noise2void^60^. Dotted lines outline protruding actin filaments, and grey arrows highlight the movement of the protrusion since the previous frame. Representative of *n* = 10 cells whose protrusion velocities are characterized in Supplementary Fig. 4.

To predict what cell-scale actin organization patterns could emerge from the molecule-scale rules illustrated in Fig. 2, we began with a disordered network and asked how filament density and velocity evolve over time (Fig. 2e), using the finite element method to solve equations (1) and (2) in COMSOL Multiphysics. Importantly, we sought to incorporate the true shape of this thin membrane-constrained layer of actin, which can be approximated as a two-dimensional closed surface following the rigid and stereotypical shape of *Toxoplasma gondii* tachyzoites. We used soft X-ray tomography^39^ to obtain native-state high-resolution 3D reconstructions of cryo-fixed extracellular parasites and used a spherical harmonic description^40^ to convert them to closed surfaces for finite element analysis (Fig. 3a). We then derived a curved-surface formulation of our governing equations (1) and (2) using extrinsic differential geometry^41–43^ (Supplementary Information Section 8). In essence, the resulting formulation uses the surface normal vector to project into the local tangent plane and thus requires no intrinsic surface parameterization. We note the versatility of such an approach for solving continuum models on complex geometries for both living and non-living systems^42,44^.

## A transition from recirculation to unidirectional actin transport

Starting from a disordered initial state and, importantly, the assumption of a conserved number of stable actin filaments enforced by equation (1), numerical simulations predicted the emergence of patches of parallel actin filaments that circulate up and down along the cell as shown in Fig. 3b and Supplementary Video 4. We observed a similar recirculation of F-actin in experiments, imaging actin bundles in *Toxoplasma* tachyzoites treated briefly with the actin-stabilizing drug jasplakinolide (Fig. 3c and Supplementary Video 5). Actin bundles repeatedly circled the cell at a mean speed of 5.7 µm s^−1^ ± 3.2 s.d. (Supplementary Fig. 4). Thus, in the absence of filament turnover, a self-organization model predicts the emergence of F-actin recirculation: a continuous ‘cyclosis’ observed experimentally for stabilized filaments in live parasites.

Next, we extended these theoretical and experimental results to consider regimes of filament turnover. Importantly, *Toxoplasma* helical and circular gliding modes are known to require regulated actin depolymerization by proteins like profilin^45^ and actin depolymerizing factor (ADF)^46^. Further, the polymerization of F-actin essential to gliding depends on formin 1 (FRM1), which localizes to the parasite anterior^47–49^. Estimates of F-actin lifetime and the characteristic timescale of F-actin cyclosis are both on the order of seconds, justifying an addition of filament turnover to our theoretical model that extends it beyond prior flocking models^50^ (Supplementary Information Section 6.4). Based on current knowledge, polymerization is favoured specifically at the cell anterior, whereas depolymerization by profilin and ADF is not known to be spatially restricted. Thus, in the filament turnover model, F-actin density at the anterior cell surface is governed by

3 $$\frac{\partial \rho }{\partial t}+\nabla \bullet \left(\,\rho {\bf{v}}\right)={c}-{\gamma }\rho$$

where *c* tunes F-actin polymerization and stabilization (rate of filaments produced per unit area) and *γ* tunes depolymerization (rate of filament loss). Outside the cell anterior (Supplementary Fig. 4), F-actin density is governed by

4 $$\frac{\partial \rho }{\partial t}+\nabla \bullet \left(\rho {\bf{v}}\right)=-{\gamma}{\rho}$$

while F-actin velocity across the entirety of the cell is governed by equation (2).

**Fig. 4:**
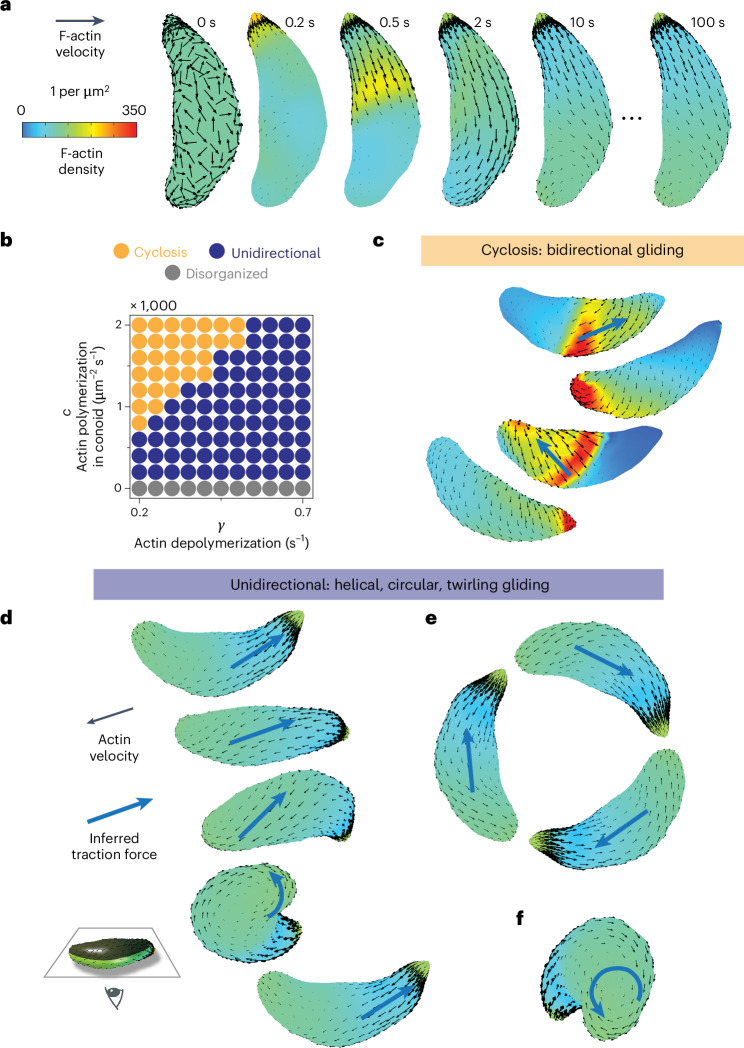
Polarized actin turnover governs a transition between actin recirculation and unidirectional transport. **a**, Incorporating F-actin depolymerization and anterior polymerization into the model enables the emergence of a unidirectional, stable velocity pattern. **b**, Tuning rates of F-actin polymerization and depolymerization move the cell between distinct self-organized states: bidirectional cyclosis, unidirectional and disorganized. **c**, Model prediction: recirculating F-actin cyclosis generates bidirectional traction force (blue arrows) to drive ‘patch’ gliding. **d**–**f**, Model prediction: the unidirectional self-organized F-actin state drives helical gliding (**d**), circular gliding (**e**) and twirling (**f**). In the images in **c**–**f**, cells are viewed from below; cell-substrate contact occurs at the position of the inferred traction force.

The addition of F-actin turnover and anterior polymerization enabled the emergence of a new F-actin organization state, in which actin transport is largely unidirectional and rearward (Fig. 4a and Supplementary Video 6). In this emergent state, filament density and velocity reach a steady state: whereas individual actin filaments flow continuously rearward, the average F-actin density and velocity at a given position reaches a fixed value. The asymmetric *Toxoplasma* cell shape was necessary for the emergence of this steady state and its consistent chirality (Supplementary Fig. 5). Tuning filament polymerization and depolymerization rates (Fig. 4b) shifted the emergent F-actin pattern between states. Increasing anterior polymerization and increasing filament stability (lowering depolymerization rate) favoured the F-actin cyclosis described in Fig. 3. Conversely, increasing filament depolymerization rate favoured unidirectional F-actin transport. At an intuitive level, the transition from cyclosis to unidirectional flow occurs as filament lifetime (1/*γ*) drops below the cross-cell filament transport time (~*L*_cell_/*v*_myosin_), preventing a posterior pile-up of F-actin large enough to force recirculation. In summary, actin turnover governs a transition between two self-organized states: F-actin recirculation and steady-state unidirectional transport.

## Self-organized actin states can account for gliding behaviours

In this section, we develop the working hypothesis that actin self-organization into distinct states explains the rich diversity of apicomplexan cell movements observed experimentally, from helical gliding and twirling to back-and-forth patch gliding^5,11,51^. During gliding on a surface, parasites form an adhesive cell-substrate contact point, where adhesin proteins bind the external substrate and form a stationary patch^48,52^. Cell motion occurs when MyoA walks the inner cytoskeleton and parasite cytoplasm towards the plus ends of F-actin adhering to that stationary patch^9,22^. Therefore, local F-actin polarity dictates the direction of myosin-powered traction force and the direction of cell movement (Supplementary Fig. 6). A map of self-organized actin velocity (Figs. 3b and 4a) thus implies a corresponding map of traction force direction (blue arrows, Fig. 4c–e).

In the recirculating actin state, a qualitative translation of predicted F-actin velocity patterns into traction force orientation (blue arrows, Fig. 4c) can explain the previously puzzling observations of back-and-forth *Toxoplasma* and *Plasmodium* cell gliding summarized in Supplementary Table 1. These observations include patch gliding, pendulum gliding and rolling in conditions like *Toxoplasma gondii* actin depolymerization factor (ADF) knockout cells^46^, *Toxoplasma gondii* treated with actin stabilizers^5^ and *Plasmodium berghei* sporozoites with mutations in the actin-binding adhesin protein TRAP^51,53^ or in actin itself^54,55^. Our theoretical finding that increased filament stability shifts F-actin self-organization from a unidirectional to recirculating mode (Fig. 4b) provides a unifying interpretation of these disparate experimental results (Supplementary Table 1).

In the unidirectional regime, tuning the rate of F-actin depolymerization changes features of the predicted velocity patterns, including chirality and density gradient length scale (Supplementary Fig. 7). For choices of polymerization and depolymerization rate close to the unidirectional-to-recirculating transition, emergent F-actin velocity patterns are consistent with the observed mechanics of helical gliding, circular gliding and twirling (Fig. 4d–f). Helical gliding initiates when the ‘left’ side of the cell is in contact with the substrate, whereas circular gliding initiates given ‘right’ side contact (considering the concave cell surface to be ventral)^11^. In each case, inferred traction force vectors along the path of substrate contact can qualitatively explain observed cell translation and rotation (Fig. 4d,e). Similarly, predicted vortical F-actin polarity at the parasite posterior would lead to a myosin-powered torque and cell rotation or twirling, which is indeed characteristic during cell posterior contact (Fig. 4f). Thus, we hypothesize that the cyclosis mode of F-actin self-organization (Figs. 3b and 4c) drives bidirectional cell gliding (patch, pendulum, rolling); the unidirectional mode of F-actin self-organization (Fig. 4a,d–f) drives helical gliding, circular gliding and twirling; and actin turnover governs the transition between modes. Indeed, decreasing F-actin turnover through titrated jasplakinolide treatment led to more frequent bidirectional gliding and less unidirectional gliding (Supplementary Fig. 8a) but did not change the fractional breakdown of unidirectional gliding into helical, circular and twirling movements (Supplementary Fig. 8b).

We hope that the theoretical and experimental results presented here will prove a stimulating first step in understanding actomyosin self-organization in the Apicomplexa, given the fruitfulness of the self-organization paradigm as a null hypothesis for cytoskeletal systems across biology. Far from wishing to claim finality for the particulars of the model developed here, we look forward to the incorporation and discovery of additional biological complexity through a dialogue between theory and experiment. Such a dialogue will benefit from fast and sensitive volumetric imaging of actin single molecules, combined with sufficiently sophisticated analysis algorithms, to enable 3D reconstructions of F-actin velocity fields in the reference frame of the cell during specific gliding motions and from quantitative comparison of predicted and measured traction forces and gliding mechanics^51,52,56^.

Broadly, we note continuum theory’s ability to unify natural phenomena across scales, allowing a flocking theory inspired by collective bird motion^30,32^ to provide insight into microscopic actin organization in a unicellular parasite. Looking forward, the mathematical framework developed here will enable a meaningful examination of actomyosin self-organization in *Plasmodium* spp. sporozoites, ookinetes and other motile Apicomplexa, incorporating their characteristic cell shapes to generate self-organized patterns of actin velocity and inferred traction force for comparison to experimental data. Further, reports of gliding cells exist within at least three major clades of eukaryotic life^57,58^, suggesting that this ‘esoteric’ mode of cell locomotion may in fact be common but understudied and deserving of a unifying effort^59^ to understand its physical principles and their degree of conservation across Eukarya.

## Methods

### Parasite and host cell culture

*Toxoplasma gondii* type I RH tachyzoites were maintained by serial passage in primary human foreskin fibroblasts (HFFs) in Dulbeccos modified Eagles high glucose medium (DMEM; Gibco 11960-044) with 10% heat- inactivated foetal bovine serum (FBS; Corning 35-011-CV), 2 mM glutamine (Sigma-Aldrich G7513), 100 U ml^−1^ penicillin and 100 μg ml^−1^ streptomycin (Gibco 15140122) at 37 °C in 5% CO_2_. In brief, to passage parasites, infected HFF monolayers were suspended in media by scraping, syringe lysed using a 25-gauge blunt-end needle (SAI Technologies B25-50) and added at a 150-fold dilution to an confluent uninfected HFF monolayer, every 2–4 days. HFFs were obtained from the neonatal clinic at Stanford University following routine circumcisions that are performed at the request of the parents for cultural, health or other personal medical reasons (that is, not in any way related to research). These foreskins, which would otherwise be discarded, were fully deidentified and therefore do not constitute human subjects research. Uninfected HFFs were maintained in the supplemented DMEM described above, passaged using 0.25% trypsin-EDTA (Gibco 25200056) and discarded after passage 15.

### Generation of halo-ACT1 and MLC1-halo strains

In brief, halo-TgACT1 or TgMLC1-halo fusions under the control of a weak promoter (from TGGT1 239010, gift of M. Panas^61^) were incorporated into the genome of the *Toxoplasma gondii* type I RH ∆hxgprt ∆ku80 strain^62^. In detail, p239010-halo-C1-HXGPRT and p239010-halo-N1-HXGPRT vectors were created by replacing a region of the pGRA-3xHA-HPT vector^63^ (from the pGRA promoter through the translated region) with (1) the TGGT1 239010 promoter and 5′ UTR to drive low expression levels, (2) the HaloTag sequence (Promega) and (3) a serine-glycine linker and multiple cloning site either C-terminal (‘C1’) or N-terminal (‘N1’) to the HaloTag sequence. TgACT1 (TGGT1 209030) or TgMLC1 (TGGT1 257680) was then synthesized and cloned into the p239010-halo-C1-HXGPRT or p239010-halo-N1-HXGPRT vector, respectively (Epoch Life Science, Inc.). For transfection with p239010-halo-ACT1-HXGPRT or p239010-MLC1-halo-HXGPRT, RH ∆hxgprt ∆ku80 parasites were mechanically released in phosphate-buffered saline (PBS), pelleted and resuspended in 20 μl P3 primary cell Nucleofector solution (Lonza) with 15 μg DNA and electroporated using the Amaxa 4D Nucleofector (Lonza). Transfected parasites were permitted to infect and grow in confluent HFFs for 48 h, after which time the media was supplemented with 50 μg ml^−1^ mycophenolic acid and 50 μg ml^−1^ xanthine for HXGPRT selection. Parasites were passaged four times over 10–12 days in selection media before being singly cloned into 96-well plates by limiting dilution. Clones were expanded and screened for HaloTag expression by incubating intracellular parasites overnight with 50 nM TMR HaloTag Ligand (Promega G8251), washing 5× with PBS to remove unbound dye, fixing with 4% paraformaldehyde (EMS AA433689M) for 15 min and imaging fluorescence.

### Single-molecule (speckle) and bulk labelling in live parasites

Before infected HFFs were lysed to release parasites for imaging, parasites within infected HFF monolayers were labelled for 3 h at 37 °C with Janelia Fluor 549 HaloTag Ligand (Promega GA1110) and Janelia Fluor 646 HaloTag Ligand (Promega GA1120)^20^ at a concentration of 1–10 pM for single-molecule imaging or 100–500 pM for bulk population imaging. For example, the MLC1-halo parasites shown in Fig. 1 show MLC1 labelled with 10 pM (cyan speckles) and at 200 pM (magenta bulk population of MLC1, showing the shape of the cell). Because Janelia Fluor dyes bleach over time in storage, dye concentration must be optimized empirically and adjusted on the timescale of months; furthermore, care should be taken to avoid more than two or three freeze–thaw cycles before use. When subpellicular microtubule imaging was used to determine parasite polarity, infected HFFs were labelled with 100 nM siR-tubulin and 10 μM verapamil (Cytoskeleton, Inc. CY-SC002) alongside 1–10 pM Janelia Fluor 549. Before parasite release, infected HFF monolayers were washed 7× with DMEM to ensure removal of unbound dye.

### Preparation and TIRF imaging of live extracellular parasites

To release parasites, infected HFFs were scraped and syringe lysed in fresh phenol red-free DMEM with a 27-gauge needle (SAI Technologies B27-50). Freshly released parasites were placed on 35 mm #1.5 glass-bottomed dishes (Cellvis D35-20-1.5-N; incubated with 10% FBS before use) with a confluent monolayer of HFFs grown on Snapwell Insert polyester membranes (Corning Costar CLS3801) suspended approximately 0.2 mm above them. Parasites were imaged at 30 °C using objective-type total internal reflection fluorescence (TIRF) microscopy on an inverted microscope (Nikon Ti-E) with a heated Apo TIRF 100 oil objective of numerical aperture 1.49 (Nikon) and controlled using Micro-Manager v.1.4 (ref. ^64^). To enable simultaneous two-colour imaging, samples were excited with both 532 nm (Crystalaser) and 635 nm (Blue Sky Research) lasers and emitted light passed through a quad-edge laser-flat dichroic with centre/bandwidths of 405 nm/60 nm, 488 nm/100 nm, 532 nm/100 nm and 635 nm/100 nm from Semrock (Di01-R405/488/532/635-25×36) and corresponding quad-pass filter with centre/bandwidths of 446 nm/30 nm, 510 nm/30 nm, 581 nm/30 nm, 703 nm/30 nm band-pass filter (FF01-446/510/581/703-25). Emission channels were then separated as previously described^65^ and recorded on an electron-multiplying charge-coupled device camera (Andor iXon).

### Frequency of gliding modes with titrated actin stabilization by jasplakinolide (low concentrations)

Live extracellular parasites were prepared as in the preceding section and added to microgrids of 75 μm × 75 μm square PDMS wells (Microsurfaces MGA-075-02) in glass-bottomed 24-well plates. Upon addition of 650,000 parasites per well, plates were spun at 100 g for 3 min to settled parasites into grids. For jasplakinolide experiments, the indicated concentrations (Supplementary Fig. 8) of jasplakinolide (Millipore Sigma J4580) were then added and mixed by pipetting. Imaging began 40–50 min later. Multiple stage positions were imaged per condition, and microgrid walls prevented shear stress or parasite detachment from sloshing of imaging media. Brightfield images were acquired at a frame rate of five frames per second at 37 °C in 5% CO_2_ using a Nikon Ti-E inverted microscope with a 20×/0.5 numerical aperture Plan Fluor CFI air objective and an Andor Neo camera. Image acquisition was controlled using Micro-Manager software^64^.

For the fraction of cells in each motility mode reported in Supplementary Fig. 8, means were calculated by ‘pooling’ all measurements of motility events from three independent experiments. This is equivalent to a weighted mean, ${x}^{* }=\sum {w}_{i}{x}_{i}/\sum {w}_{i}$, where for experiment *i* the weight *w*_*i*_ is the total number of motility events measured and *x*_*i*_ is the fraction of cells in a given mode. Weighted standard deviations are calculated as $\sum {w}_{i}{({x}_{i}-{x}^{* })}^{2} / \left(\sum {w}_{i}-\frac{\sum {w}_{i}^{2}}{\sum {w}_{i}}\right)$, where the denominator is a correction to yield an unbiased estimator given ‘reliability’ weights^66^.

### Jasplakinolide treatment (high concentration) and recirculating actin bundles

Live extracellular parasites were prepared as above, with the addition of 1 μM jasplakinolide (Millipore Sigma J4580) immediately before imaging. In a narrow window of time from approximately 15 min until 30 min after jasplakinolide addition, protruding bundles of actin filaments were observed circling around the periphery of parasites (Supplementary Fig. 4). We note that this recirculating behaviour was very sensitive to treatment time and drug concentration. Over time, most protrusions lost this recirculating behaviour and became fixed in position at the anterior (apical) end, as previously observed^67^. We speculate that this transition to fixed apical actin bundles occurs as bundles grow long enough (with polymerization favoured both by jasplakinolide and by apically-localized formin 1 (ref. ^68^)) to protrude through the conoid and into the cytoplasm and can no longer re-orient to contact myosin motors on the outside of the IMC. Under ideal treatment conditions, most extracellular parasites observed displayed recirculating actin protrusions; under less ideal conditions (for example, after more than 30 min treatment or with poorly attached parasites), less than 10% of parasites displayed recirculating protrusions. We also note that to image these large recirculating bundles, we relaxed the steep angle of the excitation light and performed highly inclined and laminated optical sheet (HILO) or ‘dirty TIRF’ imaging. Thus, fluorescently labelled actin structures within the cell cytoplasm are visible in addition to the gliding-associated surface actin.

### MLC1 immunofluorescence and confocal microscopy

Parasites were released from infected HFF monolayers by scraping and syringe lysis in DMEM with a 27-gauge needle (SAI Technologies B27-50), passed through a 5 μm filter (Millipore Sigma SLSV025LS) and allowed to settle onto #1.5 coverslips at 37 °C in 5% CO_2_ for 30 min in DMEM + 1 μM calcium ionophore A23187 (Sigma C7522). Subsequent staining steps were performed at room temperature: parasites were fixed with warm 4% paraformaldehyde (EMS AA433689M) for 15 min, washed 3× with PBS, incubated with permeabilization-and-blocking buffer (0.1% Triton-X-100 and 2% bovine serum albumin in PBS) for 20 min, incubated with mouse anti-tubulin monoclonal antibody DM1α (Sigma T6199; diluted 1:500) and 2 nM Janelia Fluor 646 HaloTag Ligand (Promega GA1120) in permeabilization-and-blocking buffer for 1 h, washed 3× with PBS, incubated with anti-mouse IgG secondary antibody conjugated to Alexa Fluor 488 (Cell Signaling 4408S, diluted 1:500) for 20 min, washed 3× with PBS and mounted in ProLong Gold Antifade (ThermoFisher P36934). Samples were imaged using an inverted Zeiss LSM 780 confocal microscope with a 63X/1.4 numerical aperture oil objective, 488 nm Ar laser, 633 nm HeNe laser and Zeiss Airyscan detector (32-channel gallium arsenide phosphide photomultiplier tube (GaAsP-PMT) area detector), in which using each detector element as an individual pinhole combined with linear deconvolution achieves a spatial resolution below the diffraction limit^69^. All images were acquired using Zen v.2.3 (black edition) software (Carl Zeiss).

### Soft X-ray tomography

HFF monolayers, 18–20 h after parasite infection, were washed 2× with Hanks balanced salt solution (Gibco 14175095) supplemented with 1 mM magnesium chloride, 1 mM calcium chloride, 10 mM sodium hydrogen carbonate and 20 mM HEPES, pH 7. HFFs were scraped and passed through a 27-gauge needle (SAI Technologies B27-50) to release parasites into fresh Hanks balanced salt solution at room temperature. Calcium ionophore A23187 (Sigma C7522) at a final concentration of 1 μM was added to the sample at room temperature for 10 min. Parasites were pelleted, excess liquid was aspirated, and parasites were resuspended in the remaining liquid (~25 μl) before loading into 5-μm-diameter glass capillaries. Parasites inside capillaries were then vitrified by fast plunge-freezing in 90 K liquid propane. Capillaries were imaged using the XM-2 cryo soft X-ray microscope at the National Center for X-Ray Tomography at the Advanced Light Source (Lawrence Berkeley Laboratories). The XM-2 is equipped with a micro zone plate with a spatial resolution of 60 nm, and the imaged capillary was in an atmosphere of helium gas stream cooled by liquid nitrogen. To have a full rotated tomographic dataset reconstructed, 92 projection images were taken with 2° increments. The exposure time of each projection varied between 200 and 450 ms, depending on the beam flux and the sample thickness. Projection images were normalized and aligned, and the tomographic reconstructions were calculated using iterative reconstruction methods in the AREC-3D package^70^. Additional information on the soft X-ray tomography method is available in ref. ^71^.

### Image analysis

Speckle tracking of actin and MLC1 was done using u-track software (v.2.2.0) made available by the Danuser lab^72^ and run through MATLAB R2019a from Mathworks, Inc or by manual spot tracking of raw images with the Manual Tracking plugin^73^ within Fiji (ImageJ v.0.0-rc-69/1.52p)^74^. Full analysis details and analysis methods for Supplementary Figs. 1, 2, 4 and 8 are presented in Supplementary Information Section 4.

### Theoretical model of *Toxoplasma gondii* actin filament self-organization

To describe the collective motion and organization of actin filaments, we repurposed a classic continuum active matter model that was originally developed by John Toner and Yuhai Tu, inspired by the work of Tàmas Vicsek, to describe the collective behaviour of flocking or schooling animals^30–32^. This class of theoretical models, known as Toner–Tu or flocking theory, describe collections of ‘dry’, polar, self-propelled agents at any length scale, from flocks of flying birds to collections of polarized cytoskeletal filaments. In our case, *Toxoplasma gondii* actin filaments at the cell surface are propelled along by an underlying carpet of plus-end-directed myosin motors, whose action we can effectively capture as polarized filament self-propulsion. In Supplementary Information Sections 6 and 7, we provide a detailed explanation of the choice of our model, our equations and an intuitive interpretation for each term, a derivation of the curvature penalty term, the addition of filament polymerization and depolymerization into the model and our parameter choices. In Supplementary Information Section 8, we discuss the derivation of a tangential formulation of our filament self-organization equations using an extrinsic differential geometry approach, which is well-suited for numerical analysis using the finite element method. As discussed in detail in Supplementary Information Section 9, we used COMSOL Multiphysics^75^ to solve our self-organization equations on triangular surface meshes of *Toxoplasma gondii* created from soft X-ray images of the extracellular *Toxoplasma gondii* tachyzoite cell shape using the SPHARM-PDM 3D Slicer package^76,77^.

### Statistics and reproducibility

Sample sizes: sample sizes were not determined a priori. For each experimental condition/replicate, we checked the robustness of our measurements by cell-to-cell and experiment-to-experiment comparisons and using statistical tests, when appropriate. Randomization: cells in experimental groups were random subsets of the same cell stock. In imaging experiments, individual wells or fields of view in imaging dishes were chosen at random for each experimental group. Blinding: analysis of motility modes across jasplakinolide concentrations (Supplementary Fig. 8) was performed blind to experimental condition and in a randomized order. Data exclusion: for speckle imaging experiments, we excluded cells with a fluorescent dye labelling density that was too low or too high to visualize and distinguish individual speckles. When tracking speckle movement, we analysed tracks that persisted for at least five frames (0.43 s). Assumptions of statistical tests: statistical tests performed on data in Supplementary Figs. 2 and 8 are discussed in detail in the text associated with those figures.

### Reporting summary

Further information on research design is available in the Nature Portfolio Reporting Summary linked to this article.

## Online content

Any methods, additional references, Nature Portfolio reporting summaries, source data, extended data, supplementary information, acknowledgements, peer review information; details of author contributions and competing interests; and statements of data and code availability are available at 10.1038/s41567-024-02652-4.

## Supplementary information

Supplementary Information Supplementary Figs. 1–12, Table 1, extended methods, theoretical model, references and appendix.

Reporting Summary

Supplementary Video 1 Diverse gliding modes: an individual cell performs back-and-forth gliding, helical gliding and then twirling. In addition to recommending the classic work of Håkansson et al.^11^, we include this video to orient readers unfamiliar with gliding motility and to illustrate that an individual cell can switch between gliding modes. In this brightfield microscopy video, an extracellular *Toxoplasma gondii* tachyzoite glides back and forth (0:00 min:s), displaying so-called patch or pendulum gliding (Supplementary Table 1). Several minutes later, the same cell displays helical gliding (4:04 min:s), followed by twirling (4:13 min:s). The ability of an individual cell to switch between gliding modes on the timescale of minutes is consistent with the self-organization hypothesis presented in this work. Different self-organized actin states may arise at different points in time, likely in response to altered regulation of actin dynamics, even though the underlying cell structure (for example, IMC and microtubules) remains unchanged.

Supplementary Video 2 Speckle imaging reports on dynamics of myosin in extracellular *Toxoplasma gondii*. Example of TIRF imaging of MLC1-halo single-molecule dynamics (cyan) in an extracellular parasite, with bulk labelling (magenta) to show cell position. MLC1-halo was expressed at low levels, labelled with 10 pM of Janelia Fluor 549 to visualize single molecules (cyan) and labelled with 200 pM of Janelia Fluor 647 to visualize the cell (magenta). MLC1 molecules frequently remained immobile or bound, on the timescale of seconds. Five molecules, indicated by arrowheads, remain bound for the length of the video. Time is in min:s.

Supplementary Video 3 Speckle imaging reports on dynamics of actin in extracellular *Toxoplasma gondii*. Example of TIRF imaging of *Toxoplasma* actin (halo-actin) single-molecule dynamics (cyan) in an extracellular parasite, with bulk labelling (magenta) to show cell position. Halo-actin was expressed at low levels, labelled with 10 pM of Janelia Fluor 549 to visualize single molecules (cyan) and labelled with 500 pM of Janelia Fluor 647 to visualize the cell (magenta). The same video repeats three times to highlight different molecule behaviours. First, ‘b’ labels an immobile (‘bound’) molecule, which persists for a second before disappearing, likely because it unbinds and moves out of the TIRF field. Second, ‘d’ labels a molecule that displays meandering (diffusive) behaviour. Third, ‘m’ labels a directional (‘motor-transported’) molecule that moves persistently towards the anterior of the cell. Time is in min:s.

Supplementary Video 4 Actin flocking model predicts self-organized recirculation (‘cyclosis’) of actin patches in the absence of filament turnover. The simulation begins with a disordered network, and then filament density *ρ* and velocity *v* evolve over time on the *Toxoplasma gondii* tachyzoite cell surface according to the actin self-organization Toner–Tu equations presented in Supplementary Information Sections 6–9. Filaments are stable and conserved, as in Supplementary Fig. 3a,b. Equations were solved and results simulated using the finite element method in COMSOL Multiphysics. The cell surface shape was obtained by soft X-ray tomography.

Supplementary Video 5 Imaging of jasplakinolide-stabilized actin bundles that recirculate up and down the *Toxoplasma gondii* cell. Example of highly inclined and laminated optical sheet (‘dirty TIRF’) imaging of halo-actin in extracellular *Toxoplasma* tachyzoites treated briefly with 1 μM jasplakinolide to stabilize actin filaments. The recirculating bundle sometimes moves out of the ‘dirty TIRF’ field but is clearly visible when parallel to the imaging plane (for example, 0:13–0:15 min:s).

Supplementary Video 6 Actin flocking model predicts the emergence of self-organized unidirectional flow in the presence of filament turnover. The simulation begins with a disordered network, and then filament density *ρ* and velocity *v* evolve over time on the *Toxoplasma gondii* tachyzoite cell surface according to the actin self-organization Toner–Tu equations presented in Supplementary Information Sections 6–9. Filaments are polymerized in the conoid (anterior end) at rate *c* = 1,500 μm^−2^ s^−1^ and depolymerized throughout the cell surface at rate *γρ* = (0.5 s^−1^) (*ρ* μm^−2^), as in Supplementary Fig. 3c,d and Supplementary Information Section 7.2. Equations were solved and results simulated using the finite element method in COMSOL Multiphysics. The cell surface shape was obtained by soft X-ray tomography.

Supplementary Data 1 Source data for Supplementary Fig. 8: tuning actin turnover changes the frequency of gliding modes.

## Data Availability

*Toxoplasma gondii* strains, plasmids and imaging data are available upon request. The BioNumbers database referenced in the Supplementary Information is available at https://bionumbers.hms.harvard.edu/search.aspx.

## Code availability

MATLAB and COMSOL code is available at https://github.com/chueschen/Toxoplasma_actin.
