# Supplementary material for: Emergent actin flows explain distinct modes of gliding motility
Source: Nat Phys. 2024 Oct 8;20(12):1989–96. doi: 10.1038/s41567-024-02652-4 (PMC11631758; doi:10.1038/s41567-024-02652-4)
Supplement: Supplementary file 1 — Supplementary Figs. 1–12, Table 1, extended methods, theoretical model, references and appendix. [file 41567_2024_2652_MOESM1_ESM.pdf]

nature physics

Supplementary information https://doi.org/10.1038/s41567-024-02652-4

Emergent actin flows explain distinct modes of gliding motility

In the format provided by the authors and unedited

Contents

1 Supplementary Figures For Main Text **3**
S1. Back-and-forth patch gliding and MLC1 localization. 3
S2. Imaging and tracking myosin (MLC1) and actin. 4
S3. Governing equations of actin filament self-organization. 6
S4. Recirculating 'cyclosis' of F-actin bundles after actin stabilization. 6
S5. The asymmetric *Toxoplasma gondii* cell shape enables consistent chirality and stability of predicted F-actin patterns. 7
S6. Predicted steady-state F-actin density and velocity patterns for different filament turnover rates. 8
S7. Schematic showing how actin patterns dictate gliding movement. 9
S8. Tuning actin turnover changes the frequency of gliding modes. 9

2 Video Legends **12**

3 Supplementary Table 1: Observations of Back-and-Forth Gliding **14**

4 Experimental Methods and Materials **15**
4.1 *Parasite and host cell culture* 15
4.2 *Generation of halo-ACT1 and MLC1-halo strains* 15
4.3 *Single molecule (speckle) and bulk labeling in live parasites* 16
4.4 *Preparation and TIRF imaging of live extracellular parasites* 16
4.5 *Frequency of gliding modes with titrated actin stabilization by jasplakinolide (low concentrations)* 16
4.6 *Jasplakinolide treatment (high concentration) and recirculating actin bundles.* 17
4.7 *MLC1 immunofluorescence and super-resolution confocal microscopy* 17
4.8 *Soft X-ray tomography* 17

5 Image Analysis **18**

6 Theoretical Model of *Toxoplasma gondii* Actin Filament Self-Organization **19**
6.1 Continuum flocking theory: background and model choice 19
6.2 A minimal Toner-Tu flocking theory 20
6.3 Extending flocking theory to capture *Toxoplasma* actin biology: curvature 21
6.4 Extending flocking theory to capture *Toxoplasma* actin biology: filament polymerization and depolymerization 24
6.5 On resistive forces from the environment. 26

7 Parameter Choices and Dimensionless Ratios **26**
7.1 Dimensionless ratios in the theory 26
7.2 Parameter choices for modeling *Toxoplasma* actin self-organization 30

8 Deriving a Tangential Formulation of the Filament Self-Organization Equations for a Curved Surface **32**

9 Numerically Solving the Filament Self-Organization Equations on the *Toxoplasma* Cell Surface **34**
9.1 Spherical harmonic shape analysis of *Toxoplasma* cells and mesh generation 34
9.2 Solving the tangential self-organization equations in COMSOL Multiphysics® 35

10 Appendix **36**
10.1 Dry vs. wet active matter: estimating frictional drag from a fixed surface 36
10.2 Estimates of F-actin polymerization and depolymerization rates 37
10.3 Estimates of resistive forces that could perturb actin 'herding' during *Toxoplasma gondii* gliding 38
10.3.1 Overcoming the drag due to cell motion. 38
10.3.2 Shear forces at the cell-substrate interface. 39
10.3.3 Drag force due to adhesion protein motion through the cell membrane. 40

References **40**

1. Supplementary Figures For Main Text

We begin by presenting, for easy access, the supplementary figures referenced within the body of the main text. Additional figures are embedded through the Supplementary Information document, adjacent to the material that they illustrate or support.

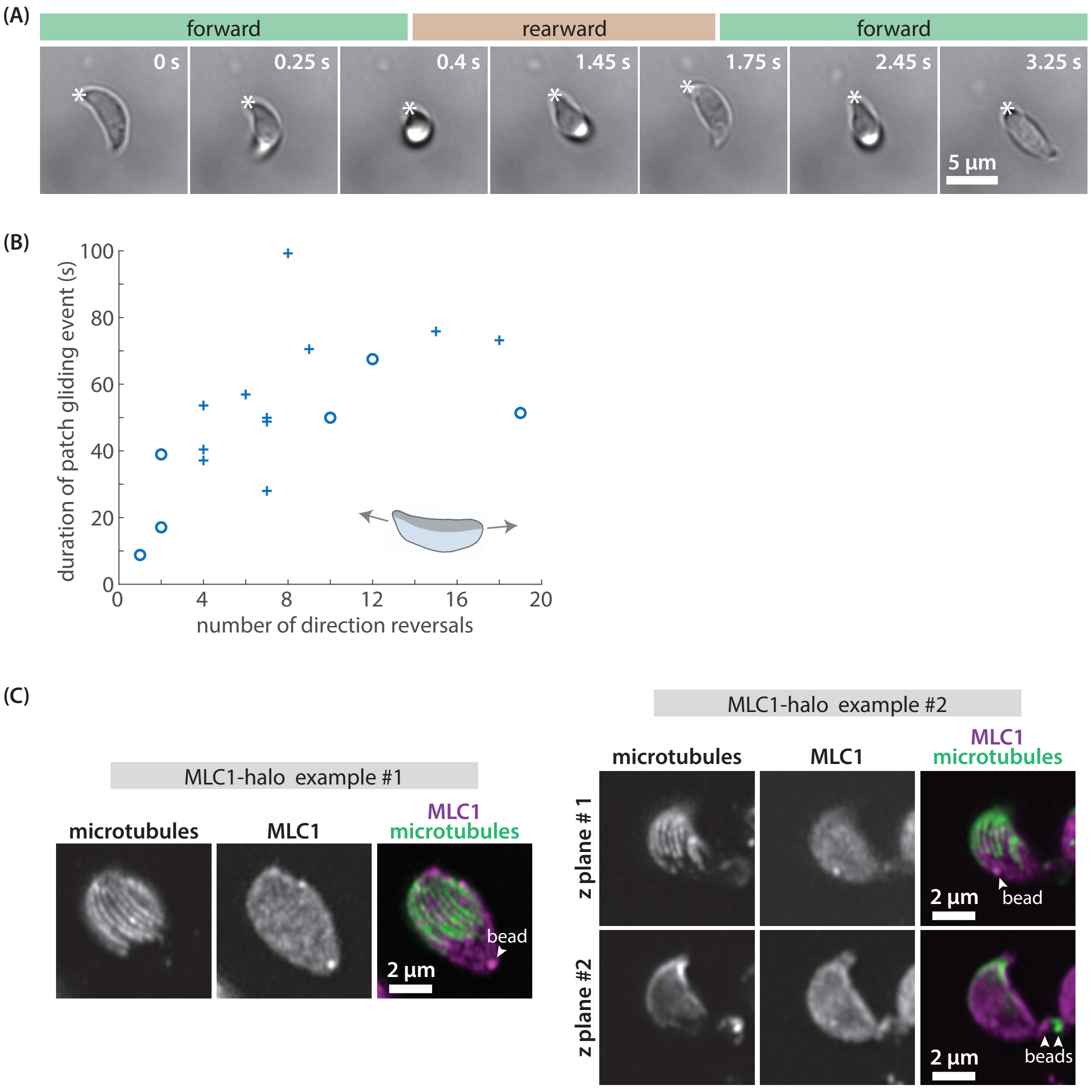

Figure S1. Back-and-forth patch gliding and MLC1 localization. (A) Timelapse showing back-and-forth patch gliding behavior in *T. gondii* tachyzoites (parental strain; see Experimental Methods and Materials). Star marks parasite posterior end. Representative of n = 11 cells analyzed in (B). (B) Quantification of the number of direction reversals and the total back-and-forth gliding event duration from movies of tachyzoites exhibiting patch gliding behavior. If patch gliding events began before the start of a movie recording or extended beyond its end, the numbers reported here represent a lower bound and are indicated by a + marker. We note that an event with one direction reversal corresponds to two body-length glides (forward, reverse), and so on. n = 17 events, 11 cells, 5 experiments. (C) Confocal (Airyscan super-resolution) images of fixed extracellular tachyzoites with retracted conoids (left) and protruded conoids (right) expressing myosin light chain 1 (MLC1)-halo and immunostained for α-tubulin. MLC1, which recruits myosin A to the IMC [1], does not localize exclusively along subpellicular microtubules, in agreement with prior work [2, 3]. Images shown are individual z-slices from examples representative of n = 9 cells. Multicolor beads were used for channel registration.

Figure S1 presents observations inconsistent with a templated model of *Toxoplasma gondii* F-actin transport during gliding, in which F-actin is transported uniformly rearward towards the posterior end of the cell by myosin A. In this model, reviewed in reference [4], a polarized rearward F-actin flow leads to forward traction force and forward (anterior-end-leading) movement of the cell. As highlighted in Figure S1A and B, however, *Toxoplasma gondii* tachyzoites can move both forward and rearward. Further, it has been hypothesized that rearward F-actin transport is directed or templated by the polarized subpellicular microtubules that lie beneath the inner membrane complex (IMC) [5, 4]. However, as shown in Figure S1C and in references [2, 3], the myosin A light chain MLC1 localizes throughout the IMC, not specifically at subpellicular microtubules. Thus, it remains unclear how the polarity of the microtubules could dictate or restrict the polarity of myosin A activity - and direct a rearward-only F-actin flow.

Figure S2 presents additional results relevant to the imaging of single myosin light chain 1 (MLC1) and actin molecules inside living extracellular *Toxoplasma gondii* tachyzoites. In these experiments, fusion proteins expressed at low levels (MLC1-halo and halo-actin, see Experimental Methods and Materials) were labeled sparsely with bright, photostable Janelia Fluor dyes using the HaloTag system. The labeling control shown in Figure S2A shows the specificity of this labeling approach. Figure S2B-D reports speeds from automated detection and tracking of MLC1 and actin molecules using the u-track algorithm [6]. While the tracking settings (see Section 5) required to prevent false linkages between molecules disproportionately reports immobile and slow trajectories, in which detection events can be more confidently linked, automated tracking still showed a larger fraction of mobile actin (Figure S2C-D) compared to myosin (MLC1).To put numbers on the difference between the distribution of actin speeds and the distribution of fixed control and myosin speeds visible by eye in Figure S2D, we can calculate the two-sample Kolmogorov-Smirnov test statistic, which reports on the maximum absolute difference between cumulative distribution functions. It is a nonparametric test that does not assume a specific probability distribution. The Kolmogorov-Smirnov statistic is 0.048 when comparing the speed distributions of immobile fixed-cell control molecules to myosin; 0.15 when comparing the immobile fixed-cell control to actin, and 0.18 when comparing myosin to actin ($p < 0.001$ in all 3 cases, suggesting that each distribution is unique). Importantly, we note that the challenge of using automated methods to capture fast, directional actin movements visible by eye necessitated the use of manual tracking for a focused analysis of directional trajectories shown in Figure 1D-F (see Section 5 for additional discussion).

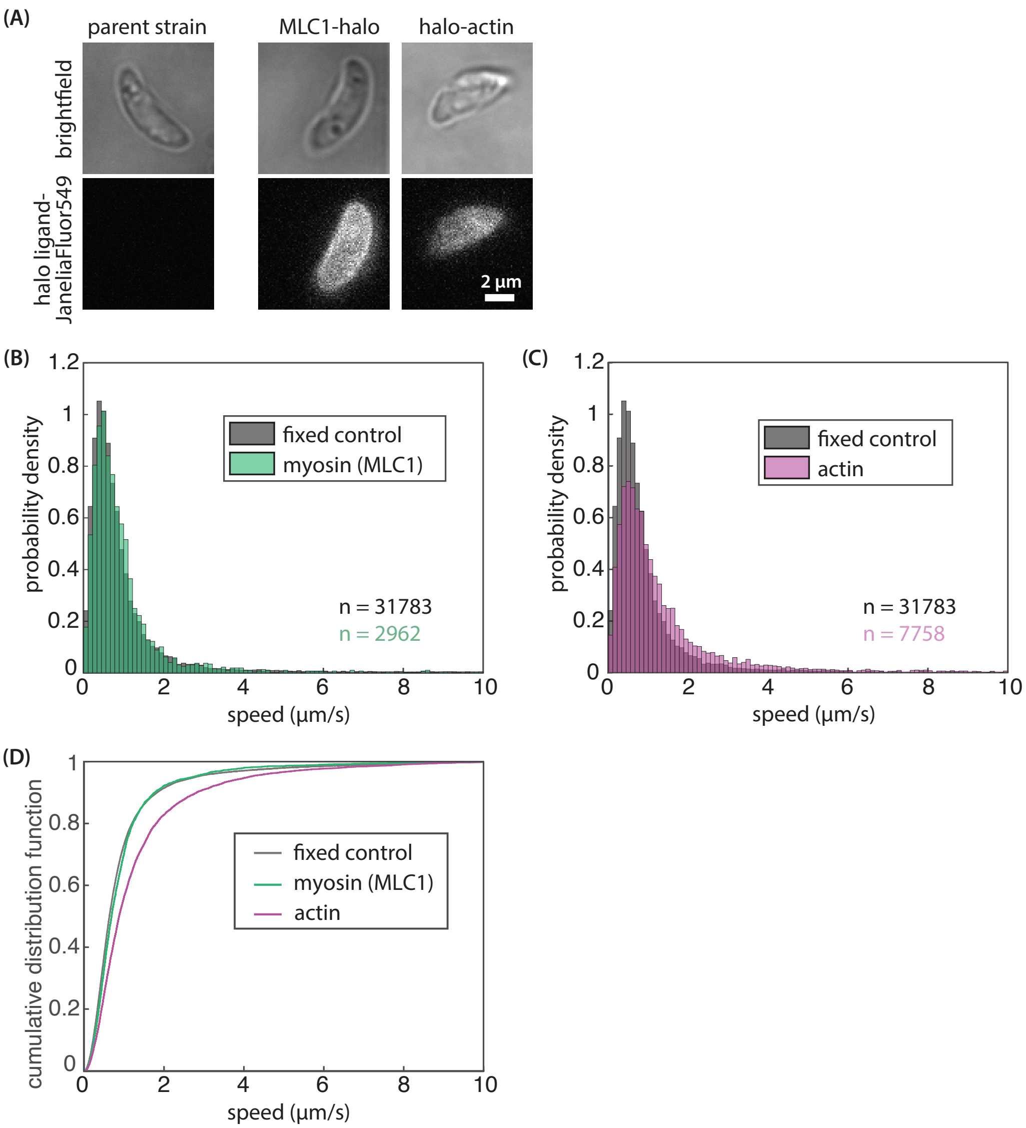

Figure S2. Imaging and tracking myosin (MLC1) and actin. (A) Labeling control showing that even at the highest concentrations (500 pM) of HaloTag Ligand Janelia Fluor used, fluorophores specifically labeled only HaloTag fusion proteins. No fluorescent molecules were detectable in parent strain tachyzoites following labeling and standard washes (see Experimental Methods and Materials). Representative of images of 11 parent strain cells, 8 MLC1-halo cells, 14 halo-actin cells. (B) Histogram of speeds of automatically-tracked single myosin light chain 1 (MLC1) proteins compared to fixed-cell control. (C) Histogram of speeds of automatically-tracked single actin proteins compared to the same fixed-cell control. In the fixed-cell control, halo-actin proteins are labeled and imaged under identical conditions, but cells are fixed before imaging. This condition allows us to establish the baseline speed distribution for static molecules that arises from measurement error when imaging at the rapid temporal resolution required to capture fast dynamics [7]. Given a pixel size of 100 nm and a frame interval of 86 ms, even a one-pixel localization error produces a measured speed of 1.2 µm/s. (D) Cumulative distribution function of fixed cell control, MLC1, and actin speeds. (B-D) Automatic tracking reveals a population of mobile actin molecules, compared to MLC1 and fixed control speed profiles (n = 15 cells for actin, 7 cells for myosin, and 6 cells for the fixed control). We note that the actin mobile population is likely an undercount; automatic tracking disproportionately reports immobile and slow trajectories, in which detection events are easier to confidently link. Fast, directional movements visible by eye were challenging to capture with automated methods, necessitating the use of manual tracking for a focused analysis of directional trajectories (Figure 1; as discussed in Supplemental Information Section 5).

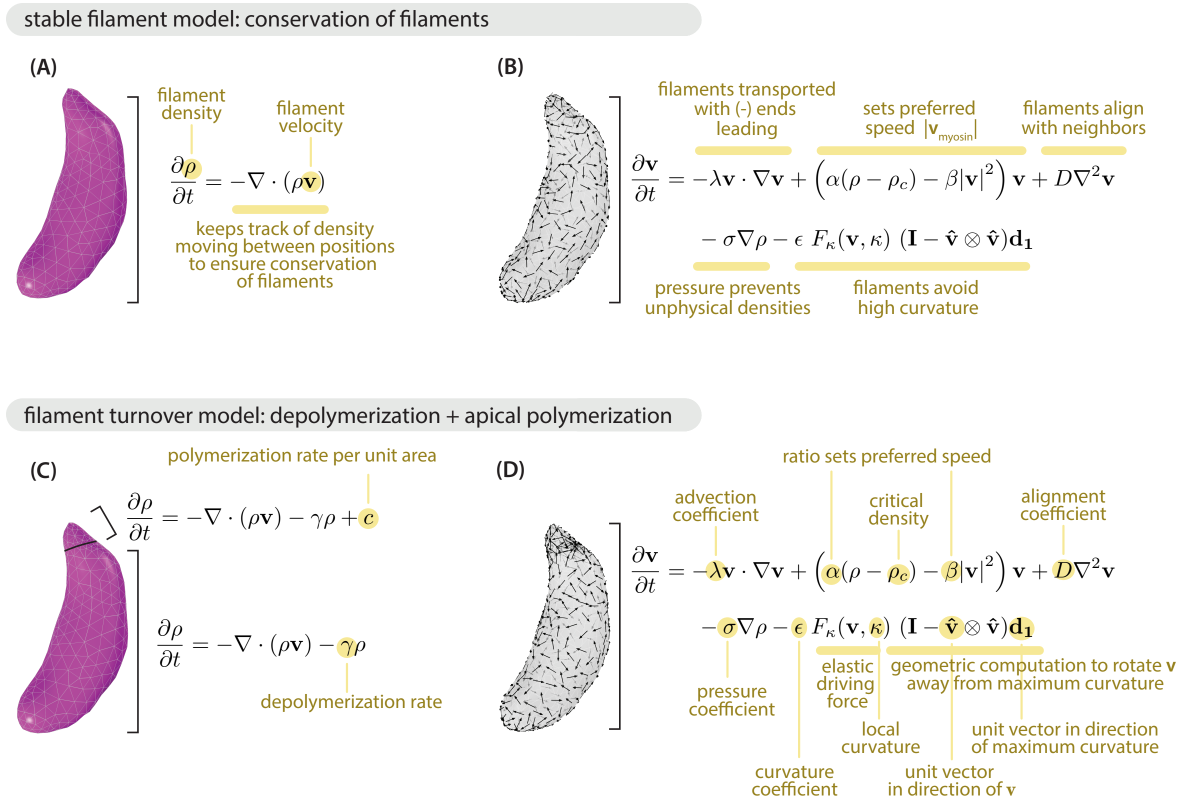

Figure S3. Governing equations of actin filament self-organization. (A) In a regime in which filaments are stable and conserved, the continuity equation governs filament density. (B) Generalized Toner-Tu flocking theory for actin filament self-organization. Different terms correspond to different effects governing filament velocity and speed. In other words, filament velocity evolves according to local rules, implemented mathematically. (C) In a filament turnover regime, incorporating our knowledge of *Toxoplasma* actin biology, we allow filament polymerization at the cell's anterior (apical) end and filament depolymerization throughout. (D) Filament velocity evolves according to the same equation (B) as in the stable filament regime. We include this second version in order to pedagogically annotate the coefficients that tune each term.

In Figure S3, we annotate the Toner-Tu flocking model used to predict emergent actin filament organization and dynamics at the *Toxoplasma gondii* cell surface. For the interested reader, the model is presented in detail in Section 6 and reference [8]. In brief, actin filament organization is described by two fields: the scalar field ρ, which captures filament density, and the velocity vector field $\mathbf{v}$, which captures both filament polarity (orientation of v) and speed (magnitude of v). The continuity equation ensures conservation of stable filaments (Figure S3A) or is modified to capture filament polymerization at the cell anterior end and filament depolymerization throughout (Figure S3C). As shown in Figure S3B and D, filament velocity evolves according to a minimal version of the Toner-Tu flocking model, with an additional term that penalizes filament curvature which is derived in Section 6.3.

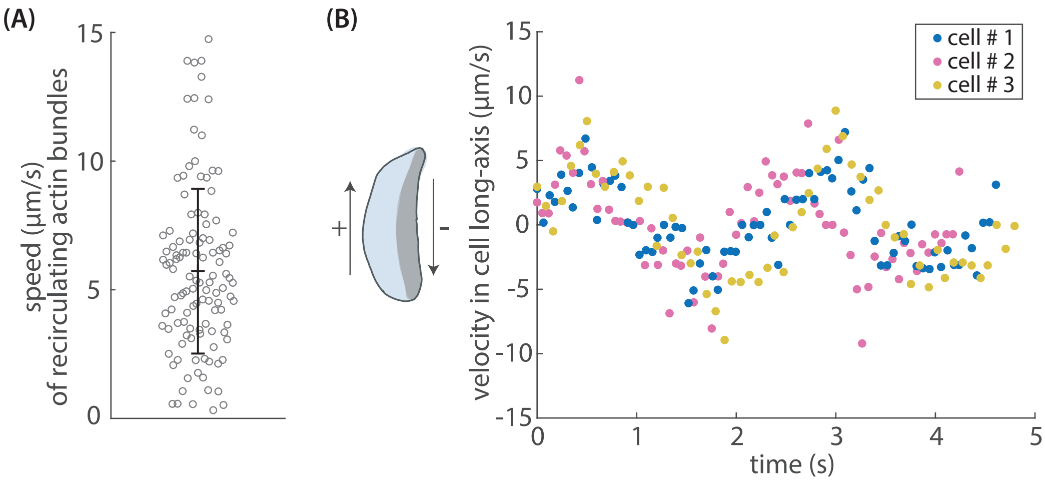

Figure S4. Recirculating 'cyclosis' of F-actin bundles after actin stabilization. After brief (15-30 min) treatment of extracellular *Toxoplasma* tachyzoites with with 1 μM of the actin-stabilizing drug jasplakinolide, we observed a continuous recirculating 'cyclosis' of F-actin bundles, which is shown in Figure 3B of the main text and in Video 4. Here, panel (A) shows measured speeds of these recirculating actin bundles, which have a mean of 5.7 μm/s ± a standard deviation of 3.3 μm/s (black lines; n = 116 from 10 cells). (B) To illustrate how velocity varies as the actin bundles recirculate up and down the *Toxoplasma* cell, we report a one-dimensional actin bundle velocity in the cell long-axis for three example cells, over two full periods of recirculation. For each cell, bundle tracking begins at one end of the cell at t = 0 s. As the actin bundle moves toward the other pole, it has a positive velocity; when it returns, it has a negative velocity.

Figure S4 provides additional quantification of the observation of recirculating actin bundles after brief treatment with high concentrations of the actin-stabilizing drug jasplakinolide, as presented in Fig. 3B of the main text. We particularly enjoy the way Figure S4B illustrates the oscillatory nature of the actin bundle velocity during this recirculating behavior, for three example cells.

In Figure S5, we highlight that we do believe that features of the specific, stereotyped shape of *Toxoplasma gondii* cells are essential for self-organization of the actin flows that drive coherent gliding motility. To demonstrate this point, we have performed a computational comparison of the self-organized actin patterns predicted a *Toxoplasma*-sized ellipsoid and actual *Toxoplasma* cell shapes. As shown in the figure, the fully asymmetric *Toxoplasma* cell shape dictates the chirality of the predicted unidirectional self-organized actin flow, likely because the cell itself has a subtle right-handed chiral twist. By contrast, the actin pattern predicted for the *Toxoplasma*-sized ellipsoid (with the identical model and parameters) stochastically breaks chiral symmetry, becoming left-handed or right-handed depending on the particular disordered (randomly-oriented) initial condition. In addition, the fully asymmetric *Toxoplasma* cell shape stabilizes the predicted unidirectional actin pattern and allows it to reach a true steady-state, as shown in Figure S5B. In the ellipsoid, for which the two short axes have the same dimension (a = b), the predicted actin pattern rotates circumferentially and indefinitely as shown in Figure S5C and D. Notice that the +1 defect or vortex seen towards the posterior end of the ellipsoid moves over time. We believe that both these features (right-handed chirality and a steady-state actin pattern) are important for successfully driving the unidirectional gliding modes (Figure 4D-F).

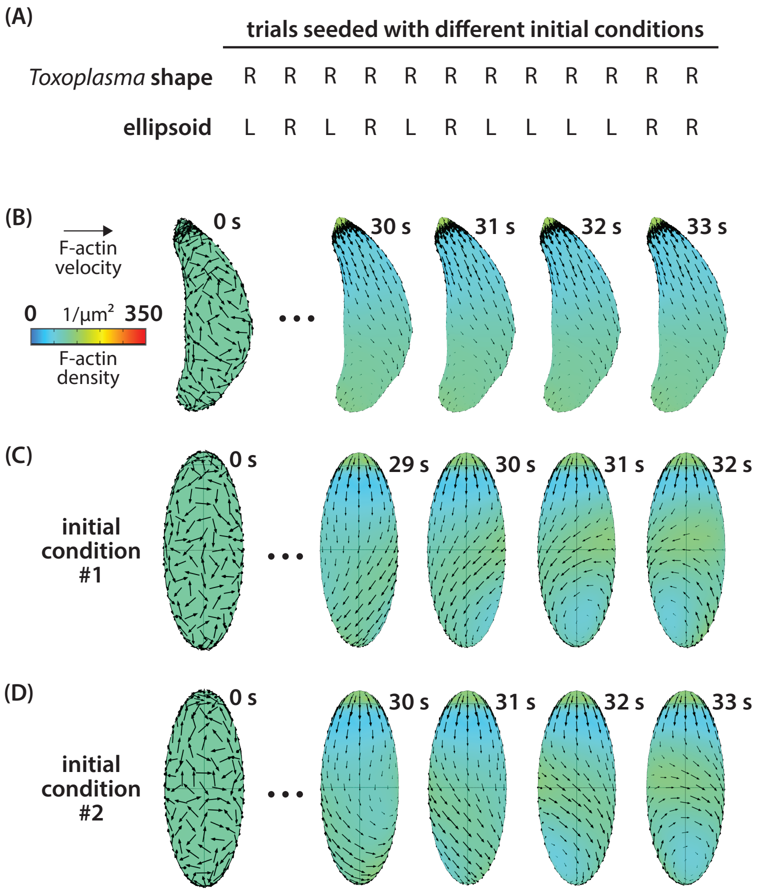

Figure S5. The asymmetric *Toxoplasma gondii* cell shape enables consistent chirality and stability of predicted F-actin patterns. For the actin self-organization model and parameters presented in Equations 2-4 and Figure 4A, multiple computational trials were performed on the fully asymmetric Toxoplasma cell shape or on a Toxoplasma-sized ellipsoid described by $x^2/a^2 + y^2/b^2 + z^2/c^2 = 1$, where a = b = 1.2 μm and c = 3 μm. Each trial used identical parameters but was seeded with a different disordered initial condition. (A) For twelve example trials with different initial conditions, the fully asymmetric Toxoplasma shape led to a steady-state actin velocity pattern with right-handed chirality, while the actin pattern predicted for the Toxoplasma-sized ellipsoid stochastically broke chiral symmetry, becoming left-handed or right-handed. In the convention of the hand rule, the thumb points up toward the cells apical end. (B) The actin pattern predicted on the Toxoplasma cell shape always converged on the same steady-state pattern. (C-D) The actin pattern predicted on the ellipsoid changed handedness depending on initial condition and did not reach a steady-state. Note the circumferential rotation of the posterior vortex.

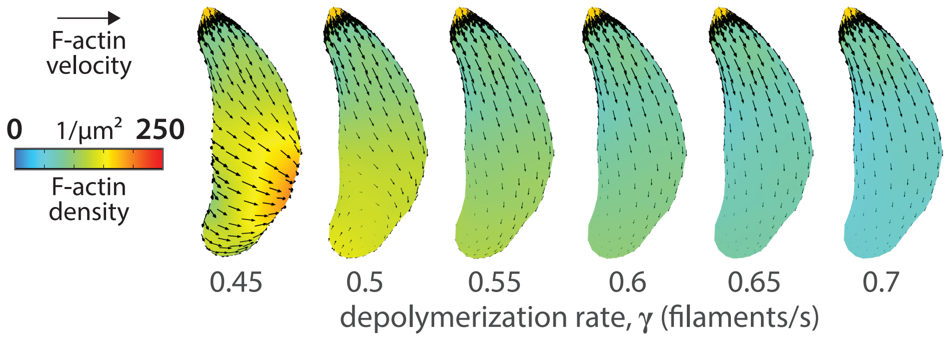

Figure S6. Predicted steady-state F-actin density and velocity patterns for different filament turnover rates. Tuning actin depolymerization rate (γ, filaments/s) in the Toner-Tu actin self-organization model changes features like chirality and density gradient in the emergent steady-state actin patterns predicted. Shown here are steady-state filament density (color) and velocity (black arrows) for a filament polymerization rate $c = 1500$ $\mu m^{-2} s^{-1}$ (estimated in Section 7.2) and filament turnover rates γ as indicated.

In Figure S6, we highlight the effect of actin filament depolymerization rate on predicted steady-state unidirectional F-actin flows. These flow patterns, which we call "steady-state" because the density and velocity fields reach a stable solution which they hold over time, consist of largely unidirectional, rearward actin flow and are labeled as "unidirectional" (dark purple circles) in Figure 4B. Those steady states that lie in a regime close to the transition to recirculation are most consistent with cell motions during helical gliding, circular gliding, and twirling, as depicted in Figure 4D-F. In the example shown here, this regime corresponds to depolymerization rates of $\gamma = 0.45$-0.55 filaments/s. (Lower rates of depolymerization lead to F-actin recirculation ("cyclosis," orange circles), as shown in Figure 4B.) Our theoretical model indicates that in *Toxoplasma gondii* parasites whose actin dynamics lie in this regime, actin self-organization alone is sufficient to generate patterns that explain helical gliding, circular gliding, and twirling. Interesting, this finding is consistent with experimental results suggesting that careful tuning of actin filament stability is critical for normal gliding behaviors [9, 10, 11, 12].

We note that this sufficiency does not preclude the existence of additional complexity not explored here, like contributions from inner membrane complex (IMC) shape or IMC surface filaments [13].

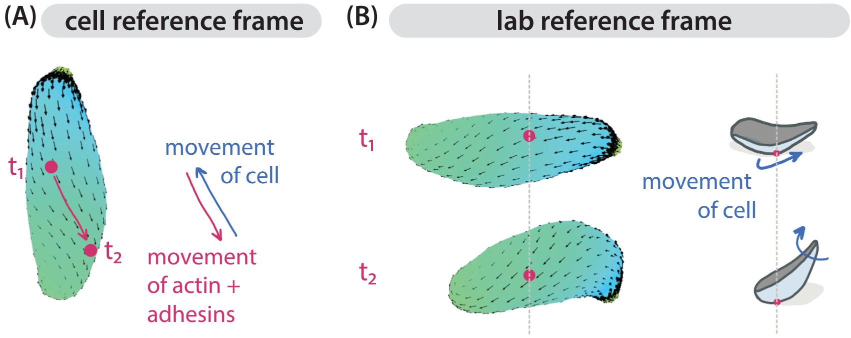

Figure S7. Schematic showing how actin patterns dictate gliding movement. In this schematic, we view from below a cell whose 'back' is in contact with the substrate. (A) In the reference frame of the cell, actin and adhesins flow down and across the cell (pink arrow) from time t_1 to t_2. (B) In the reference frame of the lab, actin, adhesins, and the point of substrate contact (pink dots) stay stationary from time t_1 (top row) to t_2 (bottom row). The cell moves over the contact point, translating and rotating (blue arrows). As illustrated here, local F-actin polarity and velocity dictates the direction of myosin-powered traction force and the direction of cell movement.

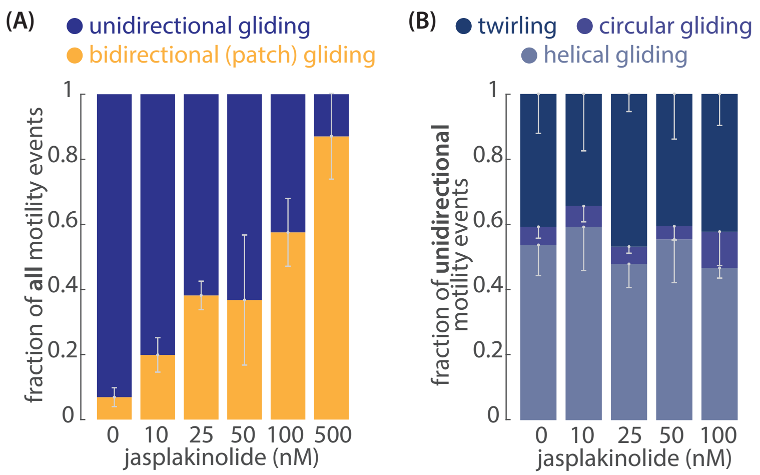

Figure S8. Tuning actin turnover changes the frequency of gliding modes. The actin-stabilizing drug jasplakinolide was titrated to tune F-actin turnover. (A) As jasplakinolide concentration increased, decreasing actin turnover, more frequent bidirectional (patch) gliding and less unidirectional gliding was observed. (B) Titrating jasplakinolide concentrations to tune F-actin turnover did not change the fractional breakdown of unidirectional gliding events into twirling(top), circular (middle), and helical (bottom) movements. Note that this analysis includes only the unidirectional (purple) events from part A. Both plots show weighted means and standard deviations (light gray bars) from 3 independent experiments with a total of n = 116 (0 nM), 156 (10 nM), 152 (25 nM), 117 (50 nM), 106 (100 nM), and 54 (500 nM) total gliding events and n = 108 (0 nM), 125 (10 nM), 94 (25 nM), 74 (50 nM), and 45 (100 nM) unidirectional gliding events. The 500 nM jasplakinolide condition was excluded from the analysis in part B because so few unidirectional events in total (n = 7) were observed.

In Figure S8, we show a first step towards experimental tests of the theoretical findings and working model presented in Figure 4. That working model predicts that decreasing F-actin turnover - by increasing filament polymerization or decreasing filament depolymerization - will shift actin self-organization towards a recirculating state (cyclosis; orange part of Figure 4B phase space) and drive more bidirectional (patch) gliding. It also predicts (Figure 4C-F) that helical gliding, circular gliding, and twirling are all driven by unidirectional F-actin flows at higher rates of F-actin turnover, and that whether a given unidirectional actin flow pattern drives helical vs. circular vs. twirling movements depends on substrate contact point position, not on differences in F-actin turnover.

Here, inspired by classic observations that high concentrations (2 µM) of the actin-stabilizing drug jasplakinolide lead to back-and-forth cell movements [9], we tuned F-actin turnover in live extracellular *Toxoplasma gondii* tachyzoites by treating them with a panel of lower jasplakinolide concentrations. We then imaged and analyzed their gliding behaviors. Figure S8 shows how common a given gliding mode is (as a fraction of total gliding events) for different jasplakinolide concentrations. Bar heights show weighted mean; light gray bars show weighted standard deviation.

Figure S8A shows that the frequency of bidirectional (patch) gliding does indeed increase with increasing F-actin stabilization (decreased F-actin turnover). In Figure S8B, we analyze only the unidirectional motility events shown in purple in part A. Consistent with the working model presented in Figure 4 and discussed above, tuning actin turnover with jasplakinolide does not significantly change the proportion of helical gliding, circular gliding, and twirling events.

For the fraction of cells in each motility mode reported in Figure S8, means were calculated by 'pooling' all measurements of motility events from 3 independent experiments. This is equivalent to a weighted mean, $x^* = \sum w_i x_i / \sum w_i$, where for experiment i the weight w_i is the total number of motility events measured and x_i is the fraction of cells in a given mode. Weighted standard deviations are calculated as $\sum w_i \left(x_i - x^*\right)^2 \ / \ \left(\sum w_i - \frac{\sum w_i^2}{\sum w_i}\right)$, where the denominator is a correction to yield an unbiased estimator

given 'reliability' weights [14].

To provide a sense of the statistical significance of these changes in gliding behavior as a function of jasplakinolide concentration, we performed one-way analysis of variance (ANOVA) to test the hypothesis that the means (*e.g.*, mean fraction undergoing bidirectional patch gliding) are the same across jasplakinolide concentrations, assuming normality. (We note that a formal test of the assumption of normality was limited by the nature of the data: the fraction of cells in a given mode, from three experiments. For completeness, we did perform a one-sample Kolmogorov-Smirnov test of normality for all conditions shown in Figure S8. This one-sample KS test could not reject the null hypothesis of a normal distribution for all gliding modes (patch, helical, circular, twirling) for 0-100 nM jasplakinolide; for the fraction of patch gliding cells at 500 nM, the p-value was 0.1.)

With those assumptions in mind, we performed our one-way ANOVA to get a sense of the significance of differences across conditions. First, we tested the hypothesis that the mean fraction of cells undergoing bidirectional patch gliding is the same across jasplakinolide concentrations. We calculated an F ratio of 20.7 and p-value of 1.6×10^{-5}, indicating this is not the case. Below, we show the ANOVA p-values for each pairwise comparison for bidirectional patch gliding (Figure S8A). Second, for the fractional composition of unidirectional gliding events (helical vs. circular vs. twirling, from 0 nM to 100 nM jasplakinolide, Figure S8B), one-way ANOVA F-ratios were 0.50, 0.57, and 0.16, respectively, and p-values were 0.74, 0.69, and 0.95, respectively. All p-values for all unidirectional gliding mode pairwise comparisons were larger than 0.65, so we do not include detailed pairwise tables below.

	**0 nM**	**10 nM**	**25 nM**	**50 nM**	**100 nM**	**500 nM**
**0 nM**	-	0.494	0.040	0.020	0.002	8.46E-06
**10 nM**	0.494	-	0.565	0.346	0.037	7.65E-05
**25 nM**	0.040	0.565	-	0.998	0.469	0.001
**50 nM**	0.020	0.346	0.998	-	0.707	0.002
**100 nM**	0.002	0.037	0.469	0.707	-	0.017
**500 nM**	8.46E-06	7.65E-05	0.001	0.002	0.017	-

p-values from ANOVA on frequency of **bidirectional** gliding events, comparing different jasplakinolide concentrations

We also note that we look forward to future development of tools to more precisely tune the key 'dials' of F-actin polymerization rate and F-actin depolymerization rate explored theoretically in Figure 4B, allowing a quantitative dialogue between theory and experiment.

2. Video Legends

Video 1. Diverse gliding modes: an individual cell performs back-and-forth gliding, helical gliding, and then twirling. In addition to recommending the classic reference [15], we include this video to orient readers unfamiliar with gliding motility and to illustrate that an individual cell can switch between gliding modes. In this brightfield microscopy video, an extracellular *Toxoplasma gondii* tachyzoite glides back and forth (0:00 min:s), displaying so-called patch or pendulum gliding (see Table S1). Several minutes later, the same cell displays helical gliding (4:04 min:s), followed by twirling (4:13 min:s). The ability of an individual cell to switch between gliding modes on the timescale of minutes is consistent with the self-organization hypothesis presented in this work. Different self-organized actin states may arise at different points in time, likely in response to altered regulation of actin dynamics, even though the underlying cell structure (e.g., IMC and microtubules) remains unchanged.

Video 2. Single-molecule imaging reports on dynamics of myosin in extracellular *Toxoplasma gondii.* Example of total internal reflection fluorescence (TIRF) imaging of myosin light chain 1 (MLC1-halo) single molecule dynamics (cyan) in an extracellular parasite, with bulk labeling (magenta) to show cell position. MLC1-halo was expressed at low levels, labeled with picomolar concentrations of Janelia Fluor 549 to visualize single molecules (cyan), and labeled with nanomolar concentrations of Janelia Fluor 647 to visualize the cell (magenta). MLC1 molecules frequently remained immobile, or bound, on the time scale of seconds. Five molecules, indicated by arrowheads, remain bound for the length of the movie. Time is in min:s.

Video 3. Single-molecule imaging reports on dynamics of actin in extracellular *Toxoplasma gondii.* Example of total internal reflection fluorescence (TIRF) imaging of *Toxoplasma* actin (halo-actin) single molecule dynamics (cyan) in an extracellular parasite, with bulk labeling (magenta) to show cell position. Halo-actin was expressed at low levels, labeled with picomolar concentrations of Janelia Fluor 549 to visualize single molecules (cyan), and labeled with nanomolar concentrations of Janelia Fluor 647 to visualize the cell (magenta). The same video repeats three times in order to highlight different molecule behaviors. First, “b” labels an immobile (“bound”) molecule, which persists for a second before disappearing - likely because it unbinds and moves out of the TIRF field. Second, “d” labels a molecule that displays meandering (diffusive) behavior. Third, “m” labels a directional (“motor-transported”) molecule that moves persistently towards the anterior of the cell. Time is in min:s.

Video 4. Actin flocking model predicts self-organized recirculation (“cyclosis”) of actin patches in the absence of filament turnover. The simulation begins with a disordered network, and then filament density ρ and velocity $\mathbf{v}$ evolve over time on the *Toxoplasma gondii* tachyzoite cell surface according the actin self-organization Toner-Tu equations presented in Sections 6 - 9. Filaments are stable and conserved, as in Supplementary Figure S3 A-B. Equations were solved and results simulated using the finite element method in COMSOL Multiphysics®. The cell surface shape was obtained by soft X-ray tomography.

Video 5. Imaging of jasplakinolide-stabilized actin bundles that recirculate up and down the *Toxoplasma gondii* cell. Example of HILO (‘dirty TIRF’) imaging of halo-actin in extracellular *Toxoplasma* tachyzoites treated briefly with 1 μM jasplakinolide to stabilize actin filaments. The recirculating bundle sometimes moves out of the ‘dirty TIRF’ field but is clearly visible when parallel to the imaging plane (e.g., 0:13-0:15 min:s).

Video 6. Actin flocking model predicts the emergence of self-organized unidirectional flow in the presence of filament turnover. The simulation begins with a disordered network, and then filament density ρ and velocity $\mathbf{v}$ evolve over time on the *Toxoplasma gondii* tachyzoite cell surface according the actin self-organization Toner-Tu equations presented in Sections 6-9. Filaments are polymerized in the conoid (anterior end) at rate $c = 1500\ \mu\text{m}^{-2}\text{s}^{-1}$ and depolymerized throughout the cell surface at rate $\gamma\rho = (0.5\ \text{s}^{-1})\ (\rho\ \mu\text{m}^{-2})$, as in Supplementary Figure S3C-D and Section 7.2. Equations

were solved and results simulated using the finite element method in COMSOL Multiphysics®. The cell surface shape was obtained by soft X-ray tomography.

3. Supplementary Table 1: Observations of Back-and-Forth Gliding

**Organism & life stage**	**Condition**	**Expected effect on actin**	**Observed bidirectional gliding phenotype**	**Reference**
T. gondii tachyzoites	"Wild-type" extracellular RH in media conditioned by human foreskin fibroblasts	N/A	"Patch" gliding with frequent pauses: e.g., gliding forward, rearward, forward, flipping over the posterior cell end, rearward, pausing, etc. Cell-substrate contact point is stationary in the lab reference frame.	This study (Fig. S1, Video 1)
T. gondii tachyzoites	Jasplakinolide treatment	Increased filament stability	"frequent reversal of direction often resulted in a behavior called "rolling," during which the parasite moved forward, raising its anterior end off the substrate, then reversed course, elevating the posterior end."	Wetzel et al., 2003 [9]
T. gondii tachyzoites	Small molecule treatment	Unknown targets	"parasites... move quickly with frequent changes in direction"	Carey et al., 2004 [16]
T. gondii tachyzoites	"ADF cKO" (actin depolymerizing factor conditional knockout)	Increased filament stability	"frequent reversals of direction leading to no net movement... back-and-forth rocking motions"	Mehta & Sibley, 2011 [10]
T. gondii tachyzoites	"DOC2.1 mutation"	Blocked microneme secretion	"shuffling, a distinct motility mode in which parasites abruptly move back and forward"	Farrell, Thirugnanam et al., 2012 [17]
P. berghei sporozoites	"coronin(-)"	Loss of filament crosslinking; possibly increased filament stability due to decreased ADF recruitment	"often detached from the substrate, frequently moving just back and forth over a single adhesion site... In addition... bending and flexing movements without moving forward"	Bane et al., 2016 [18]
P. berghei sporozoites (from hemolymph)	"Wild-type" sporozoites	N/A	"Patch" gliding: "sporozoites continuously move over a single spot in a back-and-forth manner at similar speeds in both directions"	Münter et al., 2009 [19]

Organism & life stage	Condition	Expected effect on actin	Observed bidirectional gliding phenotype	Reference
P. berghei sporozoites (from hemolymph)	"trap(-)" (truncated adhesin protein TRAP [20]) isolated from mosquito hemolymph	Unknown; truncated TRAP may fail to bind an adaptor (GAC homolog?) that regulates F-actin stability[21]	"Patch" gliding: "sporozoites continuously move over a single spot in a back-and-forth manner at similar speeds in both directions"	Münter et al., 2009 [19]
P. berghei sporozoites (from midgut)	Mutations in C-terminal cytoplasmic tail of TRAP	Unknown; mutants fail to bind an adaptor (GAC homolog?) that regulates F-actin stability [21]	"Pendulum" gliding: "repeated cycles of (a) gliding over one third of a circle, (b) stopping for usually 1-2 s, and (c) moving back to the original position"	Kappe et al., 1999 [22]
P. berghei sporozoites (from salivary gland)	Mutations in actin subdomain 4	Reduced actin filament turnover	"parasites... frequently paused and reversed direction during migration"	Douglas et al., 2018 [11]
P. berghei sporozoites (from midgut, hemolymph)	"cbβ (-)": capping protein subunit b loss of function	Unrestricted filament polymerization	"cpβ(-) sporozoites display only non-productive motility patterns, such as bending, flexing and pendulum movement"	Ganter et al., 2009 [12]

4. Experimental Methods and Materials

4.1. Parasite and host cell culture

Toxoplasma gondii tachyzoites were maintained by serial passage in primary human foreskin fibroblasts (HFFs) in Dulbeccos modified Eagles high glucose medium (DMEM; Gibco 11960-044) with 10% heat-inactivated fetal bovine serum (FBS; Corning 35-011-CV), 2 mM glutamine (Sigma-Aldrich G7513), 100 U/ml penicillin, and 100 µg/ml streptomycin (Gibco 15140122) at 37°C in 5% CO_2. In brief, to passage parasites, infected HFF monolayers were suspended in media by scraping, syringe lysed using a 25-gauge blunt-end needle (SAI Technologies B25-50), and added at a 150-fold dilution to an confluent uninfected HFF monolayer, every 2-4 days. HFFs were obtained from the neonatal clinic at Stanford University following routine circumcisions that are performed at the request of the parents for cultural, health, or other personal medical reasons (i.e., not in any way related to research). These foreskins, which would otherwise be discarded, were fully deidentified and therefore do not constitute human subjects research. Uninfected HFFs were maintained in the supplemented DMEM described above, passaged using 0.25% trypsin-EDTA (Gibco 25200056), and discarded after passage 15.

4.2. Generation of halo-ACT1 and MLC1-halo strains

In brief, halo-TgACT1 or TgMLC1-halo fusions under the control of a weak promoter (from TGGT1_239010, gift of M. Panas [23]) were incorporated into the genome of the *Toxoplasma gondii* type I RH Δhxgprt

Δku80 strain [24]. In detail, p239010-halo-C1-HXGPRT and p239010-halo-N1-HXGPRT vectors were created by replacing a region of the pGRA-3xHA-HPT vector [25] (from the pGRA promoter through the translated region) with: (1) the TGGT1_239010 promoter and 5' UTR to drive low expression levels; (2) the HaloTag sequence (Promega); and (3) a serine-glycine linker and multiple cloning site either C-terminal ("C1") or N-terminal ("N1") to the HaloTag sequence. TgACT1 (TGGT1_209030) or TgMLC1 (TGGT1_257680) were then synthesized and cloned into the p239010-halo-C1-HXGPRT or p239010-halo-N1-HXGPRT vector, respectively (Epoch Life Science, Inc., Missouri City, TX). For transfection with p239010-halo-ACT1-HXGPRT or p239010-MLC1-halo-HXGPRT, RH Δhxgprt Δku80 parasites were mechanically released in PBS, pelleted, and resuspended in 20 μl P3 primary cell Nucleofector solution (Lonza) with 15 μg DNA, and electroporated using the Amaxa 4D Nucleofector (Lonza). Transfected parasites were permitted to infect and grow in confluent HFFs for 48 h, after which time the media was supplemented with 50 μg/ml mycophenolic acid and 50 μg/ml xanthine for HXGPRT selection. Parasites were passaged 4 times over 10-12 days in selection media before being singly cloned into 96-well plates by limiting dilution. Clones were expanded and screened for HaloTag expression by incubating intracellular parasites overnight with 50 nM TMR HaloTag Ligand (Promega G8251), washing 5x with PBS to remove unbound dye, fixing with 4% paraformaldehyde (EMS AA433689M) for 15 min, and imaging fluorescence.

4.3. Single molecule (speckle) and bulk labeling in live parasites

Before infected HFFs were lysed to release parasites for imaging, parasites within infected HFF monolayers were labeled for 3 h at 37°C with Janelia Fluor 549 HaloTag Ligand (Promega GA1110) and Janelia Fluor 646 HaloTag Ligand (Promega GA1120) [26] at a concentration of 1-10 pM for single molecule imaging or 100-500 pM for bulk population imaging. For example, the MLC1-halo parasites shown in Fig. 1 show MLC1 labeled with 1-10 pM (cyan speckles) and at 100-500 pM (magenta bulk population of MLC1, showing the shape of the cell). Because Janelia Fluor dyes bleach over time in storage, dye concentration must be optimized empirically and adjusted on the timescale of months; furthermore, care should be taken to avoid more than two or three freeze-thaw cycles before use. When subpellicular microtubule imaging was used to determine parasite polarity, infected HFFs were labeled with 100 nM siR-tubulin and 10 μM verapamil (Cytoskeleton, Inc. CY-SC002) alongside 1-10 pM Janelia Fluor 549. Before parasite release, infected HFF monolayers were washed 7x with DMEM to ensure removal of unbound dye.

4.4. Preparation and TIRF imaging of live extracellular parasites

To release parasites, infected HFFs were scraped and syringe lysed in fresh phenol red-free DMEM with a 27-gauge needle (SAI Technologies B27-50). Freshly released parasites were placed on 35 mm #1.5 glass-bottomed dishes (Cellvis D35-20-1.5-N; incubated with 10% FBS before use) with a confluent monolayer of human foreskin fibroblasts (HFFs) grown on Snapwell Insert polyester membranes (Corning Costar CLS3801) suspended approximately 0.2 mm above them. Parasites were imaged at 30°C using objective-type total internal reflection fluorescence (TIRF) microscopy on an inverted microscope (Nikon TiE) with a heated Apo TIRF 100 oil objective of numerical aperture 1.49 (Nikon) and controlled using Micromanager 1.4 [27]. To enable simultaneous two-color imaging, samples were excited with both 532 nm (Crystalaser) and 635 nm (Blue Sky Research) lasers, and emitted light passed through a quad-edge laser-flat dichroic with center/bandwidths of 405 nm/60 nm, 488 nm/100 nm, 532 nm/100 nm, and 635 nm/100 nm from Semrock (Di01-R405/488/532/635-25x36) and corresponding quad-pass filter with center/bandwidths of 446 nm/30 nm, 510 nm/30 nm, 581 nm/30 nm, 703 nm/30 nm band-pass filter (FF01- 446/510/581/703-25). Emission channels were then separated as previously described [28] and recorded on an electron-multiplying charge-coupled device (EMCCD) camera (Andor iXon).

4.5. Frequency of gliding modes with titrated actin stabilization by jasplakinolide (low concentrations)

Live extracellular parasites were prepared as in subsection 4.4 and added to microgrids of 75 μm x 75 μm square PDMS wells (Microsurfaces MGA-075-02) in glass-bottomed 24-well plates. Upon addition

of 650,000 parasites per well, plates were spun at 100 g for 3 min to settled parasites into grids. For jasplakinolide experiments, the indicated concentrations (Figure S8) of jasplakinolide (Millipore Sigma J4580) were then added and mixed by pipetting. Imaging began 40-50 min later. Multiple stage positions were imaged per condition, and microgrid walls prevented shear stress or parasite detachment from sloshing of imaging media. Brightfield images were acquired at a frame rate of 5 frames per second at 37°C in 5% CO_2 using a Nikon Ti-E inverted microscope with a 20X/0.5 NA Plan Fluor CFI air objective and an Andor Neo camera. Image acquisition was controlled using Micro-Manager software [27].

4.6. Jasplakinolide treatment (high concentration) and recirculating actin bundles.

Live extracellular parasites were prepared as in subsection 4.4, with the addition of 1 μM jasplakinolide (Millipore Sigma J4580) immediately before imaging. In a narrow window of time from approximately 15-30 min after jasplakinolide addition, protruding bundles of actin filaments were observed circling around the periphery of parasites (Figure S4). We note that this recirculating behavior was very sensitive to treatment time and drug concentration. Over time, most protrusions lost this recirculating behavior and became fixed in position at the anterior (apical) end, as previously observed [29]. We speculate that this transition to fixed apical actin bundles occurs as bundles grow long enough (with polymerization favored both by jasplakinolide and by apically-localized FRM1 [21]) to protrude through the conoid and into the cytoplasm and can no longer re-orient to contact myosin motors on the outside of the inner membrane complex. Under ideal treatment conditions, mostl extracellular parasites observed displayed recirculating actin protrusions; under less ideal conditions (e.g., after more than 30 min treatment, or with poorly attached parasites), less than 10% of parasites displayed recirculating protrusions. We also note that to image these large recirculating bundles, we relaxed the steep angle of the excitation light and performed highly inclined and laminated optical sheet (HILO) or 'dirty TIRF' imaging. Thus, fluorescently-labeled actin structures within the cell cytoplasm are visible in addition to the gliding-associated surface actin.

4.7. MLC1 immunofluorescence and super-resolution confocal microscopy

Parasites were released from infected HFF monolayers by scraping and syringe lysis in DMEM with a 27-gauge needle (SAI Technologies B27-50), passed through a 5 μm filter (Millipore Sigma SLSV025LS) and allowed to settle onto #1.5 coverslips at 37°C in 5% CO_2 for 30 min in DMEM + 1 μM calcium ionophore A23187 (Sigma C7522). Subsequent staining steps were performed at room temperature: parasites were fixed with warm 4% paraformaldehyde (EMS AA433689M) for 15 min, washed 3x with phosphate-buffered saline (PBS), incubated with permeabilization-and-blocking buffer (0.1% Triton-X-100 and 2% bovine serum albumin (BSA) in PBS) for 20 min, incubated with mouse anti--tubulin monoclonal antibody DM1α (Sigma T6199; diluted 1:500) and 2 nM Janelia Fluor 646 HaloTag Ligand (Promega GA1120) in permeabilization-and-blocking buffer for 1 h, washed 3x with PBS, incubated with anti-mouse IgG secondary antibody conjugated to Alexa Fluor 488 (Cell Signaling 4408S) for 20 min, washed 3x with PBS, and mounted in ProLong Gold Antifade (ThermoFisher P36934). Samples were imaged using an inverted Zeiss LSM 780 confocal microscope with a 63X/1.4 NA oil objective, 488 nm Ar laser, 633 nm HeNe laser, and a Zeiss Airyscan detector (32-channel gallium arsenide phosphide photomultiplier tube (GaAsP-PMT) area detector), in which using each detector element as an individual pinhole combined with linear deconvolution achieves a spatial resolution below the diffraction limit [30]. All images were acquired using Zen 2.3 (black edition) software (Carl Zeiss).

4.8. Soft X-ray tomography

HFF monolayers, 18-20 hours after parasite infection, were washed two times with Hanks balanced salt solution (HBSS; Gibco 14175095) supplemented with 1 mM magnesium chloride, 1 mM calcium chloride, 10 mM sodium hydrogen carbonate and 20 mM HEPES, pH 7. HFFs were scraped and passed through a 27-gauge needle (SAI Technologies B27-50) to release parasites into fresh HBSS at room temperature. Calcium ionophore A23187 (Sigma C7522) at a final concentration of 1 μM was added to

the sample at room temperature for 10 minutes. Parasites were pelleted, access liquid was aspirated, and parasites were resuspended in the remaining liquid (≈25 µl) prior to loading into 5 µm-diameter glass capillaries. Parasites inside capillaries were then vitrified by fast plunge-freezing in 90 K liquid propane. Capillaries were imaged using the XM-2 cryo soft X-ray microscope (SXM) at the National Center for X-Ray Tomography at the Advanced Light Source (Lawrence Berkeley Laboratories, Berkeley, CA). The XM-2 is equipped with a micro zone plate (MZP) with a spatial resolution of 60 nm, and the imaged capillary was in an atmosphere of helium gas stream cooled by liquid nitrogen. To have a full rotated tomographic dataset reconstructed, 92 projection images were taken with 2 degree increments. The exposure time of each projection varied between 200-450 ms, depending on the beam flux and the sample thickness. Projection images were normalized and aligned, and the tomographic reconstructions were calculated using iterative reconstruction methods in the AREC-3D package [31]. Additional information on the soft X-ray tomography method is available within reference [32].

5. Image Analysis

Automated molecule detection and tracking (Supplementary Figure S2) was performed on raw movies of halo-actin and MLC1-halo molecules in live parasites using u-track software (release 2.2.0) made available by the Danuser lab [6] and run through MATLAB R2019a from Mathworks, Inc. Key u-track parameters were as follows: $\alpha = 0.05$ (sets spot detection threshold relative to background); Gaussian standard deviation $\sigma = 1.3$ pixels = 130 nm (based on an average measured full width half maximum (FWHM) of 3 pixels for single spots, and the relationship between FWHM and σ for the Gaussian distribution: FWHM $= 2\sqrt{2\ ln2}\ \sigma \approx 2.355\ \sigma$); search radius = 0-9 pixels, corresponding to a maximum allowed speed of 9 pixels / 86 ms = 10.5 µm/s; gap tolerance = 0; minimum track length = 5 frames = 0.43 s. These u-track settings were selected empirically to minimize false positive detection events and linkages (erroneous inclusion of two different molecules in a single trajectory), but they necessarily under-report fast-moving population trajectories. After much discussion and consideration of alternative tracking approaches and algorithms, we came to the realization that every automated algorithm is benchmarked against the "gold standard" of object tracking by the human brain, and that manual human tracking of the fast directional actin trajectories apparent by eye was not only a valid option, but in fact the most accurate one available. Thus, the fast, directional actin population tracks (Figure 1D-F) were obtained by manual spot tracking of raw images with the Manual Tracking plugin [33] within Fiji (ImageJ Version 2.0.0-rc-69/1.52p) [34].

Single molecule trajectory outputs (position over time) from u-track or manual tracking were analyzed using home-written MATLAB programs. To align molecule trajectories from different cells to a "reference cell" and calculate the orientation of molecule velocities with respect to cell polarity, cell orientation was determined and trajectories were rotated as follows. First, cell anterior-posterior polarity was determined for every movie analyzed using either subpellicular microtubule imaging or by tracking the cell posterior end following twirling events, in which the posterior end can be identified as the end in contact with the substrate. We note that posterior nuclear position proved to be a consistent polarity indicator as well; while we did not consider nuclear position alone sufficient information to include a cell in this polarity analysis, polarity information from nuclear position analysis was always in agreement with microtubule imaging or twirling analysis. Second, within a home-written MATLAB program, cells were segmented using a threshold determined by Otsu's method (MATLAB Image Processing Toolbox) and fit with an ellipse (regionprops; MATLAB Image Processing Toolbox). Ellipse centroid and orientation (combined with manually annotated polarity info, giving the ellipse an anterior-posterior polarity) were used to determine a rotation matrix that, when applied to cell images or molecule trajectories, aligned them to a "reference cell" with anterior (apical) end pointing up.

Duration and number of reversals during bidirectional patch gliding (Figure S1) and velocities of recirculating F-actin bundles created by jasplakinolide treatment (Figure S4) were manually tracked

using the Manual Tracking plugin [33] within Fiji (ImageJ Version 2.0.0-rc-69/1.52p) [34]. Tracks were analyzed in MATLAB (Mathworks, Inc).

All analyses described above were performed on raw images. For display in figures and movies, images were denoised using noise2void [35].

6. Theoretical Model of *Toxoplasma gondii* Actin Filament Self-Organization

6.1. Continuum flocking theory: background and model choice

To describe the collective motion and organization of actin filaments, we repurposed a classic continuum active matter model that was originally developed by John Toner and Yuhai Tu, inspired by the work of Támas Vicsek, to describe the collective behavior of flocking or schooling animals [36, 37, 38]. This class of theoretical models, known as Toner-Tu or flocking theory, describe collections of "dry," polar, self-propelled agents at any lengthscale, from flocks of flying birds to collections of polarized cytoskeletal filaments. In our case, *Toxoplasma gondii* actin filaments at the cell surface are propelled along by an underlying carpet of plus-end-directed myosin motors, whose action we can effectively capture as polarized filament self-propulsion. One final consideration for us, in choosing a useful and appropriate continuum model for our system, was the distinction between so-called "wet" and "dry" active matter models. In wet active matter systems, the total momentum of self-propelled agents and the media they live in is conserved. Dry active matter are systems without momentum conservation, often due to dominant frictional drag during movement along a surface, or between two walls [39]. In our system of interest, actin filaments move through a confined space ≈ 25 nm in height between the *T. gondii* plasma membrane and the rigid, intermediate filament-reinforced inner membrane complex [1], which we expect to impose a no-slip boundary condition. To make sure that modeling our *T. gondii* actin filament network as a dry, polar system (using Toner-Tu flocking theory) is indeed justified, we now turn to estimating expected viscous drag and friction scales. In the remainder of this subsection, we present this estimate and find that frictional drag indeed dominates. In the subsection that follows, we move on to the details of the Toner-Tu model and its adaptation for our purposes.

One way to formalize the distinction between wet and dry active matter is in terms of a hydrodynamic length scale,

$$l = \sqrt{\frac{\eta}{\gamma}} \tag{1}$$

which reflects a competition between the viscosity η and a friction coefficient γ. In dry active matter systems dominated by frictional drag, l is very small. In a sense, hydrodynamic "communication" through the intervening fluid medium is only possible over very short distances and can thus be neglected.

In our case, actin filament dynamics play out within an extremely thin layer (≈ 25 nm thick) above a no-slip boundary. We consider the magnitude of viscosity and of friction for the intervening fluid (cytoplasm) surrounding our actin filaments. We take $10^{-3}\,\mathrm{N{\cdot}s/m^2}$, the viscosity of water, as a reasonable estimate for η. Next, we require an estimate for the parameter γ, given our knowledge of actin filament dynamics and subcellular structure at the *Toxoplasma* cell surface. We present this estimate in Appendix Section 10.1. In brief, by understanding in-plane frictional drag as capturing viscous interactions across the height of a thin three-dimensional flow, we estimate a friction coefficient $\gamma \approx 10^{13}\,\mathrm{N \cdot s/m^4}$.

Following eqn. 1, we estimate a hydrodynamic length scale

$$l \approx \sqrt{\frac{10^{-3}\,\frac{\mathrm{N \cdot s}}{\mathrm{m^2}}}{10^{13}\,\frac{\mathrm{N \cdot s}}{\mathrm{m^4}}}} \approx 10^{-8}\,\mathrm{m} \tag{2}$$

or 10 nm, a distance smaller than the scale of our agents (filaments) themselves, or their estimated spacing. Thus, we choose a dry, polar active matter model (Toner-Tu flocking theory) to describe the collective motion and self-organization of actin filaments at the parasite surface.

6.2. A minimal Toner-Tu flocking theory

The continuum flocking equations developed by Toner and Tu use two field variables to characterize the evolution of a flock (a collection of birds, or sheep, or actin filaments) in space and time: density $\rho(\mathbf{r}, t)$ and velocity $\mathbf{v}(\mathbf{r}, t)$. Rather than keeping track of each discrete agent (every bird, sheep, or filament) over time, we use the powerful approach of coarse-graining. In our case, this means "dividing up" the *Toxoplasma gondii* cell surface into a continuous field of boxes, or area elements, and identifying an average actin filament velocity and density for each box. This continuum approach enables us to predict the time-evolution and emergence of cell-scale actin filament patterns in the language of our field variables, filament density ρ and velocity $\mathbf{v}$. Mathematically, the evolution of our two field variables in space and time is carried out in the language of partial differential equations. We can think of these partial differential equations as "update rules" that tell us how to use our knowledge of the field variables $\rho(\mathbf{r}, t)$ and $\mathbf{v}(\mathbf{r}, t)$ at a time t and work out their subsequent values $\rho(\mathbf{r}, t + \Delta t)$ and $\mathbf{v}(\mathbf{r}, t + \Delta t)$ at time $t + \Delta t$. This update process repeats, one time step after another, to get the full space-time history of filament density and velocity on the cell surface. We note that, in acknowledgement that our actin filaments of interest are confined in a thin layer between the inner membrane complex and the plasma membrane, our density and velocity fields are strictly two-dimensional, confined to the tangent plane of the cell surface.

With field variables in hand, we write the governing field equations (i.e. partial differential equations, or "update rules") that describe their spatiotemporal evolution. The first governing equation is the continuity equation,

$$\frac{\partial \rho}{\partial t} + \nabla \cdot (\rho \mathbf{v}) = 0. \tag{3}$$

Conceptually, all that this equation says is that if we consider an infinitesimal box of material, the change in the amount of actin within that little box is the difference between the amount that flows in and the amount that flows out. For the moment we consider the case of strict mass conservation with no sources or sinks, though we will see below that the biology of *Toxoplasma* requires us to soften that constraint and allow for both polymerization and depolymerization of actin filaments. The second governing equation is a minimal representation of the dynamics of the $\mathbf{v}$ field offered by Toner and Tu and given first in direct notation by

$$\frac{\partial \mathbf{v}}{\partial t} = [\alpha(\rho - \rho_c) - \beta|\mathbf{v}|^2]\mathbf{v} + D\nabla^2\mathbf{v} - \sigma\nabla\rho - \lambda\mathbf{v} \cdot \nabla\mathbf{v} \tag{4a}$$

and then, equivalently, in index notation by

$$\frac{\partial v_i}{\partial t} = [\alpha(\rho - \rho_c) - \beta v_j v_j]v_i + D\nabla^2 v_i - \sigma\frac{\partial \rho}{\partial x_i} - \lambda v_j \frac{\partial v_i}{\partial x_j}, \tag{4b}$$

where ρ_c is the critical density above which the filaments move coherently, the square root of the ratio of $\alpha(\rho - \rho_c)$ and β sets a filament transport speed scale $|\mathbf{v}| = \mathrm{v}_{\mathrm{myosin}}$, the coefficient D tunes filament alignment with neighbors, $\sigma\nabla\rho$ provides an effective pressure that keeps filament density within a physical range, and the coefficient λ tunes filament velocity self-advection. Note that we are using the summation convention, which tells us to sum over all repeated indices; for example, $\mathbf{a} \cdot \mathbf{b} = a_i b_i = \sum_{i=1}^{3} a_i b_i$.

As illustrated in Figure 2 and Supplementary Figure S3, these terms are a mathematical encapsulation of the "rules" of local filament interactions with other filaments, with myosin motors, and with the geometry of their environment. Together, they provide an update to filament velocity, over each increment in time. Put differently, each term captures a "driving force" that acts locally on filament velocity. The first term on the right, preferred speed, sets the characteristic speed of the actin filaments at 5 μm/s, the approximate mean speed of the relevant myosin motor, TgMyoA (Figure 1 and [40]). The second term, neighbor coupling, involving the Laplacian, we can think of as a velocity smoothing term which pushes a given filament's velocity (orientation and speed) to better match that of its neighbors. For the case of *T. gondii* actin filaments, this neighbor alignment term captures the action of the filament cross-linking

protein coronin [20, 41, 18] and polar alignment through physical collision of moving filaments, as seen *in vitro* [42]. The third term is a pressure term that punishes gradients in density, keeping filament density within a realistic range.

The final term, velocity advection, has analogy to the gradient component of the material time derivative in the Navier-Stokes equations. In essence, the velocity field advects itself; filaments move along in a direction dictated by their orientation and velocity, and they bring that orientation and velocity with them. In the simple case of pure self-advection, $\lambda = 1$. For active agents, such as birds in a flock, λ maybe be further tuned by the behavioral response of the agents to gradients in velocity. In other words, $\lambda = 1 + \xi$, where ξ reflects a behavioral "gradient penalty"; for example, birds may resist flying quickly into a steep gradient of decreasing velocity, and may slow down. For the case of *T. gondii* actin filament self-organization, we choose $\lambda = 1$ as discussed in Section 7.2, because our knowledge of this myosin-driven actin network does not suggest an active ability of filament velocity to respond to nearby gradients in velocity. However, we maintain the advection coefficient λ in our general presentation of the theory, for consistency with the Toner-Tu tradition and to provide the most generally useful version of these minimal flocking equations.

Each term of the four terms in these equations is necessary to capture the basic phenomenology of flocking. In the absence of the preferred speed term or the velocity advection term, filaments do not move. In the absence of the neighbor coupling term, they remain disordered, with no collective motion. In the absence of the pressure term, aphysical densities emerge (negative densities, or densities higher than a cystalline packing of filaments).

We hope that the discussion thus far has clearly presented the mathematics and the intuition of a minimal Toner-Tu flocking theory. We refer the interested reader to reference [8] for additional discussion of this theory, its formulation in a general surface form using extrinsic differential geometry, and a numerical exploration of flocking solutions on varied curved surface shapes. Here, we next extend the minimal flocking theory with new terms specific to *Toxoplasma gondii* actin biology and present our full model for actin filament self-organization on the surface of *Toxoplasma*.

6.3. Extending flocking theory to capture Toxoplasma actin biology: curvature

The original Toner-Tu flocking theory was motivated largely by the motions of animals at macroscopic scales. For the actin filament self-organization in *Toxoplasma*, we introduce several new terms in the velocity and density "update rules." First, we acknowledge that for our actin filaments of interest, confined between the parasite plasma membrane and the inner membrane complex, there is an energetic penalty for filaments whose locally curved environment induces filament bending. Indeed, for actin filaments of approximately 100 nm in length (l), with a persistence length (L_p) $\approx$ 15 μm lying at the surface of a cell of radius R $\approx$ 1 μm, we estimate a bending energy per filament (see eqn. 10.5 in [43]) of

$$E_{bend} \approx \frac{L_p\, l}{2R^2} k_B T \approx 1\, k_B T \approx 4\,\text{pN} \cdot \text{nm}, \tag{5}$$

comparable to thermal energy and to the rough energy scale of a myosin step (a few pN·nm, estimated from a stall force of $\approx$ 0.5 pN [44] and a step size of 5 nm [40]). Given a density of 100 filaments per μm^2 of membrane, this gives us a non-negligible energy of several hundred pN·nm per μm^2. Thus, our first new term captures a driving force that rotates filaments away from the local direction of maximal curvature, favoring filament alignment in the least-curved orientation.

In the remainder of this subsection, we derive this curvature penalty term. We seek to describe the dynamics of reorientation of the filaments as they move away from the unfavored direction of maximum curvature. A phenomenological example of a curvature term that leads to reordering of the actin filaments was used in the work of Woodhouse and Goldstein on filament ordering in the giant internodal cells of algae *Chara* [45]. We consider a similar term, which describes the relaxation dynamics of filament rotation away from the direction of maximum curvature, toward the direction of minimum curvature. Conceptually,

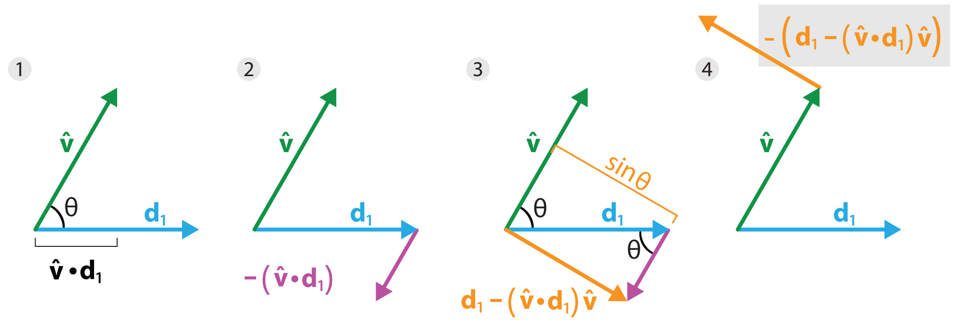

Figure S9. The geometric component of the curvature penalty term. This sequence provides geometric intuition for the "direction of rotation" vector which, when added to the filament velocity vector $\mathbf{v}$, will rotate it away from the direction of maximum curvature $\mathbf{d}_1$ without changing the magnitude of $\mathbf{v}$. In step 1, we note the magnitude of the dot product of two unit vectors, $(\hat{\mathbf{v}} \cdot \mathbf{d}_1)$. The pink vector in step 2 has this magnitude, while pointing in direction -$\mathbf{v}$. In step 3 we note that the orange vector, the sum of the blue and pink vectors, has magnitude $\sin\theta$. Step 4 shows the desired "direction of rotation" vector, which acts on $\mathbf{v}$ to rotate it away from the direction of maximum curvature. We note that in the full curvature penalty term, eqn. 7, this vector is scaled by the kinetic coefficient and elastic driving force, $\varepsilon \cdot F_\kappa$.

this curvature penalty term has three components,

$$\text{curvature update} = (\text{kinetic coefficient}) \cdot (\text{elastic driving force}) \cdot (\text{direction of rotation}). \tag{6}$$

The kinetic coefficient, which we will call ε, is a constant that tunes the contribution of the curvature term to the full Toner-Tu dynamics. The elastic driving force, which we will call $F_\kappa(\mathbf{v}, \kappa)$, computes a curvature-dependent force magnitude as a function of the orientation of vector $\mathbf{v}$ and the local curvature. Lastly, the "direction of rotation" is a geometric function that computes a vector which is orthogonal to the current filament velocity vector $\mathbf{v}$ and which, when added to $\mathbf{v}$, will rotate it away from the direction of maximum curvature $\mathbf{d_1}$ without changing the magnitude of $\mathbf{v}$. As illustrated in Figure S9, this geometric function is given by $-(\mathbf{I} - (\hat{\mathbf{v}} \otimes \hat{\mathbf{v}})\mathbf{d_1}) = -(\mathbf{d}_1 - (\hat{\mathbf{v}} \cdot \mathbf{d}_1)\,\hat{\mathbf{v}})$, where $\hat{\mathbf{v}}$ is a unit vector pointing in the direction of $\mathbf{v}$. Mathematically, our curvature penalty term is therefore given by

$$\left(\frac{\partial \mathbf{v}}{\partial t}\right)_{curv} = -\,\varepsilon\; F_\kappa(\mathbf{v}, \kappa)\,(\mathbf{I} - (\hat{\mathbf{v}} \otimes \hat{\mathbf{v}})\mathbf{d_1})\,. \tag{7}$$

We now focus on $F_\kappa(\mathbf{v}, \kappa)$, the curvature-dependent elastic driving force. To define an expression for F_κ, we follow a long-standing tradition in statistical physics in which the driving force is related to the rate of change of the free energy with respect to the geometric degrees of freedom. For simplicity, we describe the orientation of filaments by the single parameter θ, the angle measured relative to the direction of maximum curvature, $\mathbf{d}_1$. Thus, our starting point is the dynamical equation [46]

$$\frac{\partial \theta}{\partial t} = -\Gamma \frac{\partial (E_{bend})}{\partial \theta}, \tag{8}$$

a kind of relaxation dynamics which assigns a linear relationship between the temporal update of the geometric coordinate θ and the rate of change of the free energy E_{bend} with respect to θ (the driving force). We can then define this energy for all orientations of an actin filament of length L using an equation for elastic beam bending [47, 43],

$$E_{\text{bend}}(\theta) = \frac{EIL}{2}\kappa^2, \tag{9}$$

in which a beam of length L is bent into an arc of circle of radius R with curvature $\kappa = 1/R$. The combination EI is known as the flexural rigidity and is the product of a material factor E (the Young modulus) and a geometric factor I (the areal moment of inertia).

For a general surface, the curvature tensor can be written in diagonal form as

$$\boldsymbol{\kappa} = \begin{bmatrix} \kappa_1 & 0 \\ 0 & \kappa_2 \end{bmatrix} \tag{10}$$

where κ_1 and κ_2 are the maximum and minimum curvatures, respectively, and correspond to two orthogonal directions, $\mathbf{d}_1$ and $\mathbf{d}_2$. To be clear, we can rewrite $\kappa_1 = 1/R_1$ and $\kappa_2 = 1/R_2$, where R_1 and R_2 are the radii of the circles that would best mimic our surface of interest.

To determine the bending energy as a function of filament orientation, we must know $\kappa(\theta)$, the local curvature specifically in the direction that the velocity vector is currently pointing. This quantity is a weighted average of the two principal curvatures and can be written as [48]

$$\kappa(\theta) = \kappa_1 \cos^2\theta + \kappa_2 \sin^2\theta. \tag{11}$$

Thus, the bending energy as a function of the filament orientation θ is given by

$$E_{\text{bend}}(\theta) = \frac{EIL}{2}(\kappa_1 \cos^2\theta + \kappa_2 \sin^2\theta)^2. \tag{12}$$

We now recall from eqn. 8 that the driving force we seek is linearly related to the rate of change of energy with respect to filament orientation, leading us to

$$\frac{\partial\theta}{\partial t} = -\Gamma\frac{\partial(E_{\text{bend}})}{\partial\theta} = -2\Gamma EIL\ (\kappa_1 \cos^2\theta + \kappa_2 \sin^2\theta)\ (\kappa_2 - \kappa_1)\cos\theta(-\sin\theta). \tag{13}$$

Folding Γ, E, I, and L into the kinetic coefficient ε leads us to

$$\frac{\partial\theta}{\partial t} = \varepsilon(\kappa_1 \cos^2\theta + \kappa_2 \sin^2\theta)(\kappa_2 - \kappa_1)\cos\theta\sin\theta, \tag{14}$$

the curvature-dependent driving force which we recall reflects a reduction in the free energy of bending due to this realignment. Finally, we combine this driving force with the direction of rotation vector, $-(\mathbf{I} - (\hat{\mathbf{v}} \otimes \hat{\mathbf{v}})\mathbf{d_1})$. We note that, as shown in Figure S9, this vector has a magnitude of $\sin\theta$. In the final combined curvature term, we want this geometric vector to be of unit length, so that the magnitude of the curvature update is set solely by the kinetic coefficient and elastic driving force. Dividing by $\sin\theta$ and translating back into the language of $\mathbf{v}$, recalling that $\cos\theta = \hat{\mathbf{v}} \cdot \mathbf{d}_1$, leads to

$$\left(\frac{\partial\mathbf{v}}{\partial t}\right)_{curv} = -\varepsilon\ \left(\kappa_1 (\hat{\mathbf{v}} \cdot \mathbf{d}_1)^2 + \kappa_2 (1 - (\hat{\mathbf{v}} \cdot \mathbf{d}_1))^2\right)(\kappa_2 - \kappa_1)(\hat{\mathbf{v}} \cdot \mathbf{d}_1)(\mathbf{I} - (\hat{\mathbf{v}} \otimes \hat{\mathbf{v}})\mathbf{d_1}). \tag{15}$$

To reach the convenient shorthand used in eqn. 7, we can define

$$F_\kappa(\mathbf{v}, \kappa) = \left(\kappa_1 (\hat{\mathbf{v}} \cdot \mathbf{d}_1)^2 + \kappa_2 (1 - (\hat{\mathbf{v}} \cdot \mathbf{d}_1))^2\right)(\kappa_2 - \kappa_1)(\hat{\mathbf{v}} \cdot \mathbf{d}_1). \tag{16}$$

We note also that our use of the commercial finite element method software COMSOL Multiphysics® makes implementing this term relatively straightforward, as the maximum and minimal principal curvature directions and the maximum and minimum curvatures are built-in geometric variables. For the convenience of COMSOL users among our readers, we translate our variable names into the COMSOL built-ins: $\kappa_1 = |\text{curv1}|$; $\kappa_2 = |\text{curv2}|$; $\mathbf{d}_1 = (\text{tcurv1x, tcurv1y, tcurv1z})$. We note that COMSOL syntax for the directions of maximal vs. minimal curvature depends on the defined direction of the normal vector for a given surface geometry.

The full dynamics of the velocity field in the *Toxoplasma* actin self-organization theory, now amended to account for the curvature reorientation dynamics, is given by

$$\frac{\partial\mathbf{v}}{\partial t} = [\alpha(\rho - \rho_c) - \beta|\mathbf{v}|^2]\mathbf{v} + D\nabla^2\mathbf{v} - \sigma\nabla\rho - \lambda\mathbf{v} \cdot \nabla\mathbf{v} - \varepsilon F_\kappa(\mathbf{v}, \kappa)(\mathbf{I} - (\hat{\mathbf{v}} \otimes \hat{\mathbf{v}})\mathbf{d_1}). \tag{17}$$

Toy Model of the Curvature Force: The Cylinder

For the interested reader, in the remainder of this sub-section we consider a toy model of curvature-driven filament alignment on the surface of a simpler shape: a cylinder. This allows us to derive a specific expression for the elastic driving force in terms of the cylinder radius, R, rather than in the language of two arbitrary principle curvatures. On a cylindrical surface, we again characterize the orientation of filaments by the single parameter θ, the angle measured relative to the circumferential direction of the cylinder. We can also define a specific curvature tensor for the cylinder,

$$\boldsymbol{\kappa} = \begin{bmatrix} 0 & 0 \\ 0 & \frac{1}{R} \end{bmatrix}. \tag{18}$$

Recalling that to work out the curvature in any direction $\mathbf{d}$ other than that of the principal curvatures, we can use the weighted average already given above as

$$\kappa = \mathbf{d}^T \boldsymbol{\kappa} \mathbf{d} = \kappa_1 \cos^2\theta + \kappa_2 \sin^2\theta. \tag{19}$$

Using this averaging operation, we find the curvature when the actin filaments are oriented at an angle θ with respect to the circumferential direction as

$$\kappa(\theta) = \frac{1}{R}\cos^2\theta. \tag{20}$$

We see that this has the right limits in that for $\theta = 0$ we recover our usual notion of the radius of the cylinder and for $\theta = \pi/2$, we find that the curvature is zero, corresponding effectively to a radius of curvature that goes to infinity.

We can now find the energy for all orientations of the actin filaments of length L, Young modulus E, and areal moment of inertia I, oriented at an angle θ, using

$$E_{bend}(\theta) = \frac{EIL}{2}\kappa^2 = \frac{EIL}{2R^2}\cos^4\theta. \tag{21}$$

Given this energy as a function of the geometric coordinate θ, we can now compute the change in free energy during reorientation of the filaments as

$$\frac{\partial(E_{bend})}{\partial\theta} = \frac{EI}{2R^2}4L\cos^3\theta(-\sin\theta). \tag{22}$$

Finally, given this expression for the energy, we can compute the dynamical equation for the evolution of θ using eqn. 8, resulting in

$$\frac{\partial\theta}{\partial t} = \Gamma\frac{2EIL}{R^2}\cos^3\theta\sin\theta. \tag{23}$$

To get a feeling for this, Figure S10 shows how the driving force depends upon the angle θ. The blue curve ($\kappa_1 = 1$ μm^{-1}; $\kappa_2 = 0$ μm^{-1}) corresponds to the driving force for a cylinder. The other curves illustrate how the driving force's dependence on θ changes for different geometries, where the maximum and minimum curvatures are not as different.

6.4. Extending flocking theory to capture Toxoplasma actin biology: filament polymerization and depolymerization

Our second addition to the minimal Toner-Tu theory are density source and sink terms, which are usually not included for macroscopic flocks or herds. Although the total number of animals in a school, flock, or herd can of course change over time as animals are born and die, the time scales over which

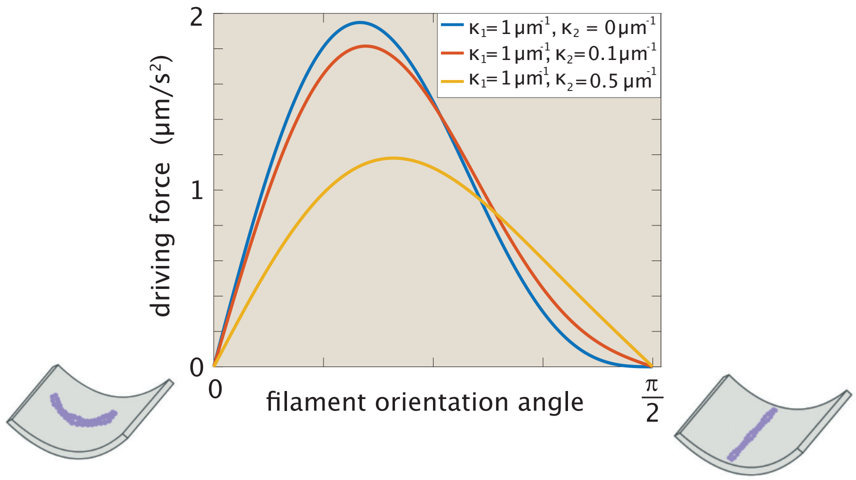

Figure S10. Physics of reorientation of actin filaments due to curvature. The graph shows the driving force for reorientation ($\partial\theta/\partial t$, eqn. 14, with $\varepsilon = 6\ \mu\mathrm{m}^3/\mathrm{s}^2$) as a function of the filament orientation angle θ with respect to the direction of maximum curvature. The blue curve shows the driving force anywhere on a cylindrical surface, where $\kappa_2 = 0$, and θ is defined with respect to the circumferential direction. The red and yellow curves show the driving force at a point on any arbitrary surface with local curvature defined by κ_1 and κ_2.

these changes happen are long compared to the time scale of the flocking behavior itself. They can thus be neglected when accounting for macroscopic changes to density or velocity fields during flocking. In the case of *Toxoplasma* actin filament self-organization, however, both the time scales of actin filament polymerization and depolymerization (see Appendix Section 10.2) and the time scale of filament transport across the entire cell surface are on the order of seconds (see Section 7.2). Moreover, we know that *T. gondii* actin polymerization is favored specifically at the anterior end of the cell (the conoid) by the formin FRM1 [49, 21], and that filaments are further stabilized by the actin-binding protein GAC during anterior microneme secretion events at the anterior end [21]. Conversely, *T. gondii* actin depolymerization is promoted by proteins like profilin [50] and actin depolymerizing factor (ADF) [10], which are not known to be spatially restricted.

In order to mathematicize this biological intuition and capture the short time scale dynamics of actin filaments, we amend the continuity equation. In its simplest form, the continuity equation acknowledges only one way to change the quantity of molecules in a material volume element: by molecules moving in and out of that element, to and from neighboring volume elements. To incorporate the reality of filament polymerization and depolymerization, we must allow sources and sinks of filament density. At the cell anterior, we add both a source term c and a degradation term $-\gamma\rho$, leading to an amended version of the continuity equation that can be written as

$$\frac{\partial \rho}{\partial t} = -\nabla \cdot (\rho \mathbf{v}) - \gamma\rho + c. \tag{24}$$

Actin filament density is added (polymerized) at a constant rate c and lost (depolymerized) at a density-dependent rate $\gamma\rho$. Over the rest of the cell surface, outside the conoid, polymerization is not favored but depolymerization still occurs. Thus,

$$\frac{\partial \rho}{\partial t} = -\nabla \cdot (\rho \mathbf{v}) - \gamma\rho. \tag{25}$$

The Toner-Tu equations in conjunction with these generalized versions of the continuity equation constitute the full conceptual formulation of the problem of actin dynamics for *Toxoplasma*, as summarized in Figure S3.

6.5. On resistive forces from the environment.

The Toner-Tu model of *Toxoplasma gondii* actin self-organization presented in this work explores the collective dynamics of motor-propelled actin filaments without reference to the external environment and potential resistive forces, which could be exerted in a spatially inhomogeneous manner. Is this focus on the myosin-driven actin system itself (internal dynamics) justified, or could forces from the environment, such as frictional drag on the cell membrane, significantly alter actin movement and organization?

To answer this question, we consider possible resistive forces from sources external to the actin system itself: (1) drag from the motion of a gliding cell through fluid, (2) shear stresses due to that motion occurring just above the 'no-slip' boundary condition imposed by the substrate, and (3) drag force from transmembrane adhesin proteins coupled to actin and moving through the plasma membrane. For each resistive force, we perform a rough estimate of its magnitude and in each case, we find that it is on the order of 0.1 pN, smaller than the several pN forces generated by - and needed to stall - a single myosin motor. These estimates are presented in the appendix, in Section 10.3.

These estimates suggest that drag forces external to the myosin-driven actin system contribute a small perturbation to actin filament motion and patterning during gliding through fluid on a two-dimensional substrate. Thus, we hypothesize that an internal model of actin self-organization is justified and useful.

As an aside, we would like to note that once the cell is moving through stiff extracellular matrix or invading through a host cell cortex, we expect that resistive stresses from the environment will exceed the stall force of a single motor. We find it very exciting to hypothesize that feedback between the extracellular environments geometry and mechanical properties and intracellular actin self-organization enables the emergence of different actin patterns (and thus, different motility behaviors) suited to different steps of the infection process, different host tissues, etc. In the future, we hope that we and others will be able to build on the simple self-organization framework presented in this work to explore how diversity in mechanical behavior could arise from coupling that framework to more complex mechanical environments.

7. Parameter Choices and Dimensionless Ratios

One tool for developing intuition for partial differential equations, such as the *Toxoplasma* actin self-organization equations presented above, is to reformulate these governing equations in dimensionless form. In this section, we first explore the dimensionless version of our equations and develop intuition for the dimensionless ratios they present. We then estimate and define parameter values for the specific case of the *Toxoplasma gondii* actin surface layer in "real-world" units. A parallel discussion of the dimensionless Toner-Tu equations and parameter estimates for macroscopic herding animals is presented in our manuscript on wildebeest herding on arbitrary curved surfaces [8].

7.1. Dimensionless ratios in the theory

We seek here to explain in an accessible way the process of reformulating the governing equations in dimensionless form. We begin by explicitly defining the units of the two key field variables ρ and $\mathbf{v}$. The density field describes the number of filaments found per unit area in the thin surface layer of the *Toxoplasma* cells. This implies that we have

$$[\rho] = \frac{1}{L^2}, \tag{26}$$

where we use the notation that $[thing]$ means "units of thing," and L and T are units of length and time, respectively. Similarly, the field $\mathbf{v}$ has units given by

$$[v] = \frac{L}{T}. \tag{27}$$

With these definitions in hand, we can now explore the units of the various terms that appear in the Toner-Tu equations given in eqn. 17, and then define dimensionless variables that will allow us to recast the equations in dimensionless form. The time derivative of the **v**-field results in a quantity with units

$$[\frac{\partial v_i}{\partial t}] = \frac{L}{T^2}. \tag{28}$$

Thus, all the other terms in the Toner-Tu equations must have these same units. We can determine the units of the advection term using

$$[\lambda v_j \frac{\partial v_i}{\partial x_j}] = \frac{L}{T^2}. \tag{29}$$

For this to be true, we must have

$$\lambda \times \frac{L}{T} \times \frac{1}{T} = \frac{L}{T^2} \tag{30}$$

which implies that λ is dimensionless,

$$[\lambda] = 1. \tag{31}$$

To evaluate the units in the velocity selection term we note that we have

$$[\alpha \times \rho \times v] = [\alpha] \times \frac{1}{L^2} \times \frac{L}{T} = \frac{L}{T^2} \tag{32}$$

which implies that

$$[\alpha] = \frac{L^2}{T}. \tag{33}$$

Similar reasoning allows us to determine the units of the quantity β using

$$[\beta v^3] = \beta \frac{L^3}{T^3} = \frac{L}{T^2}. \tag{34}$$

This implies that

$$[\beta] = \frac{T}{L^2}. \tag{35}$$

The neighbor coupling diffusion-like term tells us

$$D \frac{\partial^2 v}{\partial x^2} = D \frac{L/T}{L^2} = \frac{L}{T^2} \tag{36}$$

which gives us the dimensions of D as

$$D = \frac{L^2}{T}. \tag{37}$$

We next tackle the pressure term which requires

$$[\sigma \frac{\partial \rho}{\partial x}] = \sigma \frac{1}{L^3} = \frac{L}{T^2}. \tag{38}$$

This implies that

$$[\sigma] = \frac{L^4}{T^2}. \tag{39}$$

Lastly, the curvature term demands that

$$\varepsilon \frac{1}{L^2} = \frac{L}{T^2} \tag{40}$$

which lead us to conclude that

$$[\varepsilon] = \frac{L^3}{T^2}. \tag{41}$$

We now have a clear picture of the units of each of the material parameters that appears in the Toner-Tu *T. gondii* actin self-organization theory, and we move on to defining our dimensionless variables. For example, for the positional coordinate, we take

$$x^* = \frac{x}{L}, \tag{42}$$

where L is a characteristic length scale in the problem, which we take to be the cell size, $L \approx 5$ μm. Similarly, we use a characteristic velocity scale $U \approx 5$ μm/s, set by myosin A speed, to define the dimensionless velocity as

$$v^* = \frac{v}{U}. \tag{43}$$

The density can be rescaled by using the critical density ρ_c as our scaling variable, resulting in

$$\rho^* = \frac{\rho}{\rho_c}. \tag{44}$$

Recalling that curvature has units of $1/L$, we define our scaled curvature variable through

$$\kappa^* = L\kappa. \tag{45}$$

Lastly, in light of the definitions above, we can define a time scale L/U, the time it takes for a myosin-transported actin filaments to cross the entire cell, culminating in the definition

$$t^* = \frac{t}{L/U}. \tag{46}$$

Using the definitions given above, we can now rewrite the Toner-Tu actin self-organization equations using the dimensionless versions of t, x, ρ and $\mathbf{v}$ as

$$\frac{U^2}{L}\frac{\partial v_i^*}{\partial t^*} = \rho_c U\alpha(\rho^*-1)v_i^* - \beta U^3|\mathbf{v}^*|^2 v_i^* - \frac{\sigma\rho_c}{L}\frac{\partial \rho^*}{\partial x_i^*} + \frac{DU}{L^2}\nabla_*^2 v_i^* - \frac{\lambda U^2}{L}v_j^*\frac{\partial v_i^*}{\partial x_j^*} - \frac{\varepsilon}{L^2}F_\kappa(v^*,\kappa^*)\left((d_1)_i - \frac{v_i^* v_j^*(d_1)_j}{v_k^* v_k^*}\right). \tag{47}$$

We then divide everything by U^2/L, resulting in five dimensionless parameters and full dynamical equations of the form

$$\frac{\partial v_i^*}{\partial t^*} = \frac{\rho_c L\alpha}{U}(\rho^*-1)v_i^* - LU\beta|\mathbf{v}^*|^2 v_i^* - \frac{\sigma\rho_c}{U^2}\frac{\partial \rho^*}{\partial x_i^*} + \frac{D}{UL}\nabla_*^2 v_i^* - \lambda v_j^*\frac{\partial v_i^*}{\partial x_j^*} - \frac{\varepsilon}{LU^2}F_\kappa(v^*,\kappa^*)\left((d_1)_i - \frac{v_i^* v_j^*(d_1)_j}{v_k^* v_k^*}\right). \tag{48}$$

To write the dimensionless version of the continuity equation, we use the same definitions of dimensionless variables given above and the same strategy for replacing dimensionful variables with their dimensionless counterparts. We find that the generalized continuity equation takes the form

$$\frac{\partial \rho^*}{\partial t^*} = -\nabla_* \cdot (\rho^* v^*) - \frac{\gamma L}{U}\rho^* + \frac{L}{U\rho_c}c. \tag{49}$$

Writing the Toner-Tu equations in this form gives us a handle on the meaning of the different terms. The two components of the velocity selection term have dimensionless parameters given by

$$\text{velocity selection } \alpha \text{ term} = \frac{\alpha\rho_c L}{U} = \frac{L/U}{1/(\alpha\rho_c)} = \frac{\text{time for filament to cross cell}}{\text{time for speed to increase to } v_{myosin}} \tag{50}$$

and

$$\text{velocity selection } \beta \text{ term} = \beta LU = \frac{L/U}{1/(\beta U^2)} = \frac{\text{time for filament to cross cell}}{\text{time for speed to decrease to } v_{myosin}}. \quad (51)$$

Each of these terms provides intuition about how quickly the filaments return to the steady state mean-field speed v_{myosin} given some perturbation that disturbs them from that value. Recall that L/U (the "time for filament to cross cell") is roughly the time it takes for an actin filament to be transported by myosin across the length of the cell. The pressure term can be rewritten as

$$\text{pressure term} = \frac{\rho_c}{U^2/\sigma} = \frac{\text{critical density}}{\text{typical density excursion away from } \rho_0}. \quad (52)$$

Increasing σ decreases density variance; in other words, densities that emerge are within a more narrow range around the mean density ρ_0. The neighbor coupling term that carries out democratic velocity smoothing has the dimensionless prefactor

$$\text{democracy term} = \frac{D}{UL} = \frac{L/U}{L^2/D} = \frac{\text{time for filament to cross cell}}{\text{time for velocity to diffuse across cell}}, \quad (53)$$

analogous to the Péclet number. The velocity self-advection term prefactor λ, which for our case is 1, can be written as

$$\text{advection term} = \frac{\lambda L/U}{L/U} = \frac{\text{time for velocity advection across cell}}{\text{time for filament to cross cell (density advection)}}. \quad (54)$$

Finally, the curvature penalty term can be written as

$$\text{curvature penalty ratio} = \frac{\varepsilon}{LU^2} = \frac{L/U}{UL^2/\varepsilon} = \frac{\text{time for filament to cross cell}}{\text{time for filament to rotate away from maximum curvature}}. \quad (55)$$

To interpret this ratio, we remember that $\kappa = 1/L$ is a typical cell curvature, that the curvature-induced elastic driving force scales with κ^2, and that $\mathbf{v}/U$ is a unit vector indicating the *orientation* of a filament. Thus, ε/UL^2 gives a curvature-dependent rate of rotation or relaxation for filament orientation, and UL^2/ε gives a time scale for curvature-dependent rotation.

For each of these cases, the value of the dimensionless ratio gives us a sense of how large a contribution the term of interest will make in the incremental update to $\mathbf{v}(t)$. That is, if we think of the numerical solution to the equations, during every time step Δt, $\mathbf{v}(t)$ will get updated at every point in space. How much $\mathbf{v}(t)$ changes depends upon all the different contributions in the governing equation. These dimensionless ratios measure the relative importance of each term, serving roles analogous to the Reynolds number in thinking about the Navier-Stokes equations and the Péclet number in the context of coupled diffusion-advection problems.

Next, we turn to the two dimensionless ratios in the density governing equation. The term multiplying ρ^* on the right side of eqn. 49 involves the ratio of two very important time scales, namely,

$$\frac{\text{time for filament to cross cell}}{\text{time to depolymerize actin filament}} = \frac{L/U}{1/\gamma}. \quad (56)$$

Intuitively, we expect that when this dimensionless ratio is of order unity or larger ($\gamma > U/L$), then a rearward steady-state flow can be achieved because the actin filaments do not live long enough to accumulate at the cell posterior and force a return orbit.

The actin production rate is also given by a dimensionless ratio of the form

$$\frac{\text{actin density production rate}}{\text{actin density transport rate}} = \frac{c}{\rho_c U/L}. \quad (57)$$

7.2. Parameter choices for modeling Toxoplasma actin self-organization

In this subsection, we estimate parameter values for the specific case of *Toxoplasma gondii* surface F-actin organization, and we define the parameter values used in the simulations reported in the main text. We also discuss the transition from the recirculating to the unidirectional F-actin state, using estimates and calculations to sanity check our parameter choices for filament polymerization and depolymerization. To determine our parameters, we use our knowledge of *Toxoplasma gondii* actin biology, order-of-magnitude estimates, and a principle of balancing terms.

Based on published electron microscopy of actin filaments from gliding *Toxoplasma gondii* [9], we estimate a rough density of 150 filaments/μm^2. This average density will serve as the initial uniform density in our simulations,

$$\rho_0 = 150 \frac{1}{\mathrm{\mu m}^2}. \tag{58}$$

We next estimate ρ_c, the critical density above which actin filaments interact with their neighbors and collectively organize. Actin filaments in *T. gondii* are thought to be of order 100 nm in length [51, 52]. To estimate the critical density, we consider the density scale at which filaments can explicitly interact with neighboring filaments. If we imagine that each filament is free to rotate, sweeping out a circular area with diameter 100 nm, these circles will begin to overlap when more than 10 x 10 filaments are packed within a 1 μm x 1 μm square. Thus, we estimate that filaments collectively organize above a density

$$\rho_c = 100 \frac{1}{\mathrm{\mu m}^2}. \tag{59}$$

To get a feel for this density, picture 10 rows of 10 filaments, each of length 0.1 μm, in a 1 μm by 1 μm square.

We next move to the parameters of the governing equation for velocity, where a principle of balancing terms will play an important role. That is, in order to explore the contributions of all terms in the velocity equations, we balance their magnitudes to ensure that each contributes roughly equally to the overall velocity update. To get an initial estimate of the scale of that update, we estimate the typical change in velocity as

$$\frac{\partial v}{\partial t} \approx \frac{10\ \mathrm{\mu m/s}}{1\ \mathrm{s}} \approx 10\ \mathrm{\mu m/s^2}, \tag{60}$$

considering that a filament reaching the end of the cell at roughly 5 μm/s will reverse directions (and head back up the cell at velocity -5 μm/s) over a time scale of roughly one second. Hence, the acceleration is estimated to be ≈ 10 $\mathrm{\mu m/s^2}$. Now that we have this as the typical scale of the change in velocity, we can examine each term in the governing equation and determine its material parameter by demanding that it yield a contribution of comparable magnitude. Let's try this out for the velocity selection terms. First, we have

$$10\ \mathrm{\mu m/s^2} = \alpha(\rho - \rho_c)\mathrm{v} \approx \alpha \times 50\ \frac{1}{\mathrm{\mu m}^2} \times 5\ \mathrm{\mu m/s} \tag{61}$$

which leads to the conclusion that

$$\alpha = 0.04\ \mathrm{\mu m^2/s}. \tag{62}$$

To determine the parameter β we adopt the same strategy, with

$$10\ \mathrm{\mu m/s^2} = \beta \mathrm{v}^3 \approx \beta \times (5\ \mathrm{\mu m/s})^3 \tag{63}$$

which leads to the conclusion that

$$\beta = 0.08\ s/\mu m^2. \tag{64}$$

We can now apply this thinking to make an estimate of the neighbor coupling coefficient using the equality

$$10\ \mathrm{\mu m/s^2} = \mathrm{D}\frac{\partial^2 \mathrm{v}}{\partial \mathrm{x}^2} \approx \mathrm{D}\frac{5\ \mathrm{\mu m/s} - (-5\ \mathrm{\mu m/s})}{(1\ \mathrm{\mu m})^2}, \tag{65}$$

where we again imagine a change in filament velocity from 5 μm/s to -5 μm/s and take 1 μm to be the length scale of such gradients in the velocity, leading to an estimate for the neighbor coupling coefficient of

$$D = 1\ \mu\text{m}^2/\text{s}. \tag{66}$$

To find the parameter σ that tunes the pressure term, we estimate a density gradient scale of $\rho - \rho_c = 50\ 1/\mu\text{m}^2$ and make the correspondence

$$10\ \mu\text{m/s}^2 = \sigma\frac{\partial\rho}{\partial \text{x}} \approx \sigma\frac{50\ \frac{1}{\mu\text{m}^2}}{1\ \mu\text{m}} \tag{67}$$

which leads to a choice of

$$\sigma = 0.2\ \mu\text{m}^4/\text{s}^2. \tag{68}$$

Our final term in the Toner-Tu equations themselves concerns the penalty for filament alignment in a direction of high curvature. Recalling eqn. 15 and estimating values of 4 μm^{-1} for κ_1, 1 μm^{-1} for κ_2, and $1/\sqrt(2)$ for $\frac{\mathbf{v}}{|\mathbf{v}|}\cdot\mathbf{d}_1$, we have

$$10\ \mu\text{m/s}^2 \approx -\varepsilon(2.5\frac{1}{\mu\text{m}})(1\frac{1}{\mu\text{m}} - 4\frac{1}{\mu\text{m}})(\frac{1}{\sqrt{2}})(1-\frac{1}{\sqrt{2}}) \tag{69}$$

which leads us to

$$\varepsilon = 6\ \mu\text{m}^3/\text{s}^2. \tag{70}$$

The advection term warrants special discussion. Following the tradition of Toner and Tu [37], the dimensionless coefficient λ does not need to be equal to 1 for the case of flocking animals. If $\lambda = 1$, this term simply enforces the material time derivative; in other words, the velocity field advects itself, as in the Navier-Stokes equation. Flocking birds or sheep, however, can display behaviors - like slowing down when they notice a gradient of decreasing velocity in the birds ahead of them - that modify the parameter λ away from a value of 1. In order to show the theory in its most generally useful form, we have maintained λ throughout as a tunable parameter. However, for our simulations of actin filament self-organization, we do not hypothesize any behavioral response to the velocity gradient, as described above for bird flocks. Thus, we have

$$\lambda = 1. \tag{71}$$

Using a principle of balancing terms, we have made a first guess at each of the parameters appearing in the Toner-Tu formulation for *Toxoplasma*. Note that the main purpose of the use of the Toner-Tu theory in the context of our work on *Toxoplasma* was to understand what kinds of behaviors are possible. From that point of view, getting a sense of the characteristic scales of the different parameters suffices. That said, we can imagine explicit experimental strategies aimed at measuring and ultimately controlling the parameters in the theory, and we believe this represents an exciting direction for future work.

Finally, we turn to estimating the parameters c and γ of the equation for actin filament density,

$$\frac{\partial\rho}{\partial t} = -\nabla\cdot(\rho\mathbf{v}) - \gamma\rho + c, \tag{72}$$

where the two terms on the far right govern depolymerization and polymerization of filaments, respectively. From the previous section, we remember a key dimensionless ratio,

$$\frac{\text{time for filament to cross cell}}{\text{time to depolymerize actin filament}} = \frac{L/U}{1/\gamma}. \tag{73}$$

Intuitively, we expect that a transition between recirculating and unidirectional F-actin flow could arise when this dimensionless ratio is of order unity. Thus, we can estimate that

$$\gamma = \frac{U}{L} \approx \frac{5\ \mu\text{m/s}}{5\ \mu\text{m}} \simeq 1\ \text{s}^{-1}, \tag{74}$$

or a bit smaller, given our rounding down of cell length. In Figure 4, we find interesting changes in behavior as we sweep through a range of values for γ from 0.2 s^{-1} to 0.7 s^{-1}, and in our estimate for c below, we use a value of $\gamma \approx 0.5\ s^{-1}$.

The actin filament polymerization rate is also given by a dimensionless ratio of the form

$$\frac{\text{actin density production rate}}{\text{actin density transport rate}} = \frac{c}{\rho_c U/L}. \tag{75}$$

A rough estimate of the parameter c can be gotten by considering a simplified scenario in which density is uniform across the cell surface, and is in steady state, unchanging in time. Importantly, we must remember that actin polymerization is promoted specifically at the cell anterior, in the *T. gondii* conoid [49, 21]. In our model, filament density production at rate c is restricted to the conoid region, as shown in Supplementary Figure S3. This region has a surface area A_{conoid}, which is approximately 1/20th of A_{tot}, the total surface area of the cell. Thus, the steady state density balance for filaments will take the form

$$-\gamma \rho_{ss} A_{tot} + c A_{conoid} = 0. \tag{76}$$

We can solve this for the steady-state filament density,

$$\rho_{ss} = \frac{c}{\gamma} \frac{A_{conoid}}{A_{tot}}. \tag{77}$$

Setting the steady-state filament density equal to our estimated average density, ρ_0, we obtain an estimate for our filament polymerization coefficient,

$$c = \gamma \rho_0 \frac{A_{tot}}{A_{conoid}} \approx 0.5\ \text{s}^{-}1 \times 150\ \mu\text{m}^{-}2 \times 20 \approx 1500\ \mu\text{m}^{-2}\ \text{s}^{-1}. \tag{78}$$

We use this value for c in Figure 4A, and in Figure 4B we sweep through a range of values for c from 0 to 2000 $\mu m^{-2}\ s^{-1}$. In Section 10.2 of the Appendix, we compare the rates of filament polymerization and depolymerization determined above to data and estimates on actin dynamics in well-studied biological systems, and we find them to be similar. While the rates proposed above seemed large to us at a first glance, the more considered deliberation presented in the Appendix convinced us that these rates are in a reasonable range.

8. Deriving a Tangential Formulation of the Filament Self-Organization Equations for a Curved Surface

Thus far, our treatment of the actin filament self-organization equations presented here has ignored the fact that in our *T. gondii* case of interest, the "flock" of actin filaments is moving around on the curved surface of the cell. Our mathematics must account for this geometry; for example, the evaluation of the derivatives used in the governing partial differential equations requires a knowledge of the local curvature. In this section, we recast our governing equations in a tangential formulation for curved surfaces, using an extrinsic differential geometry approach rather than a more traditional intrinsic one. The presentation here is abridged; for a more thorough discussion of this general curved-surface reformulation of the Toner-Tu theory, we refer the reader to our manuscript on animal herding on arbitrary curved surfaces [8].

Traditional formulations of the differential geometry of surfaces begin with the parametrization of a surface of interest using a series of points given by $\mathbf{r}(u, v)$, where u and v are the parameters that

characterize the surface of interest. The surface of a cylinder of radius R, for example, can be represented by

$$\begin{aligned}\mathbf{r}(\theta, z) &= (x(\theta, z), y(\theta, z), z(\theta, z)) \\ &= (R\cos\theta, R\sin\theta, z).\end{aligned} \tag{79}$$

However, the complex shape of the *Toxoplasma gondii* cells of interest here does not conform to the simple parameterization of highly idealized geometries. While motile *Toxoplasma gondii* tachyzoite shape is stereotypical and consistent across cells, it is highly asymmetric, with features like a crescent-like bend and a narrow protrusion (the conoid) at the anterior cell end. In order to understand the contributions of the real-world, biological geometry of these cells, we want to solve our self-organization equations on their true asymmetric shapes. This is clearly not possible analytically.

To move toward an ultimate goal of solving our equations numerically using the finite element method, we can reformulate them using an extrinsic differential geometry approach; we carry out the mathematics in the full three-dimensional setting of $\mathbb{R}^3$, while using a knowledge of the normal vector $\mathbf{n} = (n_1, n_2, n_3)$ everywhere on the surface of interest to project the governing equations onto the surface. For a full description of the tangential surface formulation of flocking governing equations on an arbitrary curved surface, the reader is directed to our recent manuscript [8]. Central to this extrinsic geometry approach is the projection operator, defined as

$$P_{ij} = \delta_{ij} - n_i n_j. \tag{80}$$

The curved space or tangential version of the pressure term $\sigma\nabla\rho$, for example, requires projecting the full 3D gradient of the density onto the tangent plane. We follow Jankuhn *et al.* [53] in introducing the notation ∇_Γ for the projection of the gradient onto the surface of interest. Mathematically, this amounts to computing

$$(\nabla_\Gamma \rho)_i = (\delta_{ij} - n_i n_j)\frac{\partial \rho}{\partial x_j}. \tag{81}$$

We again refer the reader to [8] for a detailed explication of the use of tangential differential operators to generate surface-projected versions of all the other terms in the Toner-Tu equations. Here, we modify the curvature penalty term to its curved-surface implementation, since it is an original contribution to the *Toxoplasma* actin self-organization equations and not included in reference [8]. We first define $\mathbf{v}^{\|}$, the in-plane velocity,

$$v_i^{\|} = P_{ij} v_i = v_i - n_i v_i n_j. \tag{82}$$

The curved-surface formulation of the curvature penalty update, derived for the plane in Section 6.3, can now be written in component form as

$$\left(\frac{\partial v_i^{\|}}{\partial t}\right)_{\Gamma, curv} = -\varepsilon\, F_\kappa(v_i^{\|}, \kappa)\left((d_1)_i - \frac{v_i^{\|} v_j^{\|} (d_1)_j}{v_k^{\|} v_k^{\|}}\right). \tag{83}$$

The tangential curved-surface form of the full governing equations for velocity is

$$\begin{aligned}\frac{\partial v_i^{\|}}{\partial t} &= [\alpha(\rho - \rho_c) - \beta v_j^{\|} v_j^{\|}] v_i^{\|} - \sigma\left(\frac{\partial \rho}{\partial x_i} - n_i n_j \frac{\partial \rho}{\partial x_j}\right) + D P_{il}\left(\frac{\partial G_{lj}}{\partial x_j} - n_j n_k \frac{\partial G_{lj}}{\partial x_k}\right) \\ &\quad - \lambda\left[\left(\frac{\partial v_i^{\|}}{\partial x_j} - n_l n_j \frac{\partial v_i^{\|}}{\partial x_l}\right) - n_i n_k\left(\frac{\partial v_k^{\|}}{\partial x_j} - n_l n_j \frac{\partial v_k^{\|}}{\partial x_l}\right)\right] v_j^{\|} - \varepsilon\, F_\kappa(v_i^{\|}, \kappa)\left((d_1)_i - \frac{v_i^{\|} v_j^{\|} (d_1)_j}{v_k^{\|} v_k^{\|}}\right),\end{aligned} \tag{84}$$

where $\mathbf{d}_1$ is a unit vector in the direction of maximum local curvature, F_κ is defined in eqn. 16, and $\mathbf{G}$ is the surface velocity gradient tensor which in component form can be written as

$$G_{ij} = \left(\frac{\partial v_i^{\|}}{\partial x_j} - n_l n_j \frac{\partial v_i^{\|}}{\partial x_l}\right) - n_i n_k\left(\frac{\partial v_k^{\|}}{\partial x_j} - n_l n_j \frac{\partial v_k^{\|}}{\partial x_l}\right). \tag{85}$$

Similarly, the curved-surface formulation of the governing equation for density can be written as

$$\frac{\partial \rho}{\partial t} = -\frac{\partial(\rho v_i^{\|})}{\partial x_i} + n_i n_j \frac{\partial(\rho v_i^{\|})}{\partial x_j} - \gamma\rho + c. \tag{86}$$

We now have a complete formulation of our self-organization governing equations for the general surface context, requiring only a description of the surface in the language of normal vectors.

9. Numerically Solving the Filament Self-Organization Equations on the *Toxoplasma* Cell Surface

In order to solve the curved-surface formulation of our actin filament self-organization equations for general initial conditions and on general geometries, like the surface of the *Toxoplasma gondii* cell, we turn to the finite element method (FEM). More specifically, we make use of the custom PDE solver capabilities of the commercial FEM software COMSOL Multiphysics® [54]. The finite element method enables us to represent the cell surface as a mesh of triangles and solve our dynamical equations at the nodes of those triangles, then interpolate between them. In this section, we first detail the creation of surface meshes compatible with the finite element method from the soft X-ray tomograms of extracellular *Toxoplasma* tachyzoites described in Section 4.8. We then discuss our use of the finite element method to numerically solve the tangential self-organization equations on these surface meshes.

9.1. Spherical harmonic shape analysis of Toxoplasma cells and mesh generation

Many fields of study are now dependent upon fast and reliable representations of surfaces. One particular tool for doing so, called SPHARM-PDM, converts image segmentations into a mathematical representation of shape in the language of spherical harmonics, and then into triangulated surfaces [55]. This approach has proven useful for shape analysis and comparison in the macroscopic contexts of archaeology and medical imaging [56], and in this study we use it at microscopic scales, to describe the shape of single cells. In brief, the SPHARM tool maps the closed surface of an object of interest (which must have spherical topology) to the surface of a sphere, preserving area and minimizing distortion [55]. This mapping enables a mathematical description of the surface of interest as a series of spherical harmonics [57]. In essence, the SPHARM description is a list of coefficients, which weight a series of basis functions: the spherical harmonics. The spherical harmonics $Y_{lm}(\theta, \varphi)$ are solutions to Laplace's equation on the surface of a sphere and form a "basis" from which we can represent any function parametrized by θ and ϕ as

$$\mathbf{r}(\theta,\varphi) = \begin{bmatrix} x(\theta,\varphi) \\ y(\theta,\varphi) \\ z(\theta,\varphi) \end{bmatrix} = \sum_{l=0}^{\infty}\sum_{m=-l}^{l} \begin{bmatrix} c_{lm}^{(x)} \\ c_{lm}^{(y)} \\ c_{lm}^{(z)} \end{bmatrix} \quad Y_{lm}(\theta,\varphi). \tag{87}$$

All the character of a given closed surface is captured in the coefficients $c_{lm}^{(i)}$, where the superscript i tells us which component of the vector $\mathbf{r}(\varphi, \theta)$ is being considered. In other words, different closed surfaces are described using the same series of harmonics ($Y_{lm}(\theta, \varphi)$), but the series of coefficients $c_{lm}^{(i)}$ will differ, giving more or less weight to a given harmonic in the overall representation of a given surface. To obtain these coefficients, the orthonormality of the spherical harmonics is used to pick off a given coefficient by multiplying both sides of eqn. 87 by $Y_{l'm'}^*(\theta, \varphi)$ and integrating over θ and φ to obtain

$$c_{\mathrm{l'm'}}^{(x)} = \int_0^{2\pi} d\varphi \int_0^{\pi} \sin\theta \; d\theta \quad Y_{l'm'}^*(\theta,\varphi) x(\theta,\varphi). \tag{88}$$

Using these ideas, the surface of a representative *Toxoplasma gondii* tachyzoite cell was broken up into a series of weighted spherical harmonic modes. We note that *Toxoplasma gondii* tachyzoites have

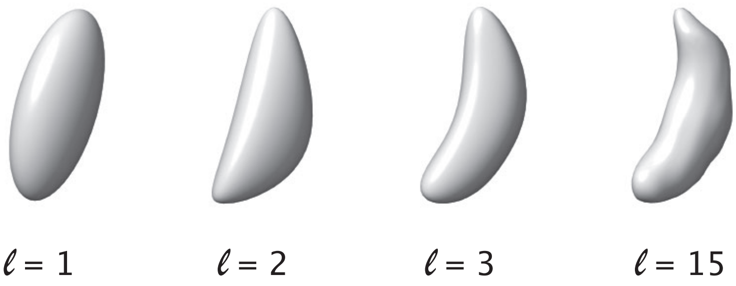

Figure S11. The surface of an extracellular *T. gondii* tachyzoite cell, represented by a series of spherical harmonic modes. Each panel shows the cell shape representation when carried to the order of expansion indicated by the value of ℓ. For example, the right-most representation is $\mathbf{r}(\theta,\varphi) = \sum_{l=0}^{15} \sum_{m=-l}^{l} c_{lm}^{(i)} \; Y_{lm}(\theta,\varphi)$.

a consistent, stereotyped shape and size. First, reconstructed 3-dimensional soft X-ray tomography images of extracellular, activated *T. gondii* tachyzoite cells were segmented in ChimeraX version 1.0 [58] and converted into binary image stacks. Within the 3D Slicer package[59], images of one representative cell were converted to a label map read in by the SPHARM-PDM Generator module, used within the SlicerSalt package [60]. As described above, a SPHARM shape description was generated in the language of spherical harmonics. Including a larger number of modes in the shape description will better capture the original surface shape, as shown in Figure S11. For our representations, we used $\ell = 15$ modes. The surface thus represented was sampled into a triangular mesh (PDM), which is compatible with the finite element method and was used to solve the governing partial differential equations for the density and velocity fields within COMSOL Multiphysics®.

9.2. Solving the tangential self-organization equations in COMSOL Multiphysics®

To solve our custom surface partial differential equations (eqns. 84 and 86), we used the COMSOL Multiphysics® General Form Boundary PDE interface and took advantage of COMSOL's built-in tangential differentiation operator, dtang(f,x). We also made use of the normal vector (nx, ny, nz), a built-in geometric variable, and the curvature variables discussed in Section 6.3. For a thorough and practical introduction to the finite element method, we recommend reference [61]. For additional details on rewriting the curved-surface Toner-Tu equations in a form convenient for standard finite element method solvers and for implementing specifically in COMSOL Multiphysics®, we refer the reader to reference [8]. For each simulation, we initialized with uniform density field $\rho_0 = 150\ 1/\mu\text{m}^2$ and a disorganized velocity field, in which every node's velocity orientation was drawn randomly from a uniform distribution of angles between 0 and 2π and had magnitude $v(0) = 5\ \mu\text{m/s} \approx v_{\text{myosin}}$. Other parameters are defined and discussed in detail in Section 7. Our standard mesh size involved 660-794 triangular elements. To avoid the accumulation of out-of-plane components in $\mathbf{v}$ from numerical error, we implemented a weak constraint of $\mathbf{n} \cdot \mathbf{v} = 0$. Similarly, in the model with no filament turnover, we implemented a global constraint on the total integrated density ρ on the surface, ensuring it stayed at its initial value. Default COMSOL solvers and settings were used: implicit backward differentiation formula (BDF) for time stepping and multifrontal massively parallel sparse direct solver (MUMPS) for the linear direct spatial solver.

Our COMSOL Multiphysics® simulation files are available at `https://github.com/chueschen/Toxoplasma_actin`, together with an orientation tutorial to these files and to our numerical implementation entitled COMSOLGuide.pdf.

10. Appendix

10.1. Dry vs. wet active matter: estimating frictional drag from a fixed surface

As discussed in section 6.1, the field of active matter has distinguished so-called wet and dry active matter. Qualitatively, the distinction centers on whether there is hydrodynamic coupling between the active agents through the intervening medium that they occupy. In wet active matter, fluid mediated coupling between active agents is significant. In dry active matter, these viscous forces are negligible relative to frictional drag, commonly due to the presence of a "no slip" boundary, as when filaments move over a fixed glass slide during a traditional *in vitro* gliding assay. To help assess whether the dynamics of our *Toxoplasma gondii* surface actin are in the wet or dry regime, we sought an order-of-magnitude estimate of frictional drag, for comparison to viscous forces.

For an actin filament moving in the thin ≈ 25 nm layer between the plasma membrane and the inner membrane complex, we can compute the way that the filament entrains the surrounding fluid using the driven Stokes equations with friction,

$$-\eta\nabla^2\mathbf{v} + \nabla\pi = \mathbf{F}_{myosin} - \gamma\mathbf{v}, \tag{89}$$

where the external force is composed of a driving by myosin motors, $\mathbf{F}_{myosin}$ and a frictional drag, $\gamma\mathbf{v}$, leading to a fluid velocity $\mathbf{v}$. Note that the units of all of these terms are $force/volume$. Next, we compare the relative magnitudes of the friction and viscous terms to determine if our system is indeed in the "dry" active matter regime in which

$$\gamma\mathbf{v} \gg \eta\nabla^2\mathbf{v}. \tag{90}$$

If so, friction is quantitatively much stronger than the viscous drag due to interactions between adjacent fluid elements. We can rewrite that condition as

$$\frac{\gamma}{\eta} \gg \frac{\nabla^2\mathbf{v}}{\mathbf{v}}. \tag{91}$$

To understand the source of friction in our two-dimensional system and estimate its magnitude, we must remember that our two-dimensional flow is in fact an approximation of a very thin, but three-dimensional flow as shown in Figure S12. Instead of a thin 3D fluid layer with viscous internal forces in all three directions, we have a two-dimensional flow in which internal viscous interactions across the third dimension, x_3, are captured as a friction with the fixed substrate. To estimate this friction coefficient, we approximate the viscous term in x_3 using

$$\frac{\text{force}}{\text{volume}} = \frac{\partial\sigma}{\partial x_3} \approx \frac{\sigma}{h} \approx \eta\frac{v}{h^2}. \tag{92}$$

Equating this internal shear force in x_3 with a two-dimensional frictional drag allows us to make the approximation

$$\eta\frac{v}{h^2} = \gamma v \implies \gamma = \frac{\eta}{h^2} \approx \frac{10^{-3}\,\mathrm{N\cdot s/m^2}}{(10^{-8}\ \mathrm{m})^2} \approx 10^{13}\frac{\mathrm{N\cdot s}}{\mathrm{m}^4}, \tag{93}$$

providing an estimate for the friction coefficient in terms of the known viscosity and the thickness of the fluid layer.

We can now check to see whether we are in the regime of dry active matter implied by eqn. 90. Using $10^{-3}\,\frac{\mathrm{N\cdot s}}{\mathrm{m}^2}$, the viscosity of water, as a reasonable estimate for η, and plugging in the relevant numbers, we find the scale of frictional drag to be

$$\gamma\mathbf{v} \approx \left(10^{13}\frac{\mathrm{N\cdot s}}{\mathrm{m}^4}\right)\left(10^{-6}\frac{\mathrm{m}}{\mathrm{s}}\right) \approx 10^{7}\frac{\mathrm{N}}{\mathrm{m}^3} \tag{94}$$

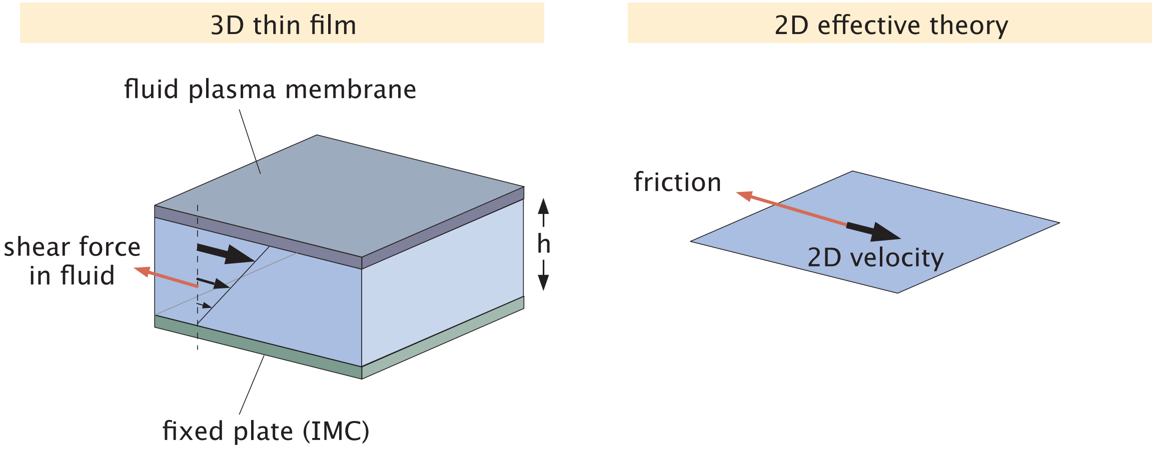

Figure S12. Effective theory of two-dimensional fluid motion. In the *Toxoplasma* cell, the actin filaments are moving in a thin layer between the plasma membrane and the inner membrane complex. That thin layer of fluid motion results in internal shear stresses. The effective theory used in our Toner-Tu description is an idealized two-dimensional fluid where the fluid coupling to the no slip boundary conditions in the 3D thin film is replaced by a friction force. Essentially, viscous stresses in the direction perpendicular to the inner membrane complex, idealized here as a fixed plate, are replaced with an in-plane friction.

and the scale of viscous forces to be

$$\eta \nabla^2 \mathbf{v} \approx \left(10^{-3} \frac{\mathrm{N} \cdot \mathrm{s}}{\mathrm{m}^2}\right) \left(\frac{10^{-6}\ \mathrm{m/s}}{(10^{-6}\ \mathrm{m})^2}\right) \approx 10^{-3} \frac{\mathrm{N}}{\mathrm{m}^3}. \tag{95}$$

Thus,

$$\gamma \mathbf{v} \gg \eta \nabla^2 \mathbf{v}, \tag{96}$$

consistent with the dry active matter limit entertained here and the choice of a Toner-Tu model.

10.2. Estimates of F-actin polymerization and depolymerization rates

To get a feeling for the polymerization (filament production) rate c, we consider actin dynamics in better-studied systems, where we can use published measurements and the BioNumbers database [62] to perform better-informed order-of-magnitude estimates.

First, we consider the comet tail behind motile *Listeria monocytogenes* bacteria as they hijack the host cell actin machinery to propel themselves at a speed that is dictated by their comet tail actin dynamics. For this estimate, we assume a modest speed of 1/10 of a body length each second, or $v \approx 200$ nm/s. We estimate that the propulsion is due to a collection of aligned actin filaments behind the bacterium with a mean spacing of ≈ 50 nm [63] resulting in a comet tail with a cross-sectional profile of $20 \times 20 = 400$ filaments, or a 1000 nm $\times$ 1000 nm area. We assume that each of these filaments is 100 nm in length. Thus, to move at a speed of ≈ 200 nm/s (2 filaments/s), the total filament polymerization rate must be

$$\text{actin production rate} \approx 2 \times 400\ \frac{\text{filaments}}{\mu\mathrm{m}^2 \cdot \mathrm{s}}. \tag{97}$$

Using this result, we make the order-of-magnitude estimate for the *Toxoplasma gondii* actin polymerization rate of

$$c \approx 10^3 \frac{\text{filaments}}{\mu\mathrm{m}^2 \cdot \mathrm{s}}, \tag{98}$$

implying a steady-state density

$$\rho_{ss} = \frac{c}{\gamma}\frac{A_{conoid}}{A_{tot}} \approx \frac{10^3\ \frac{\text{filaments}}{\mu m^2\ s}}{0.5\ \text{s}^{-1}}\frac{1}{20} \approx 100\ \frac{\text{filaments}}{\mu\text{m}^2}. \tag{99}$$

This number matches our estimated critical filament density, $\rho_c \approx 100\ \mu m^{-2}$.

Considering an actin comet tail of steady-state length $\approx$ 5 μm, we can also estimate a filament lifetime of $1/\gamma \approx 5$ μm / 0.2 μm $\text{s}^{-1} \approx 25$ s. Since actin filaments in *Listeria* comet tails are stabilized and bundled by actin-binding proteins [64], this estimate of $\gamma \approx 0.04\ \text{s}^{-1}$ likely represents a far lower bound on filament turnover rate for *Toxoplasma gondii*, where filaments are not thought to form stable bundles, and the activity of actin depolymerizing factor (ADF) is important to gliding motility [10].

For another simple estimate of depolymerization rate, we can consider actin off rates, measured *in vitro* to be $\approx$ 10 monomers/s at concentrations of 10-20 μM of *Toxoplasma gondii* actin [65]. While actin concentrations in *Toxoplasma* cells are much higher [65], lowering off rates perhaps 10-fold, we expect proteins such as ADF to increase filament turnover approximately 20-fold [66]. Thus, given a filament length of 100 nm $\approx$ 40 monomers, we estimate a rough filament lifetime for *Toxoplasma* actin of

$$\text{filament lifetime} = \frac{1}{\gamma} \approx \frac{40 \text{ monomers}}{20 \text{ monomers/s}} \approx 2 \text{ s}, \tag{100}$$

or a filament turnover rate of $\gamma \approx 0.5\ \text{s}^{-1}$, which matches our estimates for γ in Section 7.2. Considering the dynamic regulation of filament turnover by proteins like ADF, tuning γ, our final rough estimate for a reasonable range for *Toxoplasma gondii* filament lifetime is between 0.2 s and 10 s, which is equivalent to a range of filament turnover rate γ between 0.1 filaments/s and 5 filaments/s. We note that cells may actively tune depolymerization rate over an order of magnitude through the activity of proteins like ADF [66].

10.3. Estimates of resistive forces that could perturb actin 'herding' during Toxoplasma gondii gliding

As discussed in Section 6.5, the Toner-Tu model of *Toxoplasma gondii* actin self-organization presented in this work explores the collective dynamics of motor-propelled actin filaments without reference to the external environment and potential resistive force. Is this focus on the myosin-driven actin system itself (internal dynamics) justified?

In this Appendix, we present several highly approximate estimates to gain insight into the magnitude of forces that resist the movement of myosin-drive actin filaments below the plasma membrane in gliding *Toxoplasma gondii* cells. Our calculations below indicate that on the entirety of the cell including the point of substrate contact the dynamics of actin filaments are dominated by a single player (pN-scale active forces from myosin motors), while each resistive force we considered was found to be at least an order-of-magnitude smaller. These resistive forces included drag force on the cell as it moves through fluid, including effects from the no-slip substrate boundary, as well as the drag on actin-coupled adhesin proteins moving through membrane. In short, these estimates suggest that an internal model of actin self-organization is reasonable and useful.

10.3.1. Overcoming the drag due to cell motion.

First, we remind the reader that when gliding on a substrate, the *Toxoplasma* cell moves over a point of contact/adhesion. In the laboratory reference frame, that point of contact is stationary. In the cell reference frame, it is coupled to and follows the flow of actin filaments, as shown in Figure S7.

In this sub-section and the next, our key goal is to determine whether the myosin motors at the point of substrate contact/adhesion (the ones carrying the cell on their backs, so to speak), are under enough load to slow them down. If the load of pulling the cell through fluid slowed those motors down, the actin

filaments at the point of substrate contact/adhesion would move more slowly, introducing heterogeneity into the system that was not explored in our model.

Thus, we ask how large a drag force the cell encounters as a result of its motion through the surrounding medium. This same kind of question arises in thinking about the fluid drag on the micron-scale polystyrene beads that are used in optical trapping when measuring the force-velocity properties of molecular motors. In that case, a first guess might be that the bead which is huge in comparison with the motor itself might produce significant fluid drag that would impact the motor dynamics. In fact, using reasoning identical to that presented below, the drag forces are in the range of tens-to-hundreds of femptonewtons, while the force scale that perturbs motor velocities are of order piconewtons.

To get a sense of the force scale associated with fluid drag on a *Toxoplasma*-sized object, we consider the drag force on a sphere of radius $a = 1$ μm moving at a fast gliding speed of $v = 5$ μm/s. We argue that the distinction between a sphere and the elongated shape of the apicomplexan is not relevant for our order-of-magnitude estimate. Using the Stokes drag, the drag force is given by

$$F_{drag} = 6\pi\eta a v, \tag{101}$$

resulting in the estimate

$$F_{drag} \approx 6 \times \pi \times 10^{-3} \text{ N s/m}^2 \times 5 \times 10^{-6} \text{ m} \times 10^{-6} \text{ m/s} \approx 0.1 \text{ pN}. \tag{102}$$

Measured force-velocity relationships for myosins show that they slow with pN-scale loads (e.g., Norstrom et al., JBC 2010). Thus, a drag force with a magnitude of 0.1 pN is unlikely to significantly change motor velocity, suggesting that external fluid resistance will have little impact on internal actin dynamics.

10.3.2. Shear forces at the cell-substrate interface.

Another source of resistance to cellular motion might come from the proximity of the cell and the glass coverslip over which it moves. In particular, we consider the shear stresses that must arise because of the no-slip condition at the coverslip, with the nearby cell moving at a speed of a few microns per second. In this case, we compute the shear stress using

$$\sigma_{xz} = \eta \frac{\partial v_x}{\partial z}. \tag{103}$$

As a simple estimate, we consider the approximation

$$\frac{\partial v_x}{\partial z} \approx \frac{v_{cell}}{h} \tag{104}$$

where v_{cell} is the speed of the cell and h is the distance of the cell from the cover slip. To get a sense of the scale of this shear stress, we assume that the distance h of the cell from the coverslip is of molecular dimensions since the linkage between the cell and the substrate is mediated by adhesion proteins, and we use a fast gliding speed of 5 μm/s. Thus, we find

$$\sigma_{xz} \approx 10^{-3} \text{ Pa s } \frac{5 \times 10^{-6} \text{ m/s}}{10 \times 10^{-9} \text{ m}} \approx \frac{1}{2} \text{ N/m}^2. \tag{105}$$

This shear stress acts over the area of the cell closest to the substrate. To be concrete, we imagine this near-boundary cell area as being 0.5 μm ×0.5 μm, resulting in the estimate

$$F_{surface} \approx \frac{1}{2} \text{ N/m}^2 \times \frac{1}{2} \times 10^{-6} \text{ m} \times \frac{1}{2} \times 10^{-6} \text{ m} \approx 0.1 \text{ pN}. \tag{106}$$

Our estimate likely errs on the large side, as we suspect that h is larger than 10 nm over much of that 0.5 μm ×0.5 μm cell area. Even so, we find again that the force scale resulting from this drag mechanism is an order of magnitude smaller than the stall force of myosin motors. Here too, we conclude that the drag due to shear stresses at the cell-coverslip interface will have a negligible effect on the actin dynamics.

10.3.3. Drag force due to adhesion protein motion through the cell membrane.

Actin filaments at the surface of the *Toxoplasma* cell bind to transmembrane proteins called adhesins. A final possible mechanism of drag imposed on the actin movements and organization is the drag on actin-coupled adhesin proteins moving through the plasma membrane. As a first approximation, we assume that the density of adhesin proteins in the plasma membrane is similar to the density of myosin motors in the IMC. Given the continuous turnover and cleavage [67] of adhesin proteins in the plasma membrane, we suspect that adhesin density may be lower, but as an upper bound we assume a 1:1 ratio of adhesins to motors and seek to compare the drag on one adhesin protein to the stall force of a single myosin. One way to estimate the drag force makes use of measured diffusion coefficients for proteins in the cell membrane. The fluctuation-dissipation theorem relates the diffusion constant D to the drag coefficient γ through the relation

$$D = \frac{k_B T}{\gamma}. \tag{107}$$

This equation presents our first strategy for estimating the drag force by evaluating the drag coefficient as

$$\gamma = \frac{k_B T}{D} \tag{108}$$

Given that measured values for the diffusion coefficient of proteins in the plasma membrane are on the order of 0.1 $\mu m^2/s$ [68] (*i.e.* 1×10^5 nm^2/s) we estimate the drag coefficient as

$$\gamma = \frac{4 \text{ pN} \cdot \text{nm}}{1 \times 10^5 \text{ nm}^2/\text{s}} \approx 4 \times 10^{-5} \text{ pN/(nm/s)}, \tag{109}$$

resulting in a drag force estimate of

$$F_{drag} = \gamma v \approx 4 \times 10^{-5} \text{ pN/(nm/s)} \times 5 \times 10^3 \text{ nm/s} \approx 0.2 \text{ pN}. \tag{110}$$

Yet again, we see that the force scale is smaller than forces that have been measured to reduce motor speed. We also note that the plasma membrane may flow locally with adhesins, further reducing this drag. Altogether, our estimate suggests that although the myosin motors have to pull actin filaments that are in turn attached to adhesin molecules, the resistive force offered by this pulling will not alter the “preferred speed” that appears in the Toner-Tu theory used to describe the collective F-actin motion.

References

1. K. Frénal, V. Polonais, J. B. Marq, R. Stratmann, J. Limenitakis, D. Soldati-Favre, Functional dissection of the apicomplexan glideosome molecular architecture, Cell Host and Microbe 8 (2010) 343–357.

2. A. Graindorge, K. Frénal, D. Jacot, J. Salamun, J. B. Marq, D. Soldati-Favre, The Conoid Associated Motor MyoH Is Indispensable for *Toxoplasma gondii* Entry and Exit from Host Cells, PLoS Pathogens 12 (2016) 1–26.

3. L. W. Bergman, K. Kaiser, H. Fujioka, I. Coppens, T. M. Daly, S. Fox, K. Matuschewski, V. Nussenweig, S. H. Kappe, Myosin A tail domain interacting protein (MTIP) localizes to the inner membrane complex of *Plasmodium* sporozoites, Journal of Cell Science 116 (2003) 39–49.

4. K. Frénal, J. F. Dubremetz, M. Lebrun, D. Soldati-Favre, Gliding motility powers invasion and egress in Apicomplexa, Nature Reviews Microbiology 15 (2017) 645–660.

5. R. V. Stadler, L. A. White, K. Hu, B. P. Helmke, W. H. Guilford, Direct measurement of cortical force generation and polarization in a living parasite, Molecular Biology of the Cell 28 (2017) 1912–1923.

6. K. Jaqaman, D. Loerke, M. Mettlen, H. Kuwata, S. Grinstein, S. L. Schmid, G. Danuser, Robust single-particle tracking in live-cell time-lapse sequences, Nature Methods 5 (2008) 695–702.

7. C. M. Miller, E. Korkmazhan, A. R. Dunn, Extraction of accurate cytoskeletal actin velocity distributions from noisy measurements, Nature Communications 13 (2022) 4749.

8. C. L. Hueschen, A. R. Dunn, R. Phillips, Wildebeest herds on rolling hills: Flocking on arbitrary curved surfaces, Physical Review E 108 (2023) 24610.

9. D. Wetzel, S. Håkansson, K. Hu, D. Roos, L. Sibley, Actin Filament Polymerization Regulates Gliding Motility by Apicomplexan Parasites, Molecular Biology of the Cell 14 (2003) 396–406.

10. S. Mehta, L. D. Sibley, Actin depolymerizing factor controls actin turnover and gliding motility in *Toxoplasma gondii*, Molecular Biology of the Cell 22 (2011) 1290–1299.

11. R. G. Douglas, P. Nandekar, J. E. Aktories, H. Kumar, R. Weber, J. M. Sattler, M. Singer, S. Lepper, S. K. Sadiq, R. C. Wade, F. Frischknecht, Inter-subunit interactions drive divergent dynamics in mammalian and *Plasmodium* actin filaments, PLoS Biology 16 (2018) 1–30.

12. M. Ganter, H. Schüler, K. Matuschewski, Vital role for the *Plasmodium* actin capping protein (CP) beta-subunit in motility of malaria sporozoites, Molecular Microbiology 74 (2009) 1356–1367.

13. M. Martinez, S. K. Mageswaran, A. Guérin, W. D. Chen, C. P. Thompson, S. Chavin, D. Soldati-Favre, B. Striepen, Y. W. Chang, Origin and arrangement of actin filaments for gliding motility in apicomplexan parasites revealed by cryo-electron tomography, Nature Communications 14 (2023) 4800.

14. M. Galassi, GNU Scientific Library: Reference Manual, GNU manual, Network Theory, 2009.

15. S. Håkansson, H. Morisaki, J. Heuser, L. D. Sibley, Time-lapse video microscopy of gliding motility in *Toxoplasma gondii* reveals a novel, biphasic mechanism of cell locomotion., Molecular biology of the cell 10 (1999) 3539–47.

16. K. L. Carey, N. J. Westwood, T. J. Mitchison, G. E. Ward, A small-molecule approach to studying invasive mechanisms of *Toxoplasma gondii*, Proceedings of the National Academy of Sciences of the United States of America 101 (2004) 7433–7438.

17. A. Farrell, S. Thirugnanam, A. Lorestani, J. D. Dvorin, K. P. Eidell, D. J. Ferguson, B. R. Anderson-White, M. T. Duraisingh, G. T. Marth, M. J. Gubbels, A DOC2 protein identified by mutational profiling is essential for apicomplexan parasite exocytosis, Science 335 (2012) 218–221.

18. K. S. Bane, S. Lepper, J. Kehrer, J. M. Sattler, M. Singer, M. Reinig, D. Klug, K. Heiss, J. Baum, A. K. Mueller, F. Frischknecht, The Actin Filament-Binding Protein Coronin Regulates Motility in *Plasmodium* Sporozoites, PLoS Pathogens 12 (2016) 1–26.

19. S. Münter, B. Sabass, C. Selhuber-Unkel, M. Kudryashev, S. Hegge, U. Engel, J. P. Spatz, K. Matuschewski, U. S. Schwarz, F. Frischknecht, *Plasmodium* Sporozoite Motility Is Modulated by the Turnover of Discrete Adhesion Sites, Cell Host and Microbe 6 (2009) 551–562.

20. J. Salamun, J. P. Kallio, W. Daher, D. Soldati-Favre, I. Kursula, Structure of *Toxoplasma gondii* coronin, an actin-binding protein that relocalizes to the posterior pole of invasive parasites and contributes to invasion and egress, FASEB Journal 28 (2014) 4729–4747.

21. D. Jacot, N. Tosetti, I. Pires, J. Stock, A. Graindorge, Y. F. Hung, H. Han, R. Tewari, I. Kursula, D. Soldati-Favre, An Apicomplexan Actin-Binding Protein Serves as a Connector and Lipid Sensor to Coordinate Motility and Invasion, Cell Host and Microbe 20 (2016) 731–743.

22. S. Kappe, T. Bruderer, S. Gantt, H. Fujioka, V. Nussenzweig, R. Ménard, Conservation of a gliding motility and cell invasion machinery in Apicomplexan parasites., The Journal of Cell Biology 147 (1999) 937–44.

23. M. W. Panas, A. Naor, A. M. Cygan, J. C. Boothroyd, *Toxoplasma* controls host cyclin E expression through the use of a novel myr1-dependent effector protein, HCE1, mBio 10 (2019).

24. B. A. Fox, J. G. Ristuccia, J. P. Gigley, D. J. Bzik, Efficient gene replacements in *Toxoplasma gondii* strains deficient for nonhomologous end joining, Eukaryotic Cell 8 (2009) 520–529.

25. N. D. Marino, M. W. Panas, M. Franco, T. C. Theisen, A. Naor, S. Rastogi, K. R. Buchholz, H. A. Lorenzi, J. C. Boothroyd, Identification of a novel protein complex essential for effector translocation across the parasitophorous vacuole membrane of *Toxoplasma gondii*, PLoS Pathogens 14 (2018) 1–26.

26. J. B. Grimm, B. P. English, J. Chen, J. P. Slaughter, Z. Zhang, A. Revyakin, R. Patel, J. J. Macklin, D. Normanno, R. H. Singer, T. Lionnet, L. D. Lavis, A general method to improve fluorophores for live-cell and single-molecule microscopy, Nature Methods 12 (2015) 244–250.

27. A. D. Edelstein, M. A. Tsuchida, N. Amodaj, H. Pinkard, R. D. Vale, N. Stuurman, Advanced methods of microscope control using μManager software, Journal of Biological Methods 1 (2014) e10.

28. M. Morimatsu, A. H. Mekhdjian, A. C. Chang, S. J. Tan, A. R. Dunn, Visualizing the Interior Architecture of Focal Adhesions with High-Resolution Traction Maps, Nano Letters 15 (2015) 2220–2228.

29. M. K. Shaw, L. G. Tilney, Induction of an acrosomal process in *Toxoplasma gondii*: Visualization of actin filaments in a protozoan parasite, Proceedings of the National Academy of Sciences 96 (1999) 9095–9099.

30. J. Huff, The Airyscan detector from ZEISS: confocal imaging with improved signal-to-noise ratio and super-resolution, Nature Methods 12 (2015) i–ii.

31. D. Y. Parkinson, C. Knoechel, C. Yang, C. A. Larabell, M. A. Le Gros, Automatic alignment and reconstruction of images for soft X-ray tomography, Journal of Structural Biology 177 (2012) 259–266.

32. J. H. Chen, B. Vanslembrouck, V. Loconte, A. Ekman, M. Cortese, R. Bartenschlager, G. McDermott, C. A. Larabell, M. A. Le Gros, V. Weinhardt, A protocol for full-rotation soft X-ray tomography of single cells, STAR Protocols 3 (2022) 101176.

33. F. P. Cordelières, Manual Tracking, a plug-in for ImageJ software, https://imagej.nih.gov/ij/plugins/track/track.html, 2005.

34. J. Schindelin, I. Arganda-Carreras, E. Frise, V. Kaynig, M. Longair, T. Pietzsch, S. Preibisch, C. Rueden, S. Saalfeld, B. Schmid, J. Y. Tinevez, D. J. White, V. Hartenstein, K. Eliceiri, P. Tomancak, A. Cardona, Fiji: An open-source platform for biological-image analysis, Nature Methods 9 (2012) 676–682.

35. A. Krull, T. O. Buchholz, F. Jug, Noise2void-Learning denoising from single noisy images, Proceedings of the IEEE Computer Society Conference on Computer Vision and Pattern Recognition 2019-June (2019) 2124–2132.

36. T. Vicsek, A. Czirók, E. Ben-Jacob, I. Cohen, O. Shochet, Novel Type of Phase Transition in a System of Self-Driven Particles, Physical Review Letters 75 (1995) 1226–1229.

37. J. Toner, Y. Tu, Long-range order in a two-dimensional dynamical XY model: How birds fly together, Physical Review Letters 75 (1995) 4326–4329.

38. J. Toner, Y. Tu, Flocks, herds, and schools: A quantitative theory of flocking, Physical Review E 58 (1998) 4828–4858.

39. M. C. Marchetti, J. F. Joanny, S. Ramaswamy, T. B. Liverpool, J. Prost, M. Rao, R. A. Simha, Hydrodynamics of soft active matter, Reviews of Modern Physics 85 (2013) 1143–1189.

40. A. Herm-Götz, S. Weiss, R. Stratmann, S. Fujita-Becker, C. Ruff, E. Meyhöfer, T. Soldati, D. J. Manstein, M. A. Geeves, D. Soldati, *Toxoplasma gondii* myosin A and its light chain: A fast, single-headed, plus-end-directed motor, EMBO Journal 21 (2002) 2149–2158.

41. M. A. Olshina, F. Angrisano, D. S. Marapana, D. T. Riglar, K. Bane, W. Wong, B. Catimel, M. X. Yin, A. B. Holmes, F. Frischknecht, D. R. Kovar, J. Baum, *Plasmodium falciparum* coronin organizes arrays of parallel actin filaments potentially guiding directional motility in invasive malaria parasites, Malaria Journal 14 (2015) 1–18.

42. A. Sciortino, A. R. Bausch, Pattern formation and polarity sorting of driven actin filaments on lipid membranes, Proceedings of the National Academy of Sciences 118 (2021) 1–8.

43. R. Phillips, J. Kondev, J. Theriot, H. G. Garcia, Physical biology of the cell, 2nd Edition, Garland Science, New York, 2013. (Illustrated by N. Orme).

44. P. VanBuren, W. H. Guilford, G. Kennedy, J. Wu, D. M. Warshaw, S. Chako, S. Block, Smooth muscle myosin: A high force-generating molecular motor, Biophysical Journal 68 (1995) 256–258.

45. F. G. Woodhouse, R. E. Goldstein, Cytoplasmic streaming in plant cells emerges naturally by microfilament self-organization, Proceedings of the National Academy of Sciences of the United States of America 110 (2013) 14132–14137.

46. M. Kardar, Statistical Physics of Fields, Cambridge University Press, 2007.

47. J. Howard, Mechanics of motor proteins and the cytoskeleton, Sinauer Associates, 2001.

48. F. Morgan, Riemannian Geometry, A K Peters/CRC Press, 1998.

49. J. Baum, C. J. Tonkin, A. S. Paul, M. Rug, B. J. Smith, S. B. Gould, D. Richard, T. D. Pollard, A. F. Cowman, A Malaria Parasite Formin Regulates Actin Polymerization and Localizes to the Parasite-Erythrocyte Moving Junction during Invasion, Cell Host and Microbe 3 (2008) 188–198.

50. F. Plattner, F. Yarovinsky, S. Romero, D. Didry, M. F. Carlier, A. Sher, D. Soldati-Favre, *Toxoplasma* Profilin Is Essential for Host Cell Invasion and TLR11-Dependent Induction of an Interleukin-12 Response, Cell Host and Microbe 3 (2008) 77–87.

51. S. Schmitz, M. Grainger, S. Howell, L. J. Calder, M. Gaeb, J. C. Pinder, A. A. Holder, C. Veigel, Malaria parasite actin filaments are very short, Journal of Molecular Biology 349 (2005) 113–125.

52. N. Sahoo, W. Beatty, J. Heuser, D. Sept, L. D. Sibley, Unusual kinetic and structural properties control rapid assembly and turnover of actin in the parasite *Toxoplasma gondii.*, Molecular Biology of the Cell 17 (2006) 895–906.

53. T. Jankuhn, M. A. Olshanskii, A. Reusken, Incompressible fluid problems on embedded surfaces: Modeling and variational formulations, Interfaces and Free Boundaries 20 (2018) 353–378.

54. COMSOL Multiphysics® v. 5.5. www.comsol.com. COMSOL AB, Stockholm, Sweden., 2019.

55. M. Styner, I. Oguz, S. Xu, C. Brechbühler, D. Pantazis, J. Levitt, M. Shenton, G. Gerig, Framework for the Statistical Shape Analysis of Brain Structures using SPHARM-PDM., Insight J. 1071 (2006) 242–250.

56. B. Paniagua, O. Emodi, J. Hill, J. Fishbaugh, L. A. Pimenta, S. R. Aylward, E. Andinet, G. Gerig, J. Gilmore, J. A. van Aalst, M. Styner, 3D of brain shape and volume after cranial vault remodeling surgery for craniosynostosis correction in infants, Medical Imaging 2013: Biomedical Applications in Molecular, Structural, and Functional Imaging 8672 (2013) 86720V.

57. C. Brechbüler, G. Gerig, O. Kübler, Parametrization of Closed Surfaces for 3-D Shape Description, Computer Vision and Image Understanding 61 (1995) 154–170.

58. T. D. Goddard, C. C. Huang, E. C. Meng, E. F. Pettersen, G. S. Couch, J. H. Morris, T. E. Ferrin, UCSF ChimeraX: Meeting modern challenges in visualization and analysis, Protein Science 27 (2018) 14–25.

59. A. Fedorov, R. Beichel, J. Kalpathy-Cramer, J. Finet, J.-C. Fillion-Robin, S. Pujol, C. Bauer, D. Jennings, F. Fennessy, M. Sonka, J. Buatti, S. Aylward, J. V. Miller, S. Pieper, R. Kikinis, 3D Slicer as an image computing platform for the Quantitative Imaging Network, Magnetic Resonance Imaging 30 (2012) 1323–1341.

60. J. Vicory, L. Pascal, P. Hernandez, J. Fishbaugh, J. Prieto, M. Mostapha, C. Huang, H. Shah, J. Hong, Z. Liu, L. Michoud, J.-C. Fillion-Robin, G. Gerig, H. Zhu, S. M. Pizer, M. Styner, B. Paniagua, SlicerSALT: Shape AnaLysis Toolbox, in: Shape in Medical Imaging, 2018, 2018, pp. 65–72.

61. COMSOL Multiphysics, The Finite Element Method (FEM), Multiphysics Cyclopedia, https://www.comsol.com/multiphysics/finite-element-method, 2017.

62. R. Milo, P. Jorgensen, U. Moran, G. Weber, M. Springer, BioNumbers The database of key numbers in molecular and cell biology, Nucleic Acids Research 38 (2009) 750–753.

63. L. A. Cameron, T. M. Svitkina, D. Vignjevic, J. A. Theriot, G. G. Borisy, Dendritic organization of actin comet tails, Current Biology 11 (2001) 130–135.

64. M. Van Troys, A. Lambrechts, V. David, H. Demol, M. Puype, J. Pizarro-Cerda, K. Gevaert, P. Cossart, J. Vandekerckhove, The actin propulsive machinery: the proteome of *Listeria monocytogenes* tails., Biochemical and biophysical research communications 375 (2008) 194–199.

65. K. L. Hvorecny, T. E. Sladewski, E. M. De La Cruz, J. M. Kollman, A. T. Heaslip, Toxoplasma gondii actin filaments are tuned for rapid disassembly and turnover, Nature Communications 15 (2024) 1–16.

66. M. F. Carlier, V. Laurent, J. Santolini, R. Melki, D. Didry, G. X. Xia, Y. Hong, N. H. Chua, D. Pantaloni, Actin depolymerizing factor (ADF/cofilin) enhances the rate of filament turnover: Implication in actin-based motility, Journal of Cell Biology 136 (1997) 1307–1322.

67. V. B. Carruthers, G. D. Sherman, L. D. Sibley, The Toxoplasma adhesive protein MIC2 is proteolytically processed at multiple sites by two parasite-derived proteases, Journal of Biological Chemistry 275 (2000) 14346–14353.

68. A. Kusumi, T. K. Fujiwara, R. Chadda, M. Xie, T. A. Tsunoyama, Z. Kalay, R. S. Kasai, K. G. Suzuki, Dynamic organizing principles of the plasma membrane that regulate signal transduction: Commemorating the fortieth anniversary of singer and nicolson's fluid-mosaic model, Annual Review of Cell and Developmental Biology 28 (2012) 215–250.

COMSOL Multiphysics® Simulations Guide

Emergent Actin Flows Explain Diverse Parasite Gliding Modes

Christina L. Hueschen, Li-av Segev Zarko, Jian-Hua Chen, Mark A. LeGros, Carolyn A. Larabell, John C. Boothroyd, Rob Phillips, Alexander R. Dunn

We hope that others will find the numerical approach used here and in Hueschen, Dunn, Phillips 2023 [8] to be user-friendly and adaptable for solving the Toner-Tu equations on other complex curved surfaces or for solving other continuum equations on curved surfaces. Our COMSOL Multiphysics® files are available at `https://github.com/chueschen/Toxoplasma_actin`. Related files and a more thorough tutorial introduction to these simulations are found at `https://github.com/RPGroup-PBoC/wildebeest_herds`.

While we chose COMSOL Multiphysics® for its accessibility and learner-friendly interface, we note with regret that the use of these files requires access to a paid COMSOL Multiphysics® license. If you do not already have a license and are affiliated with an institution, we recommend looking into access options through a shared software library.

Files available in this repository:

1. Hueschen_ToxoActinOrganization_StableActin. Actin flocking model predicts self-organized recirculation ("cyclosis") of actin patches in the absence of filament turnover. The simulation begins with a disordered network, and then filament density ρ and velocity $\mathbf{v}$ evolve over time on the *Toxoplasma gondii* tachyzoite cell surface according the actin self-organization Toner-Tu equations presented in Supplementary Info Sections 6 - 9. Filaments are stable and conserved, as in Supplementary Figure S3 A-B. This simulation produced the results shown in Figure 3B and in Video 4.

2. Hueschen_ToxoActinOrganization_ActinTurnover. Actin flocking model predicts the emergence of self-organized unidirectional flow in the presence of filament turnover. The simulation begins with a disordered network, and then filament density ρ and velocity $\mathbf{v}$ evolve over time on the *Toxoplasma gondii* tachyzoite cell surface according the actin self-organization Toner-Tu equations presented in Supplementary Info Sections 6-9. Filaments are polymerized in the conoid (anterior end) at a rate per c $\mu m^{-2}s^{-1}$ and depolymerized throughout the cell surface at rate $\gamma\rho$ $\mu m^{-2}s^{-1}$, as in Supplementary Figure S3C-D and Section 7.2. This simulation produced the results shown in Figure 4 and Video 6.

A note of caution: This document, together with the How-To Guide found at `https://github.com/RPGroup-PBoC/wildebeest_herds`, are intended to provide a quick, practical orientation to our working files and to the use of the COMSOL Multiphysics® interface for solving PDEs. For those new to the world of finite elements, we recommend seeking out general training from local experts or written resources on both principles of the finite element method and practical tips: avoiding common pitfalls; carefully choosing a solver, mesh size, time step size; verifying against analytical results, etc. COMSOL's own thorough introduction to the finite element method is available at `https://www.comsol.com/multiphysics/finite-element-method`.

Quick-start tutorial:

To solve our custom surface partial differential equations, we used the COMSOL Multiphysics® General Form Boundary PDE interface and took advantage of COMSOL's built-in tangential differentiation operator, dtang(f,x). The heart of our implementation of a general curved-surface formulation of the Toner-Tu equations is described in Section 8 ('Deriving a Tangential Formulation of the Filament Self-Organization Equations') in the supplementary information. Here, in the form of an abridged tutorial,

we provide a practical guide to using that implementation and the COMSOL Multiphysics® interface. A more complete version is available at `https://github.com/RPGroup-PBoC/wildebeest_herds`.

1. Parameters, Geometry, and Meshing. In COMSOL Multiphysics®, open the file 'Hueschen_ToxoActinOrganization_StableActin.mph,' which explores F-actin self-organization in the absence of filament turnover. Begin to orient yourself to the side panel on the left. Under 'Global Definitions,' you'll find our **parameters** (e.g., Toner-Tu coefficients and initial conditions). Now, under 'Component 1,' expand 'Geometry 1'. Here, we could use simple geometric features to build a geometry or import one, as was done in our case for the *Toxoplasma* cell shape extracted from soft X-ray tomograms.

2. Variables and PDEs.

First, click on Variables 1 under Component 1 / Definitions. Here, weve defined important pieces of the curved-surface formulation, such as the projection operator **P** and the surface velocity gradient tensor **G**. Try to familiarize yourself with these quantities, referencing Supplementary Information Section 8.

Under 'Component 1,' click on the first General Form Boundary PDE (velocity). This is our implementation of the Toner-Tu-based self-organization equations for velocity. Note the sections on Units, Discretization, and Dependent variables, which allow us to set the **field variables** for which we wish to solve (in this case, v_1, v_2, and v_3) and, importantly, choose the element order and type of shape function we'll use to describe our velocity field. Next, expand 'General Form Boundary PDE (velocity)' and click on 'General Form PDE 1.' Now we're really getting to the heart of things! Expand the Equation section to see the PDE form used here. Referencing Section 8 in the SI, notice how the contents of $\mathbf{\Gamma}$, f, and d_a combine to **implement our curved-space self-organization velocity equations**, Eqn. 84. Syntax tip: $v1x$ is the derivative of v_1 with respect to x, and $rhox$ is the derivative of ρ with respect to x. Note that we are now working in 3D and have defined three components of our velocity vector, but we're using a 'boundary' PDE module to solve our equations on the surface of our sphere. To practice adding your own Boundary PDE module, right-click on 'Component 1' and choose 'Add Physics / Mathematics / PDE Interfaces / Lower Dimensions / General Form Boundary PDE.'

Click on the second General Form Boundary PDE (continuity). Here we have defined a field of the scalar dependent variable density, ρ, and described it with a linear shape function. Within 'General Form PDE 1,' you'll find our implementation of the curved-space **continuity equation** presented in Eqn. 86. In this example of stabilized actin, the source and sink terms (c and γ) are not present.

Next, click on 'Boundary ODEs and DAEs.' Here, we are using an ODE simply to create a field of normal vectors (n_1, n_2, n_3) that is defined between mesh nodes using a specific shape function. (Our thanks to Yue Huang of COMSOL technical support for suggesting this trick.) Note that the normal vector components nx, ny, and nz are built-in COMSOL geometric variables.

Finally, notice a couple of constraints specific to the curved-surface implementation. These constraints are called for by the imperfect world of numerics, not by the mathematics, and prevent small numerical errors from expanding over time. Within the velocity PDE module, 'Weak Constraint 1' helps to enforce the condition that the velocity vector is nonzero only in the surface tangent plane ($\mathbf{n} \cdot \mathbf{v} = 0$), using terminology defined in 'Variables 1.' Within 'General Form Boundary PDE - continuity,' notice the 'Global Constraint 1' that enforces global conservation of mass. The global integration function 'intop1' is defined within 'Component 1 / Definitions / Integration 1,' and the global integrated density (total number of filaments) calculated using that function is defined in 'Parameters 1.'

3. Initial and Boundary Conditions. Look back at the General Form PDE for velocity and click on 'Initial Values 1'. To **initialize with a disordered velocity field**, we are drawing every node's velocity orientation randomly from a uniform distribution of angles between 0 and 2π, and scaling that by magnitude v_0. Our random angle is generated by the function $rn1(x, y)$, which is defined under 'Global Definitions / Random 1.' The magnitude v_0 was defined under 'Global Definitions / Parameters 1.' Our **initial condition** for our General Form Boundary PDE for density (continuity) is a uniform density field of magnitude ρ_0, which is similarly defined in 'Parameters 1.'

4. Solving. Under 'Study 1', you'll find a **Time Dependent solver** module. Click on 'Step 1: Time Dependent' to customize the time range of the simulation. We used default COMSOL solvers and settings: implicit backward differentiation formula (BDF) for time stepping and multifrontal massively parallel sparse direct solver (MUMPS) for the linear direct spatial solver. Note the Time Stepping settings under 'Solver Configurations / Solution 1 / Time-Dependent Solver 1.'

Now, another big moment! Hit 'Compute' to run the simulation. If you're in a rush, shorten the time range of the simulation. Note: at the end of step 5, we'll discuss **parametric sweeps**.

5. Visualizing and Exporting Results. Move to the Results section on the side panel to **visualize our simulation**. As an example, expand 'Density + velocity arrows.' Note how 'Surface 1' sets up a color table based on density and how 'Arrow Surface 1' displays velocity with a field of customizable arrows. To create your own surface plot, try right-clicking on 'Results' and choose '3D Plot Group.'

Under 'Export,' use 'density velocity file export' to practice **exporting a .gif movie** of the velocity plot discussed above. Undersampling by selecting a limited number of frames or a limited time window may be useful to reduce file size. Then, notice how 'density velocity player' is set up to play within COMSOL. To add your own animations, right-click on 'Export' and find 'Animation.'

Bonus: To re-run the simulation while **sweeping through different parameter choices**, right-click on 'Study 1' and choose 'Parametric Sweep.' Click on the '+' icon and add a short list of choices for a parameter of interest. After computing, update your 'Results' plots to visualize this new dataset (Study 1/Parametric Solutions 1).
