# Supplementary material for: Emergent actin flows explain distinct modes of gliding motility
Source: Nat Phys. 2024 Oct 8;20(12):1989–96. doi: 10.1038/s41567-024-02652-4 (PMC11631758; doi:10.1038/s41567-024-02652-4)
Supplement: Supplementary file 2 — Reporting Summary [file 41567_2024_2652_MOESM2_ESM.pdf]

nature portfolio

Corresponding author(s): Alexander R. Dunn, Christina L. Hueschen

Last updated by author(s): 07/12/2024

Reporting Summary

Nature Portfolio wishes to improve the reproducibility of the work that we publish. This form provides structure for consistency and transparency in reporting. For further information on Nature Portfolio policies, see our Editorial Policies and the Editorial Policy Checklist.

Statistics

For all statistical analyses, confirm that the following items are present in the figure legend, table legend, main text, or Methods section.

n/a	Confirmed	
☐	☒	The exact sample size (n) for each experimental group/condition, given as a discrete number and unit of measurement
☐	☒	A statement on whether measurements were taken from distinct samples or whether the same sample was measured repeatedly
☐	☒	The statistical test(s) used AND whether they are one- or two-sided<br>*Only common tests should be described solely by name; describe more complex techniques in the Methods section.*
☒	☐	A description of all covariates tested
☐	☒	A description of any assumptions or corrections, such as tests of normality and adjustment for multiple comparisons
☐	☒	A full description of the statistical parameters including central tendency (e.g. means) or other basic estimates (e.g. regression coefficient) AND variation (e.g. standard deviation) or associated estimates of uncertainty (e.g. confidence intervals)
☐	☒	For null hypothesis testing, the test statistic (e.g. F, t, r) with confidence intervals, effect sizes, degrees of freedom and P value noted<br>*Give P values as exact values whenever suitable.*
☒	☐	For Bayesian analysis, information on the choice of priors and Markov chain Monte Carlo settings
☒	☐	For hierarchical and complex designs, identification of the appropriate level for tests and full reporting of outcomes
☒	☐	Estimates of effect sizes (e.g. Cohen's d, Pearson's r), indicating how they were calculated

Our web collection on statistics for biologists contains articles on many of the points above.

Software and code

Policy information about availability of computer code

Data collection	Micromanager version 1.4 software, ChimeraX version 1.0 software, and Zen 2.3 (black edition) software.
Data analysis	FIJI/ImageJ (Version 2.0.0-rc-69/1.52p), the ImageJ Manual Tracking plugin, u-track software (Danuser lab, release 2.2.0), and home-written custom MatLab code (Mathworks, version 2020a) available at https://github.com/chueschen/Toxoplasma_actin were used during data analysis.

For manuscripts utilizing custom algorithms or software that are central to the research but not yet described in published literature, software must be made available to editors and reviewers. We strongly encourage code deposition in a community repository (e.g. GitHub). See the Nature Portfolio guidelines for submitting code & software for further information.

Data

Policy information about availability of data

All manuscripts must include a data availability statement. This statement should provide the following information, where applicable:

- Accession codes, unique identifiers, or web links for publicly available datasets
- A description of any restrictions on data availability
- For clinical datasets or third party data, please ensure that the statement adheres to our policy

Plasmids, Toxoplasma gondii strains, and microscopy movies are available upon request. The BioNumbers database referenced in the Supplemental Information is available at https://bionumbers.hms.harvard.edu/search.aspx.

Human research participants

Policy information about studies involving human research participants and Sex and Gender in Research.

Reporting on sex and gender	N/A
Population characteristics	N/A
Recruitment	N/A
Ethics oversight	N/A

Note that full information on the approval of the study protocol must also be provided in the manuscript.

Field-specific reporting

Please select the one below that is the best fit for your research. If you are not sure, read the appropriate sections before making your selection.

☒ Life sciences ☐ Behavioural & social sciences ☐ Ecological, evolutionary & environmental sciences

For a reference copy of the document with all sections, see nature.com/documents/nr-reporting-summary-flat.pdf

Life sciences study design

All studies must disclose on these points even when the disclosure is negative.

Sample size	Sample sizes were not determined a priori. For each experimental condition/replicate, we checked the robustness of our measurements by cell-to-cell and experiment-to-experiment comparisons and using statistical tests, when appropriate. More on all experiments: Single-molecule velocity measurements in Fig. S2 were used to report that a larger fraction of actin molecules were mobile compared to myosin molecules. With the sample sizes used, this difference was clearly visible even qualitatively, by eye (see CDF, Fig S2D). Speed measurements in Fig. 1 were used to simply report a mean speed and standard deviation, and the number of measurements are shown to allow reader interpretation. Recirculation of jasplakinolide-stabilized bundles (Fig. 3C, S4) were used to provide a description of bundle speed and its oscillatory nature, and the number of measurements are shown to allow reader interpretation. The characterization of the frequency of gliding modes (Fig. S1, S8) were used to show a significant increase in bidirectional gliding with increasing jasplakinolide concentration. p-values for an ANOVA test on this metric are shown with Fig. S8 in the SI, and the sample sizes used allow for highly significant pairwise differences between conditions.
Data exclusions	For speckle imaging experiments, we excluded cells with a fluorescent dye labeling density that was too low or too high to visualize and distinguish individual speckles. For analysis of speckle movement, we only analyzed tracks that persisted for at least 5 frames (0.43 seconds).
Replication	For measurements of the velocities of actin or myosin molecules or actin protrusions, measurements were replicated in 6-18 independent cells per condition. For measurements of the frequency of cell gliding behaviors, 45-156 gliding events were measured from 3 independent rounds of experiment. As discussed in detail in the methods section on jasplakinolide treatment, recirculation of actin bundles (Fig. 3c, S4) is very sensitive to time and concentration of jasplakinolide treatment. Beyond 30 min of treatment, protrusions are stationary, as previously reported, likely because bundles grow long enough to reach back from the conoid into the cell body, preventing movement. We find this an interesting biological result and discuss this interpretation in the manuscript.
Randomization	Cells in experimental groups were random subsets of the same cell stock. Cells were settled into imaging dishes or wells; individual wells or fields of view were chosen at random for each experimental group.
Blinding	One experimental measurement where this kind of group allocation could be relevant are the single-molecule speed comparisons in Fig. S2. In that case, molecule detection and tracking was done by an automated algorithm (u-track, Danuser lab), which was "blind" to which group it was analyzing. The other is the analysis of motility modes across jasplakinolide concentrations (Fig. S8). Here, movies for different jasplakinolide concentrations were blinded during analysis (information about concentration was not shown) and analyzed in a random order.

Reporting for specific materials, systems and methods

We require information from authors about some types of materials, experimental systems and methods used in many studies. Here, indicate whether each material, system or method listed is relevant to your study. If you are not sure if a list item applies to your research, read the appropriate section before selecting a response.

Materials & experimental systems

n/a	Involved in the study	
☐	☒	Antibodies
☐	☒	Eukaryotic cell lines
☒	☐	Palaeontology and archaeology
☒	☐	Animals and other organisms
☒	☐	Clinical data
☒	☐	Dual use research of concern

Methods

n/a	Involved in the study	
☒	☐	ChIP-seq
☒	☐	Flow cytometry
☒	☐	MRI-based neuroimaging

Antibodies

Antibodies used

Mouse anti-tubulin monoclonal antibody DM1alpha (Sigma T6199; diluted 1:500), anti-mouse IgG secondary antibody conjugated to Alexa Fluor 488 (Cell Signaling 4408S, diluted 1:500).

Validation

This tubulin antibody has been validated by the manufacturer for this application (immunostaining) and used extensively by the microtubule research community. In this work, it was used to label the beautiful subpellicular microtubules of Toxoplasma gondii, which have a very well-defined organization - which was clearly recognizable in our imaging.

Eukaryotic cell lines

Policy information about cell lines and Sex and Gender in Research

Cell line source(s)

Toxoplasma gondii tachyzoite strains (sex and gender not relevant) were made from the Toxoplasma gondii type I RH hxgprt ku80 strain, which was a gift of John Boothroyd and his group. Human Foreskin Fibroblasts were obtained from the neonatal clinic at Stanford University following routine circumcisions that are performed at the request of the parents for cultural, health, or other personal medical reasons (i.e., not in any way related to research). These foreskins, which would otherwise be discarded, were fully deidentified and therefore do not constitute human subjects research.

Authentication

Cell lines were not authenticated by external/official means.

Mycoplasma contamination

Cell lines (human foreskin fibroblasts) tested negative for mycoplasma contamination.

Commonly misidentified lines
(See ICLAC register)

No commonly misidentified cell lines were used.
